# Supplementary material for: Treatment decision making (TDM): a qualitative study exploring the perspectives of patients with chronic haematological cancers
Source: BMJ Open. 2022 Mar 29;12(3):e050816. doi: 10.1136/bmjopen-2021-050816 (PMC8966575; doi:10.1136/bmjopen-2021-050816)
Supplement: Supplementary data [file bmjopen-2021-050816supp001.pdf]

Supplementary file 1: Topic guide for interviews with patients and relatives

Focusing on information and decisions at key states (diagnosis, W&W, treatment) and progression through states.

Information

- How important is it to you that you receive information about your cancer? *(why is that?*)
- How do you feel about the information given to you at diagnosis/start of treatment?
- How do you feel about getting information from HCPs more generally? *(time constraints; overwhelming; difficult to understand/take in; use of language/terminology)*
- Do you feel the information given applies specifically to you? *(personalized, tailored, specific)*
- How healthcare practitioners (HCPs) ascertain your information needs?
- Is the information you received explained in a way you can understand? *(technical language; too detailed; not detailed enough)*
- What do HCPs do to check if you understand the information they give you?
- How do you feel about asking questions? Are your questions always answered?
- Do you feel that your information needs are usually met? What worked well and could have been better? *(diagnosis; treatment initiation/cessation - examples)*
- What do you think about the timing of information from HCPs? When is the right time? *(at diagnosis; during clinic appointments; when disease status changes; at other times)*
- How do/did you feel about discussing the risks/benefits of different treatments with HCPs?
- How do you feel about discussing prognosis? *("a statement about expectations that refers to the likely course of the cancer and/or outcome") (want to know/not; timing; language)*
- What strategies do you use to absorb information? (*in general, how bad news is processed*)

Treatment decisions

- How do you feel about being involved in decisions with HCPs about your treatment?
- Have you been asked you if you want to be involved in decisions about treatment?
- Do you want to be involved in decisions? *(preference for patient only; clinician only; patient/clinician)*
- What should be considered during treatment decision making? *(effectiveness of treatment; side effects; prognosis; patient goals, values, preferences; impact on quality of life)*
- What might make it easier or harder for you to be involved in making decisions about your treatment? *(time; style of communication; how information is conveyed; explanations)*
- Are there particular time-points when it is harder to be involved in making decisions about treatment? *(diagnosis; treatment initiation/change; treatment cessation)*

Practical issues

- How do you feel about the amount of information you get? *(prefer more/less; overloaded; struggle to absorb)*
- What do you want to know/know more about? *(investigations; treatments; prognosis; side effects; QoL)*
- Where/who do you prefer to get information; why? *(Internet; doctors/nurses; family; leaflets; support group)*
- What do you think about different sources of information? *(credibility, ability to judge)*
- How do you prefer to see information about risks and benefits; why? (w*ords/numbers; figures/percentages; diagrams/graphs)*
