# Supplementary material for: Treatment decision making (TDM): a qualitative study exploring the perspectives of patients with chronic haematological cancers
Source: BMJ Open. 2022 Mar 29;12(3):e050816. doi: 10.1136/bmjopen-2021-050816 (PMC8966575; doi:10.1136/bmjopen-2021-050816)
Supplement: Author's manuscript [file bmjopen-2021-050816.draft_revisions.pdf]

BMJ Open

BMJ Open is committed to open peer review. As part of this commitment we make the peer review history of every article we publish publicly available.

When an article is published we post the peer reviewers' comments and the authors' responses online. We also post the versions of the paper that were used during peer review. These are the versions that the peer review comments apply to.

The versions of the paper that follow are the versions that were submitted during the peer review process. They are not the versions of record or the final published versions. They should not be cited or distributed as the published version of this manuscript.

BMJ Open is an open access journal and the full, final, typeset and author-corrected version of record of the manuscript is available on our site with no access controls, subscription charges or pay-per-view fees (http://bmjopen.bmj.com).

If you have any questions on BMJ Open's open peer review process please email info.bmjopen@bmj.com

BMJ Open

Treatment decision making (TDM): a qualitative study exploring the perspectives of patients with chronic haematological cancers.

Journal:	*BMJ Open*
Manuscript ID	bmjopen-2021-050816
Article Type:	Original research
Date Submitted by the Author:	02-Mar-2021
Complete List of Authors:	McCaughan, Dorothy; University of York, Health Sciences<br>Roman, Eve; University of York, Health Sciences<br>Smith, Alexandra; University of York, Health Sciences<br>Patmore, Russell; Castle Hill Hospital, Queens Centre for Oncology<br>Howell, Debra; University of York, Health Sciences
Keywords:	Myeloma < HAEMATOLOGY, Leukaemia < HAEMATOLOGY, Lymphoma < HAEMATOLOGY, QUALITATIVE RESEARCH, Organisation of health services < HEALTH SERVICES ADMINISTRATION & MANAGEMENT, CHEMOTHERAPY

SCHOLARONE™
Manuscripts

I, the Submitting Author has the right to grant and does grant on behalf of all authors of the Work (as defined in the below author licence), an exclusive licence and/or a non-exclusive licence for contributions from authors who are: i) UK Crown employees; ii) where BMJ has agreed a CC-BY licence shall apply, and/or iii) in accordance with the terms applicable for US Federal Government officers or employees acting as part of their official duties; on a worldwide, perpetual, irrevocable, royalty-free basis to BMJ Publishing Group Ltd ("BMJ") its licensees and where the relevant Journal is co-owned by BMJ to the co-owners of the Journal, to publish the Work in this journal and any other BMJ products and to exploit all rights, as set out in our licence.

The Submitting Author accepts and understands that any supply made under these terms is made by BMJ to the Submitting Author unless you are acting as an employee on behalf of your employer or a postgraduate student of an affiliated institution which is paying any applicable article publishing charge ("APC") for Open Access articles. Where the Submitting Author wishes to make the Work available on an Open Access basis (and intends to pay the relevant APC), the terms of reuse of such Open Access shall be governed by a Creative Commons licence – details of these licences and which Creative Commons licence will apply to this Work are set out in our licence referred to above.

Other than as permitted in any relevant BMJ Author's Self Archiving Policies, I confirm this Work has not been accepted for publication elsewhere, is not being considered for publication elsewhere and does not duplicate material already published. I confirm all authors consent to publication of this Work and authorise the granting of this licence.

For peer review only

TITLE: Treatment decision making (TDM): a qualitative study exploring the perspectives of patients with chronic haematological cancers

AUTHORS: Dorothy McCaughan, Eve Roman, Alexandra Smith, Russell Patmore, Debra Howell

CORRESPONDING AUTHOR:

Debra Howell
Epidemiology & Cancer Statistics Group, Department of Health Sciences, Seebohm Rowntree Building, University of York, York, UK. YO10 5DD.
Telephone: +44 (0)904 321895
Email: debra.howell@york.ac.uk

CO-AUTHORS
Dorothy McCaughan
Epidemiology & Cancer Statistics Group, Department of Health Sciences, Seebohm Rowntree Building, University of York, York, UK. YO10 5DD.
Telephone: +44 (0) 904 321348
Email: dorothy.mccaughan@york.ac.uk

Eve Roman
Epidemiology & Cancer Statistics Group, Department of Health Sciences, Seebohm Rowntree Building, University of York, York, UK. YO10 5DD.
Telephone: +44 (0) 904 321882
Email: eve.roman@york.ac.uk

Alexandra Smith
Epidemiology & Cancer Statistics Group, Department of Health Sciences, Seebohm Rowntree Building, University of York, York, UK. YO10 5DD.
Telephone: + 44 (0) 904 321884

Russell Patmore
Queens Centre for Oncology and Haematology, Castle Hill Hospital, Cottingham, UK.
Telephone: +44 (0) 482 604444
Email: Russell.Patmore@hey.nhs.uk

KEYWORDS: leukaemia, lymphoma, myeloma, treatment decision making, qualitative research

WORD COUNT: 4895, including quotations embedded in text, excluding standalone quotations

For peer review only - http://bmjopen.bmj.com/site/about/guidelines.xhtml

ABSTRACT

Objectives

Haematological malignancies are the fifth most common cancer in the UK, with chronic subtypes comprising around a third of all new diagnoses. These complex diseases have some similarities with other cancers, but often require different management. Surgical resection is not possible, and while some are curable with intensive chemotherapy, most indolent subtypes are managed with non-aggressive intermittent or continuous treatment, often over many years. Little is known about the views of patients with chronic haematological cancers regarding treatment decision making (TDM), a deficit our study aimed to address.

Setting and design

Set within the Haematological Malignancy Research Network (HMRN: www.hmrn.org), an ongoing population-based cohort that provides infrastructure to support evidence-based research, HMRN data were augmented by qualitative information from in-depth interviews. Data were analysed for thematic content, combining inductive and deductive approaches. Interpretation involved seeking meaning, salience and connections within the data.

Participants

Thirty-five patients with four chronic subtypes: chronic lymphocytic leukaemia (CLL), follicular lymphoma (FL), marginal zone lymphoma (MZL), and myeloma. Ten relatives also took part.

Results

Five themes were discerned: 1. Preference for clinician recommendations; 2. Factors implicated in patient involvement in TDM; 3. Perceptions of proactive/non-proactive approaches to TDM; 4. Experiences of TDM at various points in the disease trajectory; 5. Support from others. Our principal finding relates to a strong preference among interviewees for treatment recommendations from haematologists, based on trust in their expertise and perceptions of empathetic patient-clinician relationships.

Conclusion

Interviewees wanted to be involved in TDM to varying extents, contingent on complex, inter-related factors, that are dynamic and subject to change according to differing clinical and personal contexts. Patients may benefit from clinicians assessing their shifting preferences for involvement on multiple occasions. Strong preferences for acceptance of recommendations was associated with cancer complexity, trust in clinician expertise, and positive perceptions of patient-clinician relationships.

Strengths and limitations

For peer review only - http://bmjopen.bmj.com/site/about/guidelines.xhtml

- Set within the infrastructure of an established population-based study, this is (to our knowledge) the first UK study to specifically explore TDM involvement in patients with haematological malignancies.
- Interview data supplemented robust clinical information routinely collected as part of the broader cohort.
- The sample size (35 patients) enabled in-depth exploration of the research questions, enhanced by contributions from relatives/carers.
- Purposive sampling ensured inclusion of individuals with differing demographic and disease profiles, diagnosed at different time points on the clinical pathway.
- The views of participants are unlikely to reflect those of the entire patient population, and dedicated studies of people from black and minority ethnic backgrounds and those with low literacy levels are required.

For peer review only

For peer review only - http://bmjopen.bmj.com/site/about/guidelines.xhtml

TITLE: Treatment decision making: a qualitative study exploring the perspectives of patients with chronic haematological cancers

BACKGROUND

Haematological malignancies are the fifth most common cancer in the UK [1-3]. They are complex diseases, and although they have some similarities with other cancers, they often require different management. Surgery is not an option, and while some subtypes are curable with intensive chemotherapy and periods of hospitalisation, other more chronic subtypes are not. These more indolent malignancies, which account for around 30% of all newly diagnosed haematological cancers [6, 7], can, however, often be controlled for long periods; typically (but not always) following remitting/relapsing pathways wherein periods of chemotherapy are interspersed with active monitoring, known as Watch and Wait (W&W) [4, 5]. Haemato-oncology is a rapidly changing field, and the past decade has seen the development of innovative treatment regimens that include targeted therapies, along with new and established chemotherapies, radiotherapy, and haematopoietic stem cell transplantation (SCT) [8]. While obviously beneficial, these developments increase treatment decisional complexity for clinicians and patients [9].

Shared decision making (SDM), seen as a hallmark of quality care, is part of a broader concept of patient-centred care which considers individual preferences, needs, and values, with the aim of ensuring patient values guide all clinical decisions [10-12]. Steps integral to SDM include the clinician informing the patient of treatment options and the need for a decision; discussion between patient and clinician of each option; and the clinician supporting the patient to consider each option, before reaching an informed decision [13-15]. Elicitation of patient preferences is considered central to effective SDM [16, 17]. When multiple treatment options exist, each of which may be associated with different risks, benefits and quality of life implications, adherence to SDM steps can result in a decision that is optimal for the individual patient (and their relatives/carers). If evidence for a specific treatment is strong, the clinician may make a recommendation, informing the patient of their reasoning, which the patient may accept or decline [18, 15]. Audiotaped consultations between 236 patients and 40 haematologists in the United States (US) [19] revealed that patient preferences were not commonly elicited, and that treatment recommendations were provided by haematologists in 97% of consultations. More recent studies, (mainly surveys), indicate increasing desire amongst patients with haematological cancers for involvement in treatment decisions [20-22], but highlight dissatisfaction with information received. A recent thematic review [15] of 18 haematological studies, indicates three critical, but modifiable, barriers to patient centred communication, a prerequisite for SDM: insufficient information exchange, treatment goal

For peer review only - http://bmjopen.bmj.com/site/about/guidelines.xhtml

misalignment, and discordant (patient preferred and actual) role preferences in decision making. Despite the recognised need for qualitative research to better understand and contextualise preferences for involvement in decision making amongst haematology patients [15], few qualitative studies have been conducted.

Our study was designed to investigate the perspectives of patients with one of four haematological subtypes: chronic lymphocytic leukaemia (CLL), follicular lymphoma (FL), marginal zone lymphoma (MZL), and myeloma. Specifically, we aimed to explore patients' understanding and experiences of involvement in treatment decision making (TDM), and to identify factors promoting or impeding this process. This study constitutes one strand of a larger programme of work designed to provide evidence-based information about the management and experiences of the general population of patients with chronic haematological malignancies.

METHODS

Methods are reported in accordance with the Consolidated Criteria for Reporting Qualitative Research Checklist [23].

2.1 Study design

A qualitative, descriptive study [24, 25], utilising semi-structured in-depth interviews.

2.2 Sample and setting

The study was conducted within the UK's Haematology Malignancy Research Network (HMRN: www.hmrn.org), a unique collaboration between university academics, National Health Service (NHS) clinicians, and patients and carers, that facilitates research using various methods, with the purpose of generating evidence to underpin improvements in clinical practice. Detailed information about HMRN's configuration, methods and ethical approvals has been published elsewhere [6,7].

Sampling in qualitative research aims to acquire information that is useful for understanding the complexity, depth, variation, or context surrounding a phenomenon [26, 27]. We therefore aimed to capture a broad range of diverse experiences; initial criteria included proximity to the median diagnostic age for each disease subtype, with variation by gender, ethnicity and postcode, as well as time since diagnosis. Over time, our sampling strategy evolved to purposively select individuals across broader demographic categories (for example, those relatively young when first diagnosed), and to ensure inclusion of participants at different time points on the clinical pathway. To appreciate

For peer review only - http://bmjopen.bmj.com/site/about/guidelines.xhtml

the role of caregivers, patients were asked to invite a relative to contribute to the interview, if they wished. Details of the study sample can be seen in Table 1.

2.3 Data collection

Using existing links with NHS teams, initial checks ensured patients were alive and well enough to participate. Potential participants were sent information about the study, and a letter inviting them to take part in an interview, with a reply slip and pre-paid envelope. The researcher's contact details and free-phone number were included so that patients could discuss the study before deciding whether to take part. Thirty-five interviews were conducted (DM) between February and October, 2019; and ten relatives participated. The majority took place privately, in patients' homes and lasted around 40-90 minutes. Interviews were digitally recorded and transcribed verbatim, checked for accuracy and anonymised. Recordings and transcriptions were stored in accordance with legally required data protection standards and ethically approved practices. Interviewing continued until it appeared no new information was forthcoming, a signal that data saturation was likely achieved [28], and the recruitment end-point occurred when preliminary analysis indicated patterns and themes with sufficient data [29].

Interviews were directed by a semi-structured topic guide **(Appendix 1)** based on research literature and input from clinicians, and piloted with 2 patients (from a haematology cancer support group) to check comprehensiveness and comprehensibility. The guide was modified over time to include new lines of inquiry, and was used flexibly to allow patients to "tell their story" from diagnosis onwards.

2.4 Data analysis

The analytic approach adopted was qualitative description [24], based on thematic content analysis [30]. Qualitative description research seeks to discover and understand a phenomenon, a process, or the perspectives and worldviews of the people involved [31-33]. Analysis was undertaken by two members of the research team (DM, DH), both experienced in qualitative methods in applied health services research and haematology. Interviews were summarised through dynamic engagement with the dataset, while staying close to participants' accounts [24]. Our aim was to transfer the 'raw' data into a coherent depiction of the phenomena under scrutiny [34]. Guided by the research questions, our analysis balanced both inductive and deductive orientations [35]. We familiarised ourselves with the content of the transcripts to identify initial codes (units of meaning) and themes. These were modified and expanded during an interactive and reflexive process of 'interrogating' the data, in the search for common patterns and 'deviant' or 'negative' cases not supporting, or appearing to

For peer review only - http://bmjopen.bmj.com/site/about/guidelines.xhtml

contradict, patterns or explanations emerging from data analysis [36]. Data were then summarised and compared, within and between cases. Our analysis therefore facilitated data synthesis and interpretation, enabling a detailed and nuanced account of the findings [34]. Analytical rigour was promoted through reflective notes and memos, and discussion of disagreements helped refine the analysis [37].

2.5 Ethical considerations

Research projects set within HMRN's infrastructure and collecting supplementary data require ethical approval, which was granted by the London, City and East committee (REC:16/ LO/0740). Assurances of confidentiality and anonymity were given to participants and written consent was obtained from patients and relatives. During interview, a (small) number of patients became upset while reflecting on their cancer and its progression. Although these patients were asked if they wanted to pause or discontinue the interview, all wished to continue, some commenting afterwards that the discussion had helped clarify their thinking about their disease.

2.6 Patient and Public Involvement

Patient public involvement (PPI) is integral to HMRN and lay-individuals are routinely involved in all research activities. For this particularly study, patients and relatives were involved in prioritising aims, preparing the funding application, attending programme steering committee meetings, and the dissemination of findings.

RESULTS

Thirty-five patients (19 male, 16 female) were interviewed, 10 accompanied by relatives (spouse/partner or other family member) who contributed to the interview. Most were aged 50-70 years; 32 lived with a spouse/partner or other family member and three lived alone. Ten patients had CLL, eight FL, twelve myeloma and five MZL. Prior to interview, patients had experienced different treatment pathways, according to diagnosis and disease progression; some had started and remained on W&W, others had started treatment, and a further group had experienced multiple lines of chemotherapy before progressing to stem cell transplant. Patient characteristics and individuals' treatment pathways, ascertained from HMRN routine data collection, and patient self-report, can be found in Table 1.

For peer review only

Table 1. Characteristics of interviewees

[1] CLL – chronic lymphocytic leukaemia; FL – follicular lymphoma; SMZL – systemic marginal zone lymphoma; EMZL – extra-nodal marginal zone lymphoma.
[2] Who the patient lived with at the time of interview.
[3] HMRN: Haematological Malignancy Research Network
[4] Chemotx = Chemotherapy; HPE = H. Pylori eradication; Radiotx = Radiotherapy; SCT = Stem cell transplant (all autografts); SCH = Stem cell harvest (shown as SCT did not take place – risk considered too high by medical staff/patient).
[5] Does not include supportive care (e.g. blood product transfusions, plasma exchange, bisphosphonates, cell mobilization products)
[6] P2 & P3 were diagnosed before the start of HMRN, but volunteered for interview via a support group. Data presented were collected during the interview.

For peer review only

The following 5 themes were identified: 1. Preference for clinician recommendations; 2. Factors implicated in patient involvement in TDM; 3. Perceptions of proactive/non-proactive approaches to TDM; 4. Experiences of TDM at various points in the disease trajectory; 5. Support from others.

Theme 1: Preference for clinician recommendations

Most of the patients in interviewed in our study said they wished to be informed about, and given the opportunity to discuss, treatment benefits, risks and outcomes with their doctor, while expressing a preference for the clinician to make a recommendation. Some said they wanted their consultant to explain the rationale for recommendations, while others said they weren't interested, that they just wanted *'to get it* [treatment] *over and done with'*.

'they spoke to me so much, I feel involved… I would hate for them to say, you're going to do that, with no explanation…and that doesn't happen. When I have treatment, I am told why.' (P9)

'I wanted their expertise and their guidance…I felt very involved but I didn't necessarily feel I should be making the ultimate decisions… I wanted them to make the decisions…' (P19)

I would just do what they recommend really' (P23)

'I just like the person telling me what I've got and what they're going to do' (P27)

Preferences for clinician recommendations were said to be based on patients' respect for haematologists' clinical knowledge and expertise, trust in their professional judgement, and faith that they would have the patient's best interests at heart.

'I do have a respect for medical people because they've done all the training, so I would hope they would say… well we think this is probably the best way forward, because I think, well, how would I know?' (P26)

'I've got total faith in what they are doing…I am certainly not capable of making a medical decision on my behalf' (P21)

'the team are making that decision for me, and they're working for me with my best interests at heart' (P13)

For peer review only - http://bmjopen.bmj.com/site/about/guidelines.xhtml

Trust in individual haematologists was strongly linked to patient perceptions of (mainly) excellent patient/doctor relationships, characterised by consultants' willingness and ability to: demonstrate empathy (by helping patients feel at ease, and valued as individuals, *'not just a number'*); tailor information to match individual requirements; listen and respond to questions and concerns; initiate and engage patients in open and frank discussion; and impart hope and some positivity when things are not progressing well.

'she [haematologist] explained everything…I could understand everything…there was no stress involved…she seems to be able to ask the right questions and she takes it all in…she tends not to write down until we've finished talking…we just have a chat basically…she is so warm and pleasant, smiling, we have a laugh…I mean you come away feeling elated rather than 'phew' (P10)

'maybe they just think well, if somebody is not asking at the moment, we won't say, because this is too much information. Maybe they take their lead from where you're at…' (P27)

'giving time for people to actually speak…especially when people are ill, to think about things before they ask questions…so listening is probably at the top of the list' (P29)

'let's have everything as open as possible so that everybody who's in the equation knows what's going on or what possibilities are out there for treatments' (P14)

'I was a bit frightened but she said, if you do exactly as we've planned…you will come out of it…I'm so certain of that. Now, when any consultant tells you that, it sort of lifts you' (P9)

Trust was said to be enhanced when patients knew that treatment discussions had included members of the wider haematology team, as happened within the context of multidisciplinary team meetings, if their consultant had sought a second opinion from haematologists specializing in the patient's condition, and when the patient was aware that they were receiving treatment in a recognised centre of excellence.

'these people [haematologists] are much more expert than I'll ever be…the consultant that is making the decision, it's his team. You've been talked about, so it isn't one person making that decision…in the background there's quite a lot of people that are involved and I find that really, really soothing' (P13)

'it's reassuring that it is not just one person making that decision…when he [haematologist] decided I needed to start chemo, he did say he had actually spoke to a colleague of his at [place] University…I

For peer review only - http://bmjopen.bmj.com/site/about/guidelines.xhtml

think he was a professor…and told him my symptoms, and the professor had also said, well, yes, I think it's time to start treatment…' (P4)

I knew they were a centre of excellence and they had very high standards…so, I just felt very…secure' (P19)

Interestingly, some people revealed awareness of 'their' consultant's clinical and academic credentials.
'he [patient's consultant] was the lead consultant for the team so I thought, well that is a good recommendation' (P24)

'and Prof [name] who I've seen perhaps 4 times and everyone wants to see Prof [name], because he's got the biggest brain [laughs] (P28)

Only one study participant (P3, with CLL) mentioned having themselves sought a second opinion, commenting that far from taking offence, his consultant assisted him in identifying appropriate specialists to contact.

'he [haematologist] didn't take that as a personal insult…you feel more confident…going to see one of the top specialists [second opinion]…you're going to see a range of experts who have agreed this is the best option…you are being backed by a range of experts' (P3)

Theme 2: Factors implicated in patient involvement in TDM
The extent to which participants' in our study said they were involved in TDM appeared to vary according to a range of inter-related individual *('everybody is different')* and contextual factors, including decision complexity; individuals' ability and desire to access, interpret, and retain information about their cancer; personal preferences and values; patients' physical and/or emotional state, and coping mechanisms; and the level of support from others. These factors are summarised in Figure 1. While some patients wanted to hand over much, or all, responsibility to clinicians, others took steps to enable participation in discussions; many interviewees clustered somewhere between, reporting varying levels of engagement/non-involvement, dependent on circumstances.

[Individual differences]: *'every patient is different…there probably are patients who just rely on their specialist…most are happy to just 'get on with it gov', whereas I was always asking questions and that's what I would recommend to other patients, is try to understand and be proactive… that way at least when [there's] any option to decide if I can feel confident that's the best available for me'* (P3)

For peer review only - http://bmjopen.bmj.com/site/about/guidelines.xhtml

[Decisional complexity]: *'in an ideal world it would be myself, me and the doctors [who share TDM] because to me, they've got so much knowledge about this thing that I don't know anything about… I'd just like to have something [simple] like, Oh, you've got appendicitis'…but I'm not in that situation. It is complex…they don't know all the answers…because there's different types of it [myeloma] and I don't fully get the reasons why everyone reacts differently [to treatment]'* (P35)

[Information access]: *'I'm not an internet person…it annoys me, the internet'* (P15)

[Information understanding]: *'Not everyone is equipped to sort of read some of the literature because…it is a little bit challenging…and time consuming'* (R, P6)

[Information retention]: *'We were given some leaflets…but I'm a bit fuzzy…since I've had my cancer my memory has gone to shot'* (P16)

[Physical/emotional state]: *'I was so rock bottom, I guess I just went along with it all…they knew what they were doing'* (P8)

[Coping mechanism]: *'there are some people who want to know every detail about every treatment and how it affects them…I'm scared to do that'* (P35)

[Personal preference (for proactive role)]: *'PatientView [portal for accessing patient electronic record]…that's fantastic because I can see things, so your platelets, white [blood cells]…haemoglobin…it's a fantastic bit of information for me prior to my consultant appointment…having that extra information, for me, is very valuable…I can point out…to the consultant…so what's this about…you know, why's that gone up?'* (P28)

[Personal circumstances]: *'your mind is concentrating on other things...is it life threatening…am I going to be able to work anymore…have I got to retire…you've got the financial aspects…making sure your family is looked after…your brain is working on so many different levels…you tend to accept what's [treatment] being given'* (P16)

[Level of support]: *'that's what I miss, because I have to do all my own research…and sometimes I think I just wish I had that person… [for support]'* (P33)

Theme 3: Perceptions of proactive/non-proactive approaches to TDM

During interview, a number of patients articulated views relating to proactive involvement in TDM, within the context of their stated preference for acceptance of haematologists' recommendations,

For peer review only - http://bmjopen.bmj.com/site/about/guidelines.xhtml

while others described factors likely to impede or diminish active engagement in TDM. Factors associated with proactive and non-proactive approaches to decisions are summarised in Figure 2.

Those who chose to be proactive described the need for certain resources (internet, time), skills (retrieving/interpreting relevant information) and a high level of personal commitment. This group also tended to access information from many sources, including UK charities (for example, Myeloma UK and CLL Support Association), national and local support groups, on-line patient forums (mainly US based), clinical nurse specialists (CNSs), and peer reviewed articles. Some patients described how internet access was essential, along with the ability to distinguish between *'authoritative'* and *'dreadful'* websites. Taking time to read about blood/lymphatic systems was seen as a necessary pre-requisite for understanding specific diseases. Patients who sought in-depth information from journal publications tended to have some prior knowledge which facilitated their understanding, up to the point when they encountered *'the buffers'* of what they could comprehend.

'*I did intelligent searches…I was confident and used to reading science papers…'* (R, P6)

'I had read on the internet all about these different prognostic indexes and stuff…when I was told prognosis was 5 years…I already knew what prognosis meant' (P34)

'I'd be looking at Lancet type papers…PubMed' (P24)

'it turned out the free light chains had rocketed… so that ruled out second stem cell transplant' (P18)

'*it's good to know statistics…I wanted to know about survival figures…I realised that I was looking at the population as a whole, but then I thought I'd look at younger people…'* (P3)

'it's challenging because you will reach the buffers at some point, when you think, that's just a little too difficult to understand' (R, P6)

Proactive patients described preparing for consultations. Despite busy clinics, patients said doctors rarely made them feel time was constrained, usually invited questions, and took time to respond. Some patients prepared questions beforehand, a few noted the consultant's response(s), with some having relatives acting as 'scribe'. Keeping a record or graph of blood test results, used as a trigger for questions, and maintaining a diary of disease progress and treatments were common. These patients sought results from investigations (blood tests, bone marrow biopsies, X-rays, CT scans, etc.) and often asked consultants to explain their significance. They also read about the risks, benefits and possible side effects of treatments, to equip themselves to engage in further discussion. Being prepared to 'speak up' during consultations was said to be important, though was recognized

For peer review only - http://bmjopen.bmj.com/site/about/guidelines.xhtml

to be easier for some than others: *'you have to be reasonably assertive, which is maybe not my strength, but I force myself in those situations where I know how important it is'*. Only one patient (P6) and their spouse (R,P6) reported 'speaking up' to express a preference for a treatment other than the one offered by their consultant, based on their own *'research'*, that suggested it would be less detrimental to the patient's quality of life: *'we knew the gold standard was treatment with Fludarabine, FCR as they call it, it really wasn't recommended for people aged over 65, but nonetheless it was on offer to us…we actually then chose the Bendamustine route…the gentle one…which was a good alternative…we'd done our research, we made an intelligent decision'* (R, P6).

Many respondents expressed little or no desire to engage in active TDM, preferring to rely on clinicians' expertise, as reflected, for example, in the following comments: '*I don't want to go into details of things…the people at the hospital are there to help me…I put my absolute trust in them'* (P10); *'I went along with everything that I was asked to do because I have complete confidence in how they were handling the situation for me'* (P14).

Factors that could impede patients adopting a proactive approach included difficulties accessing and/or fully understanding information, a disinclination to dwell on disease progression and prognosis *('in denial')*, or patients feeling unwell, anxious, overwhelmed, or unsupported.

'I've got a little tablet but I very rarely use it. I don't like to, I'm not computer literate' (P30)

'the specialist at the hospital said, it's only forty-four, which meant nothing to me, forty-four what? She didn't say what and I never asked her.' (P31)

'sometimes they come out with all these big words and then you think, I'm not sure what that word is' (P24)

'I have glanced through it [booklet about myeloma] but it's a bit too high a level…it needs to be basic' (P21)

'I saw this research about myeloma…and when I read the title I wasn't sure whether it was going to be that helpful, because I didn't understand what it was really…' (P35)

For peer review only - http://bmjopen.bmj.com/site/about/guidelines.xhtml

'psychologically, I brush it under the carpet a bit because I know it [chemotherapy] is not imminent' (P5)

'I've just gone back to being anxious again…it's just horrible being in this position where you know it's [paraprotein level] is creeping up' (P35)

'I am strong, you know, but it just becomes too much… I want somebody just to hold my hand and go, I'm going to sort that for you.' (P33)

Theme 4: Experiences of TDM at various points in the disease trajectory

Patients' accounts indicated that the potential and desire for involvement in TDM could vary or indeed be curtailed at different points in the disease trajectory. Most participants described feeling deeply shocked, upset and anxious when first informed of their diagnosis (*'like a huge bombshell'*), to the extent that they could not absorb what was being said to them or think of questions to ask (*'it's as if your brain switched off'*), reactions likely to compromise ability to participate in discussions about treatment options.

'we were floored…[by diagnosis]…it hit us out of the blue…you can't think of questions in that short space of time…we were speechless…we just sat there in shock…you hear the word cancer…'' (P32)

'it was a shock when he mentioned the word cancer…it just sort of shut me down…he was willing to give me information but at that time, I just couldn't process it' (P4)

Some patients on W&W who subsequently required treatment, reported little or no involvement in TDM, as the treatment given was *'automatic'* or *'standard'* chemotherapy; some of these participants said they would have welcomed the opportunity to discuss the proposed treatment with family members, and consider whether or not to accept it; a few recalled their consultant strongly recommending that they accept the treatment offered.

'It was a fait accompli really' (P15)

'I was told that I had to have chemotherapy, 6 chemotherapies…one every 3 weeks and radiotherapy after that' (P8)

For peer review only - http://bmjopen.bmj.com/site/about/guidelines.xhtml

For peer review only

'I was very worried and scared and thought I might choose not to have the treatment…because I felt physically very well…why subject myself to being ill' (P1)

'[consultant said] "I must tell you that even though you don't want to go on chemo….my recommendation is that you do"' (P9).

Opportunities for involvement in decision making appeared circumscribed when urgent treatment was required. Examples came from P19, who was admitted to hospital acutely ill and subsequently diagnosed with FL *'a very unusual lymphoma'* (P24); and from P21, with myeloma. In these instances, each patient referred to TDM occurring at speed, largely without their involvement, as they relied on the expertise of the clinical team to make the *'best'* decisions on their behalf.

'There weren't really any options…it was a case of this is what is best for you… I didn't have time to think about it… it [the cancer] was so advanced…they decided… I don't think I was really involved in that…I wanted them to guide me and make the decisions' (P19)

'it was a very unusual type of lymphoma…there was a huge team involved in it all and even throughout their decision making they changed from what were originally going to do, which was radiotherapy...they were just going to blitz it, but my kidney would have been in the way, so they then decided to go down the avenue of chemotherapy and the monoclonal…' (P24)

'they [doctors] decided on stem cell transplant straightaway' (P21)

Disease progression resulted in some patients with myeloma feeling overwhelmed when faced with difficult treatment decisions, and unable to choose between options. Factors compounding this included the intensive nature of proposed treatments (such as stem cell transplant) and their impact on quality of life; the limited "returns" that some treatments seemed to offer, compared to the consequences of associated risks, such as infection; and the uncertainty and unpredictability of outcomes.

'Honestly, my head is exploding with all this…it's just like a big crushing thing to me…I think I am quite strong but this is doing me in' (P35)

'the big one [decision] was the second stem cell transplant…I was really struggling to make the decision as to whether I wanted to go for it' (P18)

For peer review only - http://bmjopen.bmj.com/site/about/guidelines.xhtml

'they [doctors] sort of said, well the average remission after the stem cell transplant is, I think either 12-24 months, or 18-24 months, something like that, and there was I thinking, right I'm going for the 10-year option, so that was quite a shock' (P18)

'Myeloma is a very individual disease. You get the same treatment, same, same this, same that but you have different outcomes and things.' (P28)

Theme 5: Support from others

Our interviewees said that their relatives often accompanied them to clinical consultations, and were portrayed as playing an important, and sometimes crucial, role when treatment options were being discussed and considered. Patients benefitted from *'going over'* information from their haematologist with their relative following consultations, and discussing the details of any proposed treatment, side effects, and implications for quality of life on them, and possibly their family and friends. Some patients (for example, those who mentioned difficulties processing and retaining information) described relying heavily on support from relatives (spouse/partner/adult child/sibling) for all interactions with clinicians. Relatives' roles encompassed gathering and interpreting information, acting as a sounding board, and providing practical and emotional support to patients preparing for, and undergoing, treatment. Many patients and relatives used the pronoun *'we'* throughout the interview, though there was general agreement that the final decision of whether or not to accept a treatment, was/would be the patients. The importance of emotional support provided by a spouse/partner to patients experiencing anxiety and inner turmoil associated with diagnosis and TDM was repeatedly emphasised: '*she's [patient's wife] been with me at every step', 'you need somebody to be the rock…to take the strain off you'.*

Three study participants living alone commented that they often felt unsupported in relation to their cancer, feelings that were heightened when treatment decisions arose: *'I knew chemo was what I'd have to have but when he* [consultant] *told me, that shocked me, and I was so upset and scared…really scared'* (P4). In the absence of a spouse/partner they turned to friends or close family members for advice and support, but did not want to be a burden to them: *'I've got a very good friend…we've known each other 50 years…I would never have got through it without her…you need support…somebody talk to…but she has her own family…I don't like to be a burden to anybody'.* Two of the three participants without a spouse/partner mentioned seeking formal psychological support, so that they might have the opportunity to share and discuss their feelings and experiences.

For peer review only - http://bmjopen.bmj.com/site/about/guidelines.xhtml

Eight of the 35 participants had joined a formal support group. Reported benefits included hearing about up-to-date research findings from invited speakers (often clinicians), and having the opportunity to talk to other people about their experiences, which helped some patients think through advantages and disadvantages of their own options for treatment. Those who preferred not to join a group *('not for me'), ('I'm a very private person'),* often sought one-to-one support, through meetings with former patients arranged by clinicians, personal contacts with someone with a similar diagnosis, and/or via on-line patient forums. Further illustrative quotations linked to Theme 5 are presented in Box 1 below.

Box 1. Illustrative quotations linked to Theme 5: Support from others

'she [patient's wife] guided me…she would translate…she would talk to the ward people…' (P7)

'we'd come home and discuss it [information from the consultant] what did she say about so and so…having two sets of ears helped' (P11)

*'we were proactive and looked for information' (*R, P7)

'my daughter always takes notes...so when we come away we can go through them…they are quite happy with that…if I go on my own, I would retain some of it and I'd probably forget some of it…my daughter knows all about my treatment' (P9)

'we'd both be involved…it wouldn't be one partner on their own, it would always be the two of us involved together' (P21)

'my daughter… when they originally first started talking about the trial, my daughter went on-line' (P13)

'I don't know how I could have coped without him [husband]…'(P6)

'It's your [patient's] decision but we talk about it' (R, P22)

'he [husband] doesn't sway me, he leaves it very much up to me wouldn't persuade me…' (P1)

'I'm as involved as much as I can be but at the end of the day it's [patient's name]… he has to make the decision, I can't make it for him' (R, P31)

'she [partner] kind of left me to make the decision really, but we'd talked through it over a number of weeks' (P18)

DISCUSSION

This qualitative study provides new insights into patient perspectives of involvement in treatment decisions, their views on proactive engagement, and the role of others in supporting decision making. The findings reflect the broad array of interconnected mechanisms at play in shared

For peer review only - http://bmjopen.bmj.com/site/about/guidelines.xhtml

decision making [38], and its complex and dynamic nature [39-41]. The principal finding among our interviewees was the strong preference for treatment recommendations to be provided by haematologists, based on trust in their clinical expertise and perceptions of empathetic patient/clinician relationships. Most participants expected/wanted an explanation from their clinician about the rationale for treatment decisions, including details of possible risks and benefits, but did not wish, and/or felt ill-equipped, to make decisions on their own behalf. This finding does not align with some recent reports of the growing desire for involvement amongst patients with haematological malignancies [20-22], though seems unsurprising within the detailed contexts depicted by our interviewees. Participants with myeloma tended to experience decision making as challenging, struggling to weigh up the risks, adverse impacts on quality of life, and prognostic uncertainty associated with different treatments options; which left them inclined to follow clinician recommendations. Patients with FL also reported trusting clinicians to make decisions on their behalf, particularly when they were acutely ill at diagnosis (as may occur in some of these typically indolent cancers), and unable to participate in discussions. Likewise, participants with CLL were mainly inclined to entrust decisions to clinicians; however, some expressed dissatisfaction that they had not been given the opportunity to consider, and accept, or decline, treatment.

Drawing on results from their systematic review of the literature relating to physician views of SDM, Pollard et al. [42] comment that physicians tend to express support for SDM in situations where they do not feel strongly about one treatment alternative, but are less supportive of SDM in situations where compelling, or well-evidenced, clinical practice guidelines exist in favour of one treatment over another. In such instances, the decision is not one of selecting between options, but rather whether the patient chooses to accept or decline treatment. This review includes results from an interview study with 20 physicians working in five different settings [43], that found support for SDM was most common among those who had received communication skills training in this area; and Rocque et al. [44] suggest that multi-level education programmes, targeting patients with CLL and their clinicians, may improve patient participation in decision making.

Patient involvement in decision making has been linked to improved care experiences and better health outcomes, yet the desire for this has been shown to vary by individuals, number of treatment options and treatment certainty [45]. That patients with haematological malignancies, which are characterised by uncertain trajectories, indistinct transitions and prognostic uncertainty [46, 47, 7] and novel and evolving treatments, may prefer to defer decisions to specialists, whom they trust, is understandable. Our findings resonate with qualitative studies in Germany [48] and Denmark [49], that combine interviews with extensive observation of consultations with clinicians and patients

For peer review only - http://bmjopen.bmj.com/site/about/guidelines.xhtml

with cancer, and which show that most of the time physicians made treatment decisions alone, or with colleagues, with little patient involvement. None of our participants recalled clinicians formally eliciting their preferences regarding decision-making, as is recommended [50, 51], yet most felt as involved in this process as they wanted to be, through discussions of treatment options, and clinicians taking time to talk and listen to them, address their concerns, answer questions, and offer explanations. Patients in our study clearly felt strong bonds with their specialists, as noted elsewhere [47], arising from sustained contact in clinic or during hospital admissions. A meta-ethnography of quantitative and qualitative studies [52] underscores the importance patients place on being in a caring relationship with clinicians, which may preclude the need to seek detailed information.

Amongst our participants was a small number (n=5) who shared certain characteristics (male, (mainly) younger, educated to degree level, and highly motivated), and who had adopted a proactive approach to TDM. These individuals generally felt confident interpreting complex information, and were prepared to 'speak up' to obtain further explanation from clinicians; nonetheless, limits to understanding were perceived as constraining their ability to make fully informed decisions. Loh et al. [53] caution against predicting preference for decisional involvement of patients with haematological cancers, based on age or characteristics such as educational attainment, suggesting instead that this should be assessed periodically, as part of decision-making encounters.

While many respondents preferred little or no involvement in TDM, there were some for whose engagement was hindered by the provision of information that did not match their needs, leaving them feeling ill-equipped to deal with the complex nature of this material. Clinician assessment of individual health literacy (capacity to access, process and interpret information) is therefore an essential component of SDM, as is noted by others [15]. Providing patients with information that is comprehensible, tailored to their needs, and which does not overwhelm [54], can be challenging. Strategies for 'drip-feeding' [54] information, are to be preferred to a one-way-flow of information from clinician to patient ('broadcasting'), which is regarded as suboptimal [55]. Interestingly, insufficient time for information sharing and discussion during consultations was not generally perceived as a problem in our study, though is reported elsewhere [15, 48, 20].

During interview, patients often recalled feelings of profound shock on hearing their diagnosis, reactions that could act as a barrier to meaningful discussion about treatment. A qualitative study [56] including 32 patients with acute myeloid leukaemia revealed similar findings; highlighting the importance of clinicians managing the amount of information patients are ready to receive [54], both at diagnosis and other timepoints; for example, initiation of treatment after W&W.

For peer review only - http://bmjopen.bmj.com/site/about/guidelines.xhtml

The important role played by relatives and others in supporting patients during consultations when treatment options are discussed was very apparent in our study, reflecting results from a systematic review [57] of patient-physician-companion communication that shows companions as instrumental in information transfer and provision of emotional support. Unaccompanied patients in our study said they particularly benefitted from a nurse taking notes of what was said during consultation, and 'talking through' the record with them afterwards. Interviewees without a spouse/partner expressed concerns about burdening friends and family members with their needs for practical and emotional support. While some patients may be reluctant to broach the topic of their cancer with others, or find it difficult to talk about it, they are likely to benefit from discussing treatment options/decisions with someone close to them, who is familiar with their personal preferences and circumstances; haematology doctors and nurses could take time to help patients identify such individuals, and encourage patients to draw on their support.

Coping with disease progression and prognostic uncertainty was said to be particularly difficult by our participants with myeloma. Treatment with SCT (or the newer CAR T-cell therapy) can affect patients physically, psychologically and financially [58-60], and formal psychological support may be beneficial [61, 62]. Several of those considering SCT valued one-to-one support, as also noted by Tariman [20]. Furthermore, it has been suggested that information about 'personal experiences' can complement 'general facts', and contribute to decision support in various ways, for example, by helping people clarify their own values and reasoning, either by suggesting different ways of thinking and/or by providing a 'sounding board' against which to test their own ideas [63].

Strengths and Limitations

As far as we are aware, this is the first UK study to specifically explore involvement in TDM in patients with haematological malignancies. Our sample size of 35 enabled in-depth exploration of the research questions, and use of semi-structured interviews allowed participants to focus on issues they themselves considered significant. Our sampling framework ensured 'key informants' were interviewed from targeted disease subtypes, both sexes and various age-groups. As the diseases included are typically relapsing-remitting conditions, we felt it important to include patients (and relatives) whose perceptions may have altered over time, during prolonged W&W or following treatment, to capture as broad a range of views as possible. To counteract the influence of memory, we also invited some recently diagnosed patients to take part; reference to patient diaries and contributions from relatives also enhanced recall.

For peer review only - http://bmjopen.bmj.com/site/about/guidelines.xhtml

Attempts to recruit patients from black and minority ethnic (BAME) backgrounds were unfortunately unsuccessful. As Morse [64] has highlighted how merging data from a small number of BAME participants can result in loss of cultural differences when analysed alongside the remainder, who share a single identity, we recommend future in-depth studies, dedicated to those whose heritage differs from participants in our own study. Furthermore, fewer people living in more deprived areas agreed to take part, compared to those in affluent areas. Consequently, a further limitation was our inability to recruit patients with low levels of literacy, who may have been deterred because the study invitation and information was provided in writing. We therefore recognise that the views of our participants are unlikely to reflect those of the entire population with the diseases of interest. Nonetheless, it is highly likely that a large proportion of our findings are transferable to other UK areas, and also countries with similar health-care infrastructure and universal health-care coverage.

Clinical Implications

Our findings suggest that our interviewees varied in their preference for involvement in TDM according to intrinsic, contextual, and disease-related factors, requiring clinicians to assess individuals' preferences for engagement at multiple time points. Fisher et al. [41] comment that clinicians who clarify patients' preferences and ensure they are informed about their options, are sharing the deliberation aspect of decision making, even if the doctor ultimately provides a strong recommendation. Entrusting clinical staff to make recommendations does not appear to diminish patients' desire for discussion of possible options, and for provision of relevant information that matches their individual needs. Empathetic relationships with clinicians seem highly valued by patients, and appear conducive to engagement in TDM. Deliberation of treatment options can be highly distressing for some patients, and those lacking support from family members/others may benefit from formal assessment and referral for psychological support.

CONCLUSION

This study revealed that patients with haematological cancers may wish to be involved in TDM to varying extents, contingent on complex, inter-related factors, that are dynamic and subject to change according to clinical and personal contexts. Overall, our interviewees expressed a strong preference for acceptance of clinician recommendations, linked to disease complexity, patients' trust in clinician expertise, and perceptions of trusted patient-clinician relationships.

For peer review only

For peer review only - http://bmjopen.bmj.com/site/about/guidelines.xhtml

Abbreviations and Declarations

Abbreviations

TDM: Treatment decision making; NHS: National Health Service; UK: United Kingdom; P: Patient; R: Relative.

Declarations

Ethics approval and consent to participate

Research projects set within HMRN's infrastructure and collecting supplementary data require ethical approval, which was granted by the London, City and East committee (REC:16/ LO/0740). Assurances of confidentiality and anonymity were given to participants and written consent was obtained from patients and relatives.

Consent for publication

All interviewees consented to the use of quotations from their interviews in publications arising from the study.

Availability of data and materials

All data and materials relating to this research are from the Haematological Malignancy Research Network and are archived and maintained by the first and last author, according to organisational and ethical regulations. Data are not publicly available due to the risk of participant identification from specific contexts revealed when reading entire transcripts and due to the terms and conditions regarding the release of data to third parties upon which ethical approvals for this study were contingent. Reasonable requests for further information relating to this data can be made to the corresponding author.

Competing interests

The authors declare that they have no competing interests.

Funding

This work was supported by the NIHR via a PGfAR: RP-PG-0613-2002, Cancer Research UK: 29685, and Blood Cancer UK (formerly Bloodwise): 15037. None of the funding bodies were involved in the design of the study, nor in the collection, analysis, interpretation and reporting of data; the views expressed here are those of the authors and do not necessarily reflect those of the funder.

Authors' contributions

DH, ER, AS and RP designed the study. AS identified potential participants and mapped pathways. DH and DM recruited the study participants, and DM conducted interviews. Transcripts were coded and analysed by DM with discussion/input from DH. DM wrote the first draft of the manuscript. DH, ER, AS revised the manuscript. RP commented on the clinical aspects of the study. All authors read and approved the final version.

Acknowledgments

We wish to thank the study participants who took part in an interview and shared sensitive and emotive issues.

For peer review only

For peer review only - http://bmjopen.bmj.com/site/about/guidelines.xhtml

REFERENCES:

1. Office for National Statistics. Cancer Registration Statistics, England: 2017. https://www.ons.gov.uk/peoplepopulationandcommunity/healthandsocialcare/conditionsanddiseases/bulletins/cancerregistrationstatisticsengland/2017 (release date April, 2019) Accessed 15 Jan 2021.

2. Public Health England National Statistics Cancer Registration Statistics, England: final release, 2018. Gov.UK (Published 29 May, 2020). https://www.gov.uk/government/publications/cancer-registration-statistics-england-2018-final-release/cancer-registration-statistics-england-final-release-2018. Accessed 15 Jan 2021.

3. Blood Cancer UK, 2018. https://bloodcancer.org.uk/news/latest-statistics Accessed 15 Jan 2021.

4. National Institute for Clinical Excellence (NICE) Interactive Flowcharts. 2020. https://pathways.nice.org.uk/pathways/ Accessed 15 Jan 2021.

5. British Society for Haematology. Guidelines. 2020. https://b-s-h.org.uk/guidelines/ Accessed 15 Jan 2021.

6. Smith A, Howell D, Patmore R, Jack A, Roman E. Incidence of haematological malignancy by sub-type: a report from the Haematological Malignancy Research Network. Br J Cancer. 2011;105:1684–92. Available from: https://doi.org/10.1038/bjc.2011.450.

7. Smith A, Howell D, Crouch S, Painter D, Blase J, Wang H-I, et al. Cohort Profile: The Haematological Malignancy Research Network (HMRN): a UK population-based patient cohort. Int J Epidemiol. 2018; 7:700-700g. Available from: https://doi.org/10.1093/ije/dyy044.

8. Im A, Pavletic SZ. Immunotherapy in hematologic malignancies: past, present, and future. J Hematol Oncol 2017;10:94. Available from: https://doi.org/10.1186/s13045-017-0453-8.

9. Gauthier J, Yakoub-Agha I. Chimeric antigen-receptor T-cell therapy for hematological malignancies and solid tumors: Clinical data to date, current limitations and perspectives. Curr Res Transl Med.2017;65:93-102. Available from: https://doi.org/10.1016/j.retram.2017.08.03.

10. Institute of Medicine (US) Committee on Quality of Health Care in America. Crossing the Quality Chasm: a new Health System for the 21st Century. Washington (DC): Natl Acad Press (US); 2001. Available from: https://doi.org/10.17226/10027.

For peer review only - http://bmjopen.bmj.com/site/about/guidelines.xhtml

11. Coulter A, Collins A. Making Shared Decision Making a Reality: No decision about me, without me. Report from The King's Fund. 2011; London: The King's Fund. Available from: https://www.kingsfund.org.uk/sites/default/files/Making-shared-decision-making-a-reality-paper-Angela-Coulter-Alf-Collins-July-2011_0.pdf. Accessed 15 Jan 2021.

12. Barry M, Edgman-Levitan S. Shared Decision Making – the Pinnacle of Patient-Centred Care. New Engl J Med. 2012;366(9):780-1. Available from: https://doi.org/10.1056/NEJMp1109283.

13. Elwyn G, Frosch D, Thomson R, Joseph-Williams N, Lloyd A, Kinnersley P, Cording E, Tomson D, Dodd C, Rollnick S, Edwards A, Barry M. Shared Decision Making: A Model for Clinical Practice. J Gen Intern Med. 2012;27(10):1361–7 Available from: https://doi.org/10.1007/s11606-012-2077-6.

14. Elwyn G, Durand MA, Song J, Aarts J, Barr PJ, Berger Z, et al. A three-talk model for shared decision making: multistage consultation process. BMJ. 2017;359:j4891 Available from: http://dx.doi.org/10.1136/bmj.j4891

15. LeBlanc TW, Baile WF, Eggly S, Bylund CL, Kurtin S, Khurana M, et al. Review of the patient-centered communication landscape in multiple myeloma and other hematologic malignancies. Pat Educ Couns. 2019;102:1602-1612. Available from: https://doi.org/10.1016/j.pec.2019.04.028.

16. Mulley AG, Trimble C, Elwyn G. Stop the silent misdiagnosis: patients' preferences matter. BMJ. 2012;345:e6572. Available from: https://doi.org/10.1136/bmj.e6572.

17. Elwyn G, Cochran N, Pignone M. Shared Decision Making – the Importance of Diagnosing Preferences. JAMA Intern Med. 2017;177(9):1239-1240. Available from: https://doi.org/10.1001/jamainternmed.2017.1923.

18. Bomhof-Roordink H, Fischer MJ, van Duijn-Bakker N, Baas-Thijssen MC, van der Weijden T, Stiggelbout AM, Pietersei AH. Shared decision making in oncology: A model based on patients', healthcare professionals' and researchers' views. Psycho-Oncology. 2019;28 (1):139–146. Available from: https://doi.org/10.1002/pon.4923.

19. Alexander SC, Sullivan AM, Back AL, Tulsky JA, Goldman RE, Block SD, et al. Information giving and receiving in haematology malignancy consultations. Psycho-Oncology. 2012;21(3):297–306. Available from: https://doi.org/10.1002/pon.1891.

20. Tariman JD, Doorenbos A, Schepp KG, Becker PS, Berry DL. Patient, Physician and Contextual Factors Are Influential in the Treatment Decision Making of Older Adults Newly Diagnosed with

For peer review only - http://bmjopen.bmj.com/site/about/guidelines.xhtml

Symptomatic Myeloma. Cancer Treat Commun. 2014;2:34-47. Available from: https://doi.org/10.1016/j.ctrc.2014.08.003.

21. Rood JAJ, van Zuuren, FJ, Stam F, van der Ploeg T, Eeltink C, Verdonck-de Leeuws IM, Huigens PC. Perceived need for information among patients with a haematological malignancy: associations with information satisfaction and treatment decision-making preferences. Hematol Oncol. 2015;33 (2): 85–98. Available from: https://doi.org.10.1002/hon.2138.

22. Rood JAJ, Nauta IH, Witte BI, Stam F, van Zuuren FJ, Manenschijn A, et al. Shared decision-making and providing information among newly diagnosed patients with haematological malignancies and their informal care-givers: Not “one size fits all”. Psycho-Oncology. 2017;26(12):2040–2047. Available from: https://doi.org/10.1002/pon.4414.

23. Tong A, Sainsbury P, Craig J. Consolidated criteria for reporting qualitative research (COREQ): a 32-item checklist for interviews and focus groups. Int J Qual Health Care. 2007; 19(6):349-57. Available from: https://doi.org/10.1093/intqhc/mzm042.

24. Sandelowski M. 2000. Focus on research methods: Whatever happened to qualitative description? Res Nurs Health. 23(4):334-340. Available from: https://doi.org/10.1002/1098-240x(200008)23:4<334::aid-nur9>3.0.co;2-g.

25. Neergaard MA, Olesen F, Andersen RS, Sondergaard J. Qualitative description-the poor cousin of health research? BMC Med Res Methodol. 2009;9:52. Available from: https://doi.org/10.1186/1471-2288-9-52.

26. Gentles SJ, Charles C, Ploeg J, McKibbon KA. Sampling in Qualitative Research: Insights from an Overview of the Methods Literature. The Qualitative Report. 2015; 20:1772-1789. Available from: http://nsuworks.nova.edu/tqr/vol20/iss11/5

27. Malterud K, Siersma VD, Guassora AD. Sample size in qualitative interview studies: Guided by information power. Qual Health Res. 2016; 26(13):1753–1760. Available from: https://doi.org/10.1177/1049732315617444.

28. Saunders B, Sim J, Kingstone T, Baker S, Waterfield J, Bartlam B, et al. Saturation in qualitative research: Exploring its conceptualization and operationalization. Qual Quant. 2018; 52(4):1893–1907. Available from: https://doi.org/10.1007/s11135-017-0574-8.

For peer review only - http://bmjopen.bmj.com/site/about/guidelines.xhtml

29. Thorne, S. The Great Saturation Debate: What the "S Word" Means and Doesn't Mean in Qualitative Research Reporting. Can J Nurs Res. 2020; 52: 3–5. Available from: https://doi.org/10.1177/0844562119898554.

30. Morse J. Confusing categories and themes. (Editorial). Qual Health Res. 2008;18 (6):727-728. Available from: https://doi.org/10.1177/1049732308314930.

31. Bradshaw C, Atkinson S, Doody O. Employing a Qualitative Description Approach in Health Care Research. Glob Qual Nurs Res. 2017;24 (4):2333393617742282. Available from: https://doi.org/10.1177/2333393617742282.

32. Caelli K, Ray L, Mill, J. "Clear as mud": Toward greater clarity in generic qualitative research. Int J Qual Methods. 2003;2:1–23. Available from: https://doi.org/10.1177/160940690300200201.

33. Pope C, Mays N. (eds). Qualitative Research in Health Care. 3rd ed. Oxford: BMJ Blackwell Publishing; 2006.

34. Sandelowski, M. (2010). What's in a name? Qualitative description revisited. Res Nurs Health. 33(1):77–84. Available from: https://doi.org/10.1002/nur.20362.

35. Green J, Thorogood N. Qualitative Methods for Health Research: Introducing Qualitative Methods Series. 4th ed. London: Sage; 2018, p258.

36. Pope C, Ziebland S, Mays N. Analysing qualitative data. BMJ. 2000;8;320 (7227): 114–116. Available from: https://doi.org/10.1136/bmj.320.7227.114.

37. Barbour RS. 2001. Checklists for improving rigour in qualitative research: a case of the tail wagging the dog? BMJ. 2001;322:1115–1117. Available from: https://doi.org/10.1136/bmj.322.7294.1115.

38. Waldron T, Carr T, McMullen L, Westorp G, Duncan V, Neufeld S-M, Bandura L-A, Groot G. Development of a Programme Theory for Shared Decision Making: a realist synthesis. BMC Health Serv Res. 2020;20(1):59. Available from: https://doi.org/10.1186/s12913-019-4649-1.

39. Entwistle VA, Watt IS. Patient involvement in treatment decision making: the case for a broader conceptual framework. Pat Ed Couns. 2006;63(3):268-278. Available from: https://doi.org/10.1016/j.pec.2006.05.002.

40. Joseph-Williams N, Elwyn G, Edwards, A. Knowledge is *not* power for patients: A systematic review and thematic synthesis of patient-reported barriers and facilitators to shared decision

For peer review only - http://bmjopen.bmj.com/site/about/guidelines.xhtml

making. Pat Educ Couns. 2014.94(3):291-309. Available from: https://doi.org/10.1016/j.pec.2013.10.031.

41. Fisher KA, Tan ASL, Matlock DD, Saver B, Mazor KM, Pieterse AH. Keeping the patient in the center: Common challenges in the practice of shared decision making. Pat Educ Couns. 2018;101(12):2195–2201. Available from: https://doi.org/10.1016/j.pec.2018.08.007.

42. Pollard S, Bansback N, Bryan S. Physician attitudes toward shared decision making: A systematic Review. Pat Educ Couns. 2015;98(9):1046-1057. Available from: https://doi.org/j.pec.2015.05.004.

43. Zeuner R, Frosch DL, Kuzemchak MD, Politi MC. Physicians' perceptions of shared decision-making behaviours: a qualitative study demonstrating the continued chasm between aspirations and clinical practice. Health Expect. 2014;18(6):2465–2476. Available from: https://doi.org/10.1111/hex.12216.

44. Rocque GB, Williams CP, Halilova KI, Borate U, Jackson BE, Van Laar ES, et al. Improving shared decision making in chronic lymphocytic leukaemia through multidisciplinary education. Trans Behav Med. 2018;8:175-182. Available from: https://doi.org/10.1093/tbm/ibx034.

45. Blumenthal-Barby JS. 'That's the doctor's job': Overcoming patient reluctance to be involved in medical decision making. Pat Educ Couns. 2017; 100(1):14-17. Available from: https://doi.org/10.1016/j.pec.2016.07.010.

46. National Institute for Clinical Excellence (NICE). Haematological cancers: improving outcomes NICE guideline [NG47]. https://www.nice.org.uk/guidance/ng47/evidence/improving-outcomes-in-haematological-cancers-the-manual-pdf-2487893581. Accessed 15 Jan 2021.

47. McCaughan D, Roman E, Smith AG, Garry A, Johnson M, Patmore R, et al. Determinants of hospital death in haematological cancers: findings from a qualitative study. BMJ Supp Palliat Care. 2018;8(1):78–86. Available from: https://doi.org/10.1136/bmjspcare-2016-001289.

48. Hahlweg P, Harter M, Nestoriuc Y, Scholl I. How are decisions made in cancer care? A qualitative study using participant observation of current practice. BMJ Open. 2017;7(9):e016360. Available from: https://doi.org/10.1136/bmjopen-2017-016360.

49. Lowe MM, Osther PJS, Ammentorp J, Birkelund R. The Balance of Patient Involvement: Patients' and Health Professionals' Perspectives on Decision-Making in the Treatment of Advanced Prostate Cancer. Qual Health Res. 2021;31(1):29-40. Available from: https://doi.org/10.1177/1049732320962759.

For peer review only - http://bmjopen.bmj.com/site/about/guidelines.xhtml

50. Coulter A. Patient information and shared decision-making in cancer care. Br J Cancer. 2003;89 Suppl 1:S15-16. Available from: https://doi.org/10.1038/sj.bjc.6601080.

51. Legare F, Stacey D, and IP TEAM. IP-SDM model. 2010. https://decisionaid.ohri.ca/docs/develop/IP-SDM-Model.pdf Accessed 15 Jan 2021.

52. Atherton K, Young B, Salmon P. Understanding the information needs of people with haematological cancers. A meta-ethnography of quantitative and qualitative research. Eur J Cancer Care. 2017;26(6):e12647. Available from: https://doi.org/10.1111/ecc.12647.

53. Loh KP, Tsang M, LeBlanc TW, Back A, Duberstein PR, Mohile SG, et al., Decisional involvement and information preferences of patients with hematologic malignancies. Blood Adv. 2020; 4(21):5492-5500. Available from: https://doi.org/10.1182/bloodadvances.2020003044.

54. Atherton K, Young B, Kalakonda N, Salmon P. Perspectives of patients with haematological cancer on how clinicians meet their information needs: “Managing” information versus “giving” it. Psycho-Oncology. 2018;27(7):1719–1726. Available from: https://doi.org/10.1002/pon.4714.

55. Chhabra KR, Pollack KI, Lee SJ, Back AL, Goldman RE, Tulsky JA. Physician Communication Styles in Initial Consultations for Hematological Cancer. Pat Educ Couns. 2013; 93(3):573-8. Available from: https://doi.org/10.1016/j.pec.2013.08.023.

56. LeBlanc TW, Fish LJ, Bloom CT, El-Jawahri A, Davis DM, Locke SC, et al. Patient experiences of acute myeloid leukemia: A qualitative study about diagnosis, illness understanding, and treatment decision-making. Psycho-Oncology. 2017;26(12):2063–2068. Available from: https://doi.org/10.1002/pon.4309.

57. Laidsaar-Powell RC, Butow PN, Bu S, Charles C, Gafni A, Lam WWT, et al. Physician–patient–companion communication and decision-making: a systematic review of triadic medical consultations. Patient Educ Couns. 2013; 91(1):3–13. Available from: https://doi.org/10.1016/j.pec.2012.11.007.

58. Mosher CE, DuHamel KN, Rini C, Corner G, Lam J, Redd WH. Quality of Life Concerns and Depression among Hematopoietic Stem Cell Transplant Survivors. Support Care Cancer. 2011; 19(9): 1357–1365. Available from: https://doi.org.10.1007/s00520-010-0958-y.

For peer review only - http://bmjopen.bmj.com/site/about/guidelines.xhtml

59. Walpole G, Clark H, Dowling M. Myeloma patients' experiences of haematopoietic stem cell transplant: A qualitative thematic synthesis. Eur J Onc Nurs. 2018;35:15-21. Available from: https://doi.org/10.1016/j.ejon.2018.05.002.

60. Ruark J, Mullane E, Cleary N, Cordeiro A, Bezzero ED, Wu V, et al., Patient-Reported Neuropsychiatric Outcomes of Long-Term Survivors after Chimeric Antigen Receptor T Cell Therapy. Biol Blood Marrow Transplant. 2020; 26(1):34-43. Available from: https://doi.org/10.1016j.bbmt.2019.09.037.

61. Swash B, Hulbert-Williams N, Bramwell R. Unmet psychosocial needs in haematological cancer: a systematic review. Support Care Cancer. 2014; 22(4):1131-1141. Available from: https://doi.org/10.1007/s00520-014-2123-5.

62. Oberoi DV, White VM, Seymour JF, Prince HM, Harrison S, Jefford M et al. Distress and unmet needs during treatment and quality of life in early cancer survivorship: A longitudinal study of haematological cancer patients. Eur J Haematol. 2017; 99(5):423-430. Available from: https://doi.org/10.1111/ejh.12941.

63. Entwistle VA, France EF, Wyke S, Jepson R, Hunt K, Ziebland S, Thompson A. How information about other people's personal experiences can help with healthcare decision-making: a qualitative study. Patient Educ Couns. 2011;85(3):e291–8. Available from: https://doi.org/10.1016/j.pec.2011.05.014.

64. Morse J. The Changing Face of Qualitative Inquiry. Invited IIQM Conference Keynote address 2019. Int J Qual Methods. 2020; 19:1–7. Available from: https://doi.org/10.1177/1609406920909938.

65. Kim H, Sefcik JS, Bradway C. Characteristics of qualitative descriptive studies: A systematic review. Res Nurs Health. 2017;40(1):23–42. Available from: https://doi.org/10.1002/nur.21768.

For peer review only - http://bmjopen.bmj.com/site/about/guidelines.xhtml

Figure 1. Factors implicated in patient involvement in treatment decisions

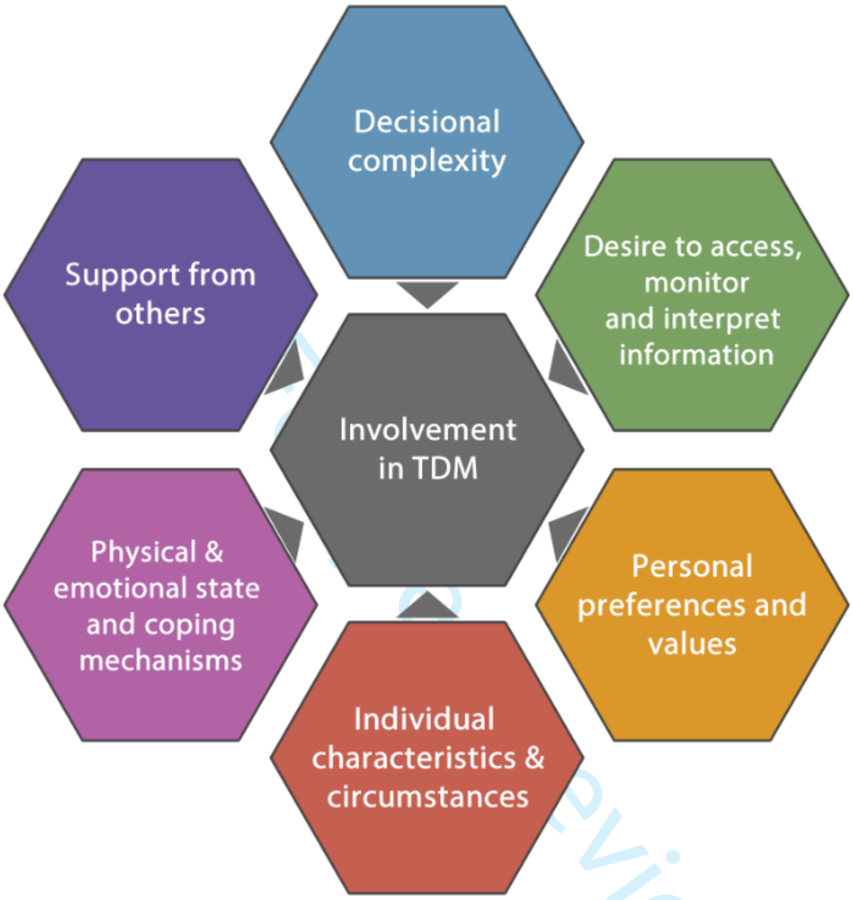

Figure 2. Continuum of characteristics associated with proactive and non-proactive approaches to involvement in treatment decisions

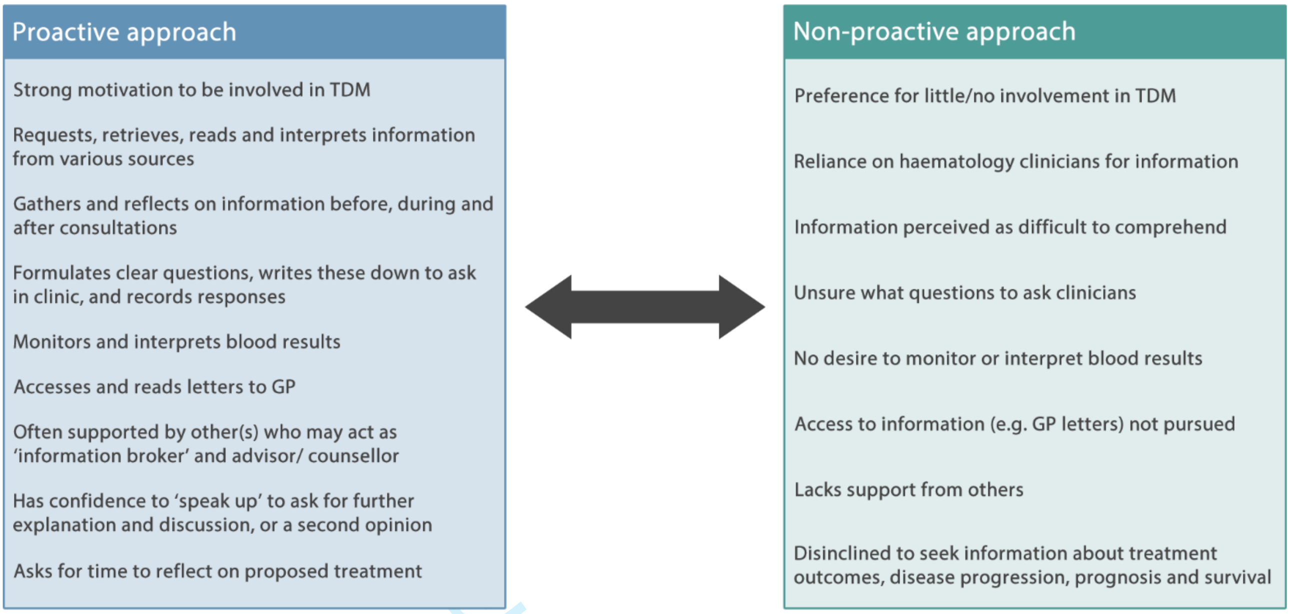

r review only

Supplementary file 1: Topic guide for interviews with patients and relatives

Focusing on information and decisions at key states (diagnosis, W&W, treatment) and progression through states.

Information

- How important is it to you that you receive information about your cancer? *(why is that?*)
- How do you feel about the information given to you at diagnosis/start of treatment?
- How do you feel about getting information from HCPs more generally? *(time constraints; overwhelming; difficult to understand/take in; use of language/terminology)*
- Do you feel the information given applies specifically to you? *(personalized, tailored, specific)*
- How healthcare practitioners (HCPs) ascertain your information needs?
- Is the information you received explained in a way you can understand? *(technical language; too detailed; not detailed enough)*
- What do HCPs do to check if you understand the information they give you?
- How do you feel about asking questions? Are your questions always answered?
- Do you feel that your information needs are usually met? What worked well and could have been better? *(diagnosis; treatment initiation/cessation - examples)*
- What do you think about the timing of information from HCPs? When is the right time? *(at diagnosis; during clinic appointments; when disease status changes; at other times)*
- How do/did you feel about discussing the risks/benefits of different treatments with HCPs?
- How do you feel about discussing prognosis? *("a statement about expectations that refers to the likely course of the cancer and/or outcome") (want to know/not; timing; language)*
- What strategies do you use to absorb information? (*in general, how bad news is processed*)

Treatment decisions

- How do you feel about being involved in decisions with HCPs about your treatment?
- Have you been asked you if you want to be involved in decisions about treatment?
- Do you want to be involved in decisions? *(preference for patient only; clinician only; patient/clinician)*
- What should be considered during treatment decision making? *(effectiveness of treatment; side effects; prognosis; patient goals, values, preferences; impact on quality of life)*
- What might make it easier or harder for you to be involved in making decisions about your treatment? *(time; style of communication; how information is conveyed; explanations)*
- Are there particular time-points when it is harder to be involved in making decisions about treatment? *(diagnosis; treatment initiation/change; treatment cessation)*

Practical issues

- How do you feel about the amount of information you get? *(prefer more/less; overloaded; struggle to absorb)*
- What do you want to know/know more about? *(investigations; treatments; prognosis; side effects; QoL)*
- Where/who do you prefer to get information; why? *(Internet; doctors/nurses; family; leaflets; support group)*
- What do you think about different sources of information? *(credibility, ability to judge)*
- How do you prefer to see information about risks and benefits; why? (w*ords/numbers; figures/percentages; diagrams/graphs)*

For peer review only

COREQ (COnsolidated criteria for REporting Qualitative research) Checklist

Topic	No.	Guide Questions/Description	Page	Explanation
**Domain 1: Research team and reflexivity**				
**Personal characteristics**				
Interviewer	1	Which author conducted the interview?	p5	Dorothy McCaughan (DM)
Credentials	2	What were the researcher's credentials? E.g. PhD, MD	n/a	BA, RN MSc
Occupation	3	What was their occupation at the time of the study?	n/a	Senior Research Fellow
Gender	4	Was the researcher male or female?	n/a	Female
Experience and training	5	What experience or training did the researcher have?	n/a	Registered Nurse. Conducted many qualitative interviews patients.
**Relationship with participants**				
Relationship established	6	Was a relationship established prior to the study?	n/a	No relationship existed with participants prior to the study.
Participant knowledge of the interviewer	7	What did participants know about the researcher? e.g. personal goals, reasons for doing the research?	n/a	Participants had no knowledge of the interviewer prior to the study.
Interviewer characteristics	8	What characteristics were reported about the interviewer? e.g. Bias, assumptions, interests in topic	p5	Data were collected by DM, analysed by DM & DH, both qualified nurses and experienced health services researchers.
**Domain 2: Study design**				
**Theoretical framework**				
Methodological orientation and theory	9	What methodological orientation underpinned the study? e.g. grounded theory, content analysis	p5	Qualitative description and thematic content analysis (referenced in the Methods).
**Participant selection**				
Sampling	10	How were participants selected? e.g. purposive, convenience, consecutive,	p4	Interviewees were purposively selected.
Method of approach	11	How were participants approached?	p5	Interviewees were recruited by mail. Potential participants contacted DM if they wanted to take part.
Sample size	12	How many participants were in the study?	p5	Thirty-five patient interviews were undertaken, ten included relatives.
Non-participation	13	How many people refused to participate or dropped out? Reasons?	n/a	After invitation, around 20 patients did not contact the study team; reasons for this are unknown. No-one who responded dropped out.
Setting of data collection	14	Where was the data collected? e.g. home, clinic, workplace	p5	Data were largely collected in patient homes. A small number took place in the University or elsewhere.
Presence of nonparticipants	15	Was anyone else present besides the participants and researchers?	p5	Interviews were conducted privately.

For peer review only

Description of sample	16	What are the important characteristics of the sample? e.g. demographic data, date	p4<br>Table 1	The sample was selected from HMRN and included patients with chronic haematological malignancies, who had agreed they could be contacted for research (men and women of any ethnicity and age, living in any area).
Interview guide	17	Were questions, prompts, guides provided by the authors? Was it pilot tested?	p5<br>Appx. 1	A semi-structured topic guide, tested on 2 patients, was developed from the literature and team experiences.
Repeat interviews	18	Were repeat interviews carried out? If yes, how many?	n/a	No, there were no repeat interviews.
Audio/visual recording	19	Was audio or visual recording used to collect the data?	p5	Audio recording was used.
Field notes	20	Were field notes made during and/or after the interview?	n/a	Field notes were made post-interview.
Duration	21	What was the duration of the interviews?	p5	Interviews lasted 40-90 minutes.
Data saturation	22	Was data saturation discussed?	p5	Interviews continued until saturation appeared to have been reached.
Transcripts returned	23	Were transcripts returned to participants for comment and/or correction?	n/a	This was not requested and audiotaping was clear.
**Domain 3: analysis and findings**				
**Data analysis**				
Number of data coders	24	How many data coders coded the data?	p5	DM & DH were involved in coding and discussed/developed the scheme.
Description of the coding tree	25	Did authors provide a description of the coding tree?	p5-6<br>Fig. 1	The coding scheme (tree) reflected the topic guide and was developed to accommodate data content.
Derivation of themes	26	Were themes identified in advance or derived from the data?	p5	Themes were derived from the data. The topic guide provided a framework for data collection.
Software	27	What software, if applicable, was used to manage the data?	n/a	Data were managed in Word and Excel.
Participant checking	28	Did participants provide feedback on the findings?	n/a	Feedback was provided to patients via the study newsletter.
**Reporting**				
Quotations presented	29	-Were quotations presented to illustrate themes/findings?<br>-Were quotations linked to ID?	p7-16	Quotations are presented with anonymised IDs, linked to more detailed characteristics in Table 1.
Data and findings consistent	30	Was there consistency between data and findings?	p7-16	We consistently link findings with supporting quotations.
Clarity of major themes	31	Were major themes clearly presented in the findings?	p7-16<br>Fig. 1&2	Five major themes are clearly defined, with separate headings.
Clarity of minor themes	32	Is there a description of diverse cases or discussion of minor themes?	p5, p19	Whilst highlighting the main themes, we also incorporated a range of responses and drew attention to negative cases.

Developed from: Tong A, Sainsbury P, Craig J. Consolidated criteria for reporting qualitative research (COREQ): a 32-item checklist for interviews and focus groups. International Journal for Quality in Health Care. 2007. Volume 19, Number 6: pp. 349 – 357.

For peer review only

BMJ Open

Treatment decision making (TDM): A qualitative study exploring the perspectives of patients with chronic haematological cancers

Journal:	*BMJ Open*
Manuscript ID	bmjopen-2021-050816.R1
Article Type:	Original research
Date Submitted by the Author:	04-Oct-2021
Complete List of Authors:	McCaughan, Dorothy; University of York, Health Sciences<br>Roman, Eve; University of York, Health Sciences<br>Smith, Alexandra; University of York, Health Sciences<br>Patmore, Russell; Castle Hill Hospital, Queens Centre for Oncology<br>Howell, Debra; University of York, Health Sciences
**Primary Subject Heading**:	Haematology (incl blood transfusion)
Secondary Subject Heading:	Health services research, Patient-centred medicine, Qualitative research
Keywords:	Myeloma < HAEMATOLOGY, Leukaemia < HAEMATOLOGY, Lymphoma < HAEMATOLOGY, QUALITATIVE RESEARCH, Organisation of health services < HEALTH SERVICES ADMINISTRATION & MANAGEMENT, CHEMOTHERAPY

SCHOLARONE™
Manuscripts

I, the Submitting Author has the right to grant and does grant on behalf of all authors of the Work (as defined in the below author licence), an exclusive licence and/or a non-exclusive licence for contributions from authors who are: i) UK Crown employees; ii) where BMJ has agreed a CC-BY licence shall apply, and/or iii) in accordance with the terms applicable for US Federal Government officers or employees acting as part of their official duties; on a worldwide, perpetual, irrevocable, royalty-free basis to BMJ Publishing Group Ltd ("BMJ") its licensees and where the relevant Journal is co-owned by BMJ to the co-owners of the Journal, to publish the Work in this journal and any other BMJ products and to exploit all rights, as set out in our licence.

The Submitting Author accepts and understands that any supply made under these terms is made by BMJ to the Submitting Author unless you are acting as an employee on behalf of your employer or a postgraduate student of an affiliated institution which is paying any applicable article publishing charge ("APC") for Open Access articles. Where the Submitting Author wishes to make the Work available on an Open Access basis (and intends to pay the relevant APC), the terms of reuse of such Open Access shall be governed by a Creative Commons licence – details of these licences and which Creative Commons licence will apply to this Work are set out in our licence referred to above.

Other than as permitted in any relevant BMJ Author's Self Archiving Policies, I confirm this Work has not been accepted for publication elsewhere, is not being considered for publication elsewhere and does not duplicate material already published. I confirm all authors consent to publication of this Work and authorise the granting of this licence.

For peer review only

TITLE: Treatment decision making (TDM): A qualitative study exploring the perspectives of patients with chronic haematological cancers

AUTHORS: Dorothy McCaughan, Eve Roman, Alexandra Smith, Russell Patmore, Debra Howell

CORRESPONDING AUTHOR:

Debra Howell
Epidemiology & Cancer Statistics Group, Department of Health Sciences, Seebohm Rowntree Building, University of York, York, UK. YO10 5DD.
Telephone: +44 (0)904 321895
Email: debra.howell@york.ac.uk

AUTHORS
Dorothy McCaughan
Epidemiology & Cancer Statistics Group, Department of Health Sciences, Seebohm Rowntree Building, University of York, York, UK. YO10 5DD.
Telephone: +44 (0) 904 321348
Email: dorothy.mccaughan@york.ac.uk

Eve Roman
Epidemiology & Cancer Statistics Group, Department of Health Sciences, Seebohm Rowntree Building, University of York, York, UK. YO10 5DD.
Telephone: +44 (0) 904 321882
Email: eve.roman@york.ac.uk

Alexandra Smith
Epidemiology & Cancer Statistics Group, Department of Health Sciences, Seebohm Rowntree Building, University of York, York, UK. YO10 5DD.
Telephone: + 44 (0) 904 321884

Russell Patmore
Queens Centre for Oncology and Haematology, Castle Hill Hospital, Cottingham, UK.
Telephone: +44 (0) 482 604444
Email: Russell.Patmore@hey.nhs.uk

Debra Howell
Epidemiology & Cancer Statistics Group, Department of Health Sciences, Seebohm Rowntree Building, University of York, York, UK. YO10 5DD
Telephone: +44 (0)904 321895
Email: debra.howell@york.ac.uk

KEYWORDS: leukaemia, lymphoma, myeloma, treatment decision making, qualitative research

WORD COUNT: 6212 including quotations

For peer review only - http://bmjopen.bmj.com/site/about/guidelines.xhtml

ABSTRACT

Objectives

Haematological malignancies are the fifth most common cancer in the UK, with chronic subtypes comprising around a third of all new diagnoses. These complex diseases have some similarities with other cancers, but often require different management. Surgical resection is not possible, and while some are curable with intensive chemotherapy, most indolent subtypes are managed with non-aggressive intermittent or continuous treatment, often over many years. Little is known about the views of patients with chronic haematological cancers regarding treatment decision making (TDM), a deficit our study aimed to address.

Setting and design

Set within the Haematological Malignancy Research Network (HMRN: www.hmrn.org), an ongoing population-based cohort that provides infrastructure to support evidence-based research, HMRN data were augmented by qualitative information from in-depth interviews. Data were analysed for thematic content, combining inductive and deductive approaches. Interpretation involved seeking meaning, salience and connections within data.

Participants

Thirty-five patients with four chronic subtypes: chronic lymphocytic leukaemia, follicular lymphoma, marginal zone lymphoma, and myeloma. Ten relatives were present and contributed to varying extents.

Results

Five themes were discerned: 1. Preference for clinician recommendations; 2. Factors implicated in patient involvement in TDM; 3. Perceptions of proactive/non-proactive approaches to TDM; 4. Experiences of TDM at various points in the disease trajectory; 5. Support from others. Our principal finding relates to a strong preference among interviewees for treatment recommendations from haematologists, based on trust in their expertise and perceptions of empathetic patient-clinician relationships.

Conclusion

Interviewees wanted to be involved in TDM to varying extents, contingent on complex, inter-related factors, that are dynamic and subject to change according to differing clinical and personal contexts. Patients may benefit from clinicians assessing their shifting preferences for involvement on multiple occasions. Strong preferences for acceptance of recommendations was associated with cancer complexity, trust in clinician expertise, and positive perceptions of patient-clinician relationships.

For peer review only - http://bmjopen.bmj.com/site/about/guidelines.xhtml

Strengths and limitations

- Set within the infrastructure of an established population-based study, this is (to our knowledge) the first UK study to specifically explore TDM involvement in patients with chronic haematological malignancies.
- Interview data supplemented robust clinical information routinely collected as part of the broader cohort.
- The sample size (35 patients) enabled in-depth exploration of the research questions, enhanced by contributions from relatives/carers.
- Purposive sampling ensured inclusion of individuals with differing demographic and disease profiles, diagnosed at different time points on the clinical pathway.
- The views of participants are unlikely to reflect those of the entire patient population, and dedicated studies of people from black and minority ethnic backgrounds and those with low literacy levels are required.

For peer review only

TITLE: Treatment decision making: A qualitative study exploring the perspectives of patients with chronic haematological cancers

BACKGROUND

Haematological malignancies are the fifth most common cancer in the UK [1-3]. They are complex diseases, and although they have some similarities with other cancers, they often require different management. Surgery is not an option, and while some subtypes are curable with intensive chemotherapy and periods of hospitalisation, other more chronic subtypes are not. These more indolent malignancies, which account for around 30% of all newly diagnosed haematological cancers [4, 5], can, however, often be controlled for long periods; typically (but not always) following remitting/relapsing pathways wherein periods of chemotherapy are interspersed with active monitoring, known as Watch and Wait (W&W) [6, 7]. These pathways are associated with variations in the extent of hospital activity, with periods of disease progression, relapse and treatment requiring more consultations and decisions, and W&W having fewer contact points, often months apart. Haemato-oncology is a rapidly changing field, and the past decade has seen the development of innovative treatment regimens that include targeted therapies, along with new and established chemotherapies, radiotherapy, and haematopoietic stem cell transplantation (SCT) [8]. While obviously beneficial, these developments increase treatment decisional complexity for clinicians and patients [9].

Shared decision making (SDM), seen as a hallmark of quality care, is part of a broader concept of patient-centred care which considers individual preferences, needs, and values, with the aim of ensuring patient values guide all clinical decisions [10-12]. Steps integral to SDM include the clinician informing the patient of treatment options and the need for a decision; discussion between patient and clinician of each option; and the clinician supporting the patient to consider each option, before reaching an informed decision [13-15]. Elicitation of patient preferences is considered central to effective SDM [16, 17]. When multiple treatment options exist, each of which may be associated with different risks, benefits and quality of life implications, adherence to SDM steps can result in a decision that is optimal for the individual patient (and their relatives/carers). If evidence for a specific treatment is strong, the clinician may make a recommendation, informing the patient of their reasoning, which the patient may accept or decline [18, 15]. Audiotaped consultations between 236 patients and 40 haematologists in the United States (US) [19] revealed that patient preferences were not commonly elicited, and that treatment recommendations were provided by haematologists in 97% of consultations. More recent studies, (mainly surveys), indicate increasing desire amongst patients with haematological cancers for involvement in treatment decisions [20-

For peer review only - http://bmjopen.bmj.com/site/about/guidelines.xhtml

22], but highlight dissatisfaction with information received. A recent thematic review [15] of 18 haematological studies, indicates three critical, but modifiable, barriers to patient centred communication, a prerequisite for SDM: insufficient information exchange, treatment goal misalignment, and discordant (patient preferred and actual) role preferences in decision making. Despite the recognised need for qualitative research to better understand and contextualise preferences for involvement in decision making amongst haematology patients [15], few qualitative studies have been conducted.

Our study was designed to investigate the perspectives of patients with one of four haematological subtypes: chronic lymphocytic leukaemia (CLL), follicular lymphoma (FL), marginal zone lymphoma (MZL), and myeloma. Specifically, we aimed to explore patients' understanding and experiences of involvement in treatment decision making (TDM), and to identify factors promoting or impeding this process. This study constitutes one strand of a larger programme of work designed to provide evidence-based information about the management and experiences of the general population of patients with chronic haematological malignancies.

METHODS

Methods are reported in accordance with the Consolidated Criteria for Reporting Qualitative Research Checklist [23].

2.1 Study design

A qualitative, descriptive study [24, 25], utilising semi-structured in-depth interviews.

2.2 Sample and setting

The study was conducted within the UK's Haematology Malignancy Research Network (HMRN: www.hmrn.org), a unique collaboration between university academics, National Health Service (NHS) clinicians, and patients and carers, that facilitates research using various methods, with the purpose of generating evidence to underpin improvements in clinical practice. Detailed information about HMRN's configuration, methods and ethical approvals has been published elsewhere [6,7]. HMRN includes a population of around 4 million people in Yorkshire and Humberside and has a similar age and sex profile to the UK and a comparable socio-economic and urban/rural distribution.

Sampling in qualitative research aims to acquire information that is useful for understanding the complexity, depth, variation, or context surrounding a phenomenon [26, 27]. We therefore aimed to

For peer review only - http://bmjopen.bmj.com/site/about/guidelines.xhtml

capture a broad range of diverse experiences; initial criteria included proximity to the median diagnostic age for each disease subtype, with variation by gender, ethnicity and postcode, as well as time since diagnosis. Over time, our sampling strategy evolved to purposively select individuals across broader demographic categories (for example, those relatively young when first diagnosed), and to ensure inclusion of participants at different time points on the clinical pathway. To appreciate the role of caregivers, patients were asked to invite a relative to contribute to the interview, if they wished. Ten relatives were present and contributed to varying extents. Details of the study sample can be seen in Table 1.

2.3 Data collection

Using existing links with NHS teams, initial checks ensured patients were alive and well enough to participate. Potential participants were sent information about the study, and a letter inviting them to take part in an interview, with a reply slip and pre-paid envelope. The researcher's contact details and free-phone number were included so that patients could discuss the study before deciding whether to take part. Thirty-five interviews were conducted (DM) between February and October, 2019; and ten relatives participated. The majority took place privately, in patients' homes and lasted around 40-90 minutes. Interviews were digitally recorded and transcribed verbatim, checked for accuracy and anonymised. Recordings and transcriptions were stored in accordance with legally required data protection standards and ethically approved practices. Interviewing continued until it appeared no new information was forthcoming, a signal that data saturation was likely achieved [28], and the recruitment end-point occurred when preliminary analysis indicated patterns and themes with sufficient data [29].

Interviews were directed by a semi-structured topic guide **(Appendix 1)** based on research literature and input from clinicians (haematology specialist consultants and nurses) and piloted with 2 patients (from a haematology cancer support group) to check comprehensiveness and comprehensibility. The guide was modified over time to include new lines of inquiry, and was used flexibly to allow patients to "tell their story" from diagnosis onwards.

2.4 Data analysis

The analytic approach adopted was qualitative description [24], based on thematic content analysis [30]. Qualitative description research seeks to discover and understand a phenomenon, a process, or the perspectives and worldviews of the people involved [31-33]. Analysis was undertaken by two members of the research team (DM, DH), both experienced in qualitative methods in applied health

For peer review only - http://bmjopen.bmj.com/site/about/guidelines.xhtml

services research and haematology. Interviews were summarised through dynamic engagement with the dataset, while staying close to participants' accounts [24]. Our aim was to translate the 'raw' data into a coherent depiction of the phenomena under scrutiny [34]. Guided by the research questions, our analysis balanced both inductive and deductive orientations [35]. We familiarised ourselves with the content of the transcripts to identify initial codes (units of meaning) and themes. These were modified and expanded during an interactive and reflexive process of 'interrogating' the data, in the search for common patterns and 'deviant' or 'negative' cases not supporting, or appearing to contradict, patterns or explanations emerging from data analysis [36]. Data were then summarised and compared, within and between cases. Our analysis therefore facilitated data synthesis and interpretation, enabling a detailed and nuanced account of the findings [34]. Analytical rigour was promoted through reflective notes and memos, and discussion of disagreements helped refine the analysis [37].

2.5 Ethical considerations

Research projects set within HMRN's infrastructure and collecting supplementary data require ethical approval, which was granted by the London, City and East committee (REC:16/ LO/0740). Assurances of confidentiality and anonymity were given to participants and written consent was obtained from patients and relatives. During interview, a (small) number of patients became upset while reflecting on their cancer and its progression. Although these patients were asked if they wanted to pause or discontinue the interview, all wished to continue, some commenting afterwards that the discussion had helped clarify their thinking about their disease.

2.6 Patient and Public Involvement

Patient public involvement (PPI) is integral to HMRN and lay-individuals are routinely involved in all research activities. For this particular study, patients and relatives were involved in prioritising aims, preparing the funding application, attending programme steering committee meetings, and the dissemination of findings.

RESULTS

Thirty-five patients (19 male, 16 female) were interviewed, 10 accompanied by relatives (spouse/partner or other family member). Most were aged 50-70 years; 32 lived with a spouse/partner or other family member and three lived alone. Ten patients had CLL, eight FL, twelve myeloma and five MZL. Prior to interview, patients had experienced different treatment pathways, according to diagnosis and disease progression; some (7) had started and remained on W&W, others

For peer review only - http://bmjopen.bmj.com/site/about/guidelines.xhtml

(22) had started treatment, and a further group (6) had experienced multiple lines of chemotherapy before progressing to stem cell transplant. Patient characteristics and individuals' treatment pathways, ascertained from HMRN routine data collection, and patient self-report, can be found in Table 1.

For peer review only

Table 1. Characteristics of interviewees

[1] CLL: Chronic lymphocytic leukaemia; FL: Follicular lymphoma; MZL: Marginal zone lymphoma.

[2] Chemotx = Chemotherapy; HPE = Helicobactor pylori eradication; Radiotx = Radiotherapy; SCT = Stem cell transplant (all autografts); SCH = Stem cell harvest (shown for P33 because this patient's SCT was cancelled as it was considered risk by clinical staff and the patient).

[3] Does not include supportive care (e.g. blood product transfusions, plasma exchange, bisphosphonates, cell mobilization products).

[4] Patient was diagnosed pre-HMRN, so data presented were collected at interview.

For peer review only

For peer review only - http://bmjopen.bmj.com/site/about/guidelines.xhtml

Table 1. Characteristics of interviewees

The following 5 themes were identified: 1. Preference for clinician recommendations; 2. Factors implicated in patient involvement in TDM; 3. Perceptions of proactive/non-proactive approaches to TDM; 4. Experiences of TDM at various points in the disease trajectory; 5. Support from others.

Theme 1: Preference for clinician recommendations

Most of the patients in interviewed in our study said they wished to be informed about, and given the opportunity to discuss, treatment benefits, risks and outcomes with their doctor, while expressing a preference for the clinician to make a recommendation. Some said they wanted their consultant to explain the rationale for recommendations, while others said they weren't interested, that they just wanted *'to get it* [treatment] *over and done with'*.

*'they spoke to me so much, I feel involved***…** *I would hate for them to say, you're going to do that, with no explanation…and that doesn't happen. When I have treatment, I am told why.'* (P9)

'I wanted their expertise and their guidance…I felt very involved but I didn't necessarily feel I should be making the ultimate decisions… I wanted them to make the decisions…' (P19)

I would just do what they recommend really' (P23)

'I just like the person telling me what I've got and what they're going to do' (P27)

Preferences for clinician recommendations were said to be based on patients' respect for haematologists' clinical knowledge and expertise, trust in their professional judgement, and faith that they would have the patient's best interests at heart.

'I do have a respect for medical people because they've done all the training, so I would hope they would say… well we think this is probably the best way forward, because I think, well, how would I know?' (P26)

'I've got total faith in what they are doing…I am certainly not capable of making a medical decision on my behalf' (P21)

'the team are making that decision for me, and they're working for me with my best interests at heart' (P13)

For peer review only - http://bmjopen.bmj.com/site/about/guidelines.xhtml

Trust in individual haematologists was strongly linked to patient perceptions of (mainly) excellent patient/doctor relationships, characterised by consultants' willingness and ability to: demonstrate empathy (by helping patients feel at ease, and valued as individuals, *'not just a number'*); tailor information to match individual requirements; listen and respond to questions and concerns; initiate and engage patients in open and frank discussion; and impart hope and some positivity when things are not progressing well.

'she [haematologist] explained everything…I could understand everything…there was no stress involved…she seems to be able to ask the right questions and she takes it all in…she tends not to write down until we've finished talking…we just have a chat basically…she is so warm and pleasant, smiling, we have a laugh…I mean you come away feeling elated rather than 'phew' (P10)

'maybe they just think well, if somebody is not asking at the moment, we won't say, because this is too much information. Maybe they take their lead from where you're at…' (P27)

'giving time for people to actually speak…especially when people are ill, to think about things before they ask questions…so listening is probably at the top of the list' (P29)

'let's have everything as open as possible so that everybody who's in the equation knows what's going on or what possibilities are out there for treatments' (P14)

'I was a bit frightened but she said, if you do exactly as we've planned…you will come out of it…I'm so certain of that. Now, when any consultant tells you that, it sort of lifts you' (P9)

Trust was said to be enhanced when patients knew that treatment discussions had included members of the wider haematology team, as happened within the context of multidisciplinary team meetings, if their consultant had sought a second opinion from haematologists specializing in the patient's condition, and when the patient was aware that they were receiving treatment in a recognised centre of excellence.

'these people [haematologists] are much more expert than I'll ever be…the consultant that is making the decision, it's his team. You've been talked about, so it isn't one person making that decision…in the background there's quite a lot of people that are involved and I find that really, really soothing' (P13)

'it's reassuring that it is not just one person making that decision…when he [haematologist] decided I needed to start chemo, he did say he had actually spoke to a colleague of his at [place] University…I

For peer review only - http://bmjopen.bmj.com/site/about/guidelines.xhtml

think he was a professor…and told him my symptoms, and the professor had also said, well, yes, I think it's time to start treatment…' (P4)

I knew they were a centre of excellence and they had very high standards…so, I just felt very…secure' (P19)

Interestingly, some people revealed awareness of 'their' consultant's clinical and academic credentials.
'he [patient's consultant] was the lead consultant for the team so I thought, well that is a good recommendation' (P24)

'and Prof [name] who I've seen perhaps 4 times and everyone wants to see Prof [name], because he's got the biggest brain [laughs] (P28)

Only one study participant (P3, with CLL) mentioned having themselves sought a second opinion, commenting that far from taking offence, his consultant assisted him in identifying appropriate specialists to contact.

'he [haematologist] didn't take that as a personal insult…you feel more confident…going to see one of the top specialists [second opinion]…you're going to see a range of experts who have agreed this is the best option…you are being backed by a range of experts' (P3)

Theme 2: Factors implicated in patient involvement in TDM
The extent to which participants in our study said they were involved in TDM appeared to vary according to a range of inter-related individual *('everybody is different')* and contextual factors, including decision complexity; individuals' ability and desire to access, interpret, and retain information about their cancer; personal preferences and values; patients' physical and/or emotional state, and coping mechanisms; and the level of support from others (elaborated in Theme 5). These factors were drawn together to develop this theme and are summarised in Figure 1, with quotes (below) illustrating each component. While some patients wanted to hand over much, or all, responsibility to clinicians, others took steps to enable participation in discussions; many interviewees clustered somewhere between, reporting varying levels of engagement/non-involvement, dependent on circumstances.

[Individual differences]: *'every patient is different…there probably are patients who just rely on their specialist…most are happy to just 'get on with it gov', whereas I was always asking questions and*

For peer review only - http://bmjopen.bmj.com/site/about/guidelines.xhtml

that's what I would recommend to other patients, is try to understand and be proactive… that way at least when [there's] any option to decide if I can feel confident that's the best available for me' (P3)

[Decisional complexity]: *'in an ideal world it would be myself, me and the doctors [who share TDM] because to me, they've got so much knowledge about this thing that I don't know anything about… I'd just like to have something [simple] like, Oh, you've got appendicitis'…but I'm not in that situation. It is complex…they don't know all the answers…because there's different types of it [myeloma] and I don't fully get the reasons why everyone reacts differently [to treatment]'* (P35)

[Information access]: *'I'm not an internet person…it annoys me, the internet'* (P15)

[Information understanding]: *'Not everyone is equipped to sort of read some of the literature because…it is a little bit challenging…and time consuming'* (R, P6)

[Information retention]: *'We were given some leaflets…but I'm a bit fuzzy…since I've had my cancer my memory has gone to shot'* (P16)

[Physical/emotional state]: *'I was so rock bottom, I guess I just went along with it all…they knew what they were doing'* (P8)

[Coping mechanism]: *'there are some people who want to know every detail about every treatment and how it affects them…I'm scared to do that'* (P35)

[Personal preference (for proactive role)]: *'PatientView [portal for accessing patient electronic record]…that's fantastic because I can see things, so your platelets, white [blood cells]…haemoglobin…it's a fantastic bit of information for me prior to my consultant appointment…having that extra information, for me, is very valuable…I can point out…to the consultant…so what's this about…you know, why's that gone up?'* (P28)

[Personal circumstances]: *'your mind is concentrating on other things...is it life threatening…am I going to be able to work anymore…have I got to retire…you've got the financial aspects…making sure your family is looked after…your brain is working on so many different levels…you tend to accept what's [treatment] being given'* (P16)

[Level of support]: *'that's what I miss, because I have to do all my own research…and sometimes I think I just wish I had that person… [for support]'* (P33)

Theme 3: Perceptions of proactive/non-proactive approaches to TDM

For peer review only - http://bmjopen.bmj.com/site/about/guidelines.xhtml

During interview, a number of patients articulated views relating to proactive involvement in TDM, within the context of their stated preference for acceptance of haematologists' recommendations, while others described factors likely to impede or diminish active engagement in TDM. Factors associated with proactive and non-proactive approaches to decisions are summarised in Figure 2.

Those who chose to be proactive described the need for certain resources (internet, time), skills (retrieving/interpreting relevant information) and a high level of personal commitment. This group also tended to access information from many sources, including UK charities (for example, Myeloma UK and CLL Support Association), national and local support groups, on-line patient forums (mainly US based), clinical nurse specialists (CNSs), and peer reviewed articles. Some patients described how internet access was essential, along with the ability to distinguish between *'authoritative'* and *'dreadful'* websites. Taking time to read about blood/lymphatic systems was seen as a necessary pre-requisite for understanding specific diseases. Patients who sought in-depth information from journal publications tended to have some prior knowledge which facilitated their understanding, up to the point when they encountered *'the buffers'* of what they could comprehend. Even these patients, who were well equipped to retrieve and assimilate information, reached a limit to their capacity to understand information relating to their haematological cancer at a specific juncture; other study participants indicated that they had difficulty understanding much/most of the information they encountered.

'I did intelligent searches…I was confident and used to reading science papers…' (R, P6)

'I had read on the internet all about these different prognostic indexes and stuff…when I was told prognosis was 5 years…I already knew what prognosis meant' (P34)

'I'd be looking at Lancet type papers…PubMed' (P24)

'it turned out the free light chains had rocketed… so that ruled out second stem cell transplant' (P18)

'it's good to know statistics…I wanted to know about survival figures…I realised that I was looking at the population as a whole, but then I thought I'd look at younger people…' (P3)

'it's challenging because you will reach the buffers at some point, when you think, that's just a little too difficult to understand' (R, P6)

Proactive patients described preparing for consultations. Despite busy clinics, patients said doctors rarely made them feel time was constrained, usually invited questions, and took time to respond. Some patients prepared questions beforehand, a few noted the consultant's response(s), with some

For peer review only - http://bmjopen.bmj.com/site/about/guidelines.xhtml

having relatives acting as 'scribe'. Keeping a record or graph of blood test results, used as a trigger for questions, and maintaining a diary of disease progress and treatments were common. These patients sought results from investigations (blood tests, bone marrow biopsies, X-rays, CT scans, etc.) and often asked consultants to explain their significance. They also read about the risks, benefits and possible side effects of treatments, to equip themselves to engage in further discussion. Being prepared to 'speak up' during consultations was said to be important, though was recognized to be easier for some than others: *'you have to be reasonably assertive, which is maybe not my strength, but I force myself in those situations where I know how important it is'* (P3). Only one patient (P6) and their spouse (R,P6) reported 'speaking up' to express a preference for a treatment other than the one offered by their consultant, based on their own *'research'*, that suggested it would be less detrimental to the patient's quality of life: *'we knew the gold standard was treatment with Fludarabine, FCR as they call it, it really wasn't recommended for people aged over 65, but nonetheless it was on offer to us…we actually then chose the Bendamustine route…the gentle one…which was a good alternative…we'd done our research, we made an intelligent decision'* (R, P6).

Many respondents expressed little or no desire to engage in active TDM, preferring to rely on clinicians' expertise, as reflected, for example, in the following comments: '*I don't want to go into details of things…the people at the hospital are there to help me…I put my absolute trust in them'* (P10); *'I went along with everything that I was asked to do because I have complete confidence in how they were handling the situation for me'* (P14).

Factors that could impede patients adopting a proactive approach included difficulties accessing and/or fully understanding information, a disinclination to dwell on disease progression and prognosis *('in denial'*: P35*)*, or patients feeling unwell, anxious, overwhelmed, or unsupported.

'I've got a little tablet but I very rarely use it. I don't like to, I'm not computer literate' (P30)

'the specialist at the hospital said, it's only forty-four, which meant nothing to me, forty-four what? She didn't say what and I never asked her.' (P31)

'sometimes they come out with all these big words and then you think, I'm not sure what that word is' (P24)

For peer review only - http://bmjopen.bmj.com/site/about/guidelines.xhtml

'I have glanced through it [booklet about myeloma] but it's a bit too high a level…it needs to be basic' (P21)

'I saw this research about myeloma…and when I read the title I wasn't sure whether it was going to be that helpful, because I didn't understand what it was really…' (P35)

'psychologically, I brush it under the carpet a bit because I know it [chemotherapy] is not imminent' (P5)

'I've just gone back to being anxious again…it's just horrible being in this position where you know it's [paraprotein level] is creeping up' (P35)

'I am strong, you know, but it just becomes too much… I want somebody just to hold my hand and go, I'm going to sort that for you.' (P33)

Theme 4: Experiences of TDM at various points in the disease trajectory

Patients' accounts indicated that the potential and desire for involvement in TDM could vary or indeed be curtailed at different points in the disease trajectory. Most participants described feeling deeply shocked, upset and anxious when first informed of their diagnosis (*'like a huge bombshell'*), to the extent that they could not absorb what was being said to them or think of questions to ask (*'it's as if your brain switched off'*), reactions likely to compromise ability to participate in discussions about treatment options.

'we were floored…[by diagnosis]…it hit us out of the blue…you can't think of questions in that short space of time…we were speechless…we just sat there in shock…you hear the word cancer…'' (P32)

'it was a shock when he mentioned the word cancer…it just sort of shut me down…he was willing to give me information but at that time, I just couldn't process it' (P4)

Some patients on W&W, who subsequently went on to require treatment, reported little or no involvement in TDM about the type of therapy to be given, as there was only a single relevant option. In this context the decision was said to be *'automatic'* and that it would be the *'standard'* treatment. Some of these participants said they would have welcomed having more time to discuss the proposed treatment with family members, and to consider whether or not to accept it; a few recalled their consultant strongly recommending that they accept the treatment offered.

For peer review only - http://bmjopen.bmj.com/site/about/guidelines.xhtml

'It was a fait accompli really' (P15)

'I was told that I had to have chemotherapy, 6 chemotherapies…one every 3 weeks and radiotherapy after that' (P8)

'I was very worried and scared and thought I might choose not to have the treatment…because I felt physically very well…why subject myself to being ill' (P1)

'[consultant said] "I must tell you that even though you don't want to go on chemo….my recommendation is that you do"' (P9).

Opportunities for involvement in decision making appeared circumscribed when urgent treatment was required. Examples came from P19, who was admitted to hospital acutely ill and subsequently diagnosed with FL, from P24, who said he was diagnosed with *'a very unusual lymphoma',* and from P21, with myeloma. In these instances, each patient referred to TDM occurring at speed, largely without their involvement, as they relied on the expertise of the clinical team to make the *'best'* decisions on their behalf.

'There weren't really any options…it was a case of this is what is best for you… I didn't have time to think about it… it [the cancer] was so advanced…they decided… I don't think I was really involved in that…I wanted them to guide me and make the decisions' (P19)

'it was a very unusual type of lymphoma…there was a huge team involved in it all and even throughout their decision making they changed from what were originally going to do, which was radiotherapy...they were just going to blitz it, but my kidney would have been in the way, so they then decided to go down the avenue of chemotherapy and the monoclonal…' (P24)

'they [doctors] decided on stem cell transplant straightaway' (P21)

Disease progression resulted in some patients with myeloma feeling overwhelmed when faced with difficult treatment decisions, and unable to choose between options. Factors compounding this included the intensive nature of proposed treatments (such as stem cell transplant) and their impact on quality of life; the limited "returns" that some treatments seemed to offer, compared to the consequences of associated risks, such as infection; and the uncertainty and unpredictability of outcomes.

For peer review only - http://bmjopen.bmj.com/site/about/guidelines.xhtml

'Honestly, my head is exploding with all this…it's just like a big crushing thing to me…I think I am quite strong but this is doing me in' (P35)

'the big one [decision] was the second stem cell transplant…I was really struggling to make the decision as to whether I wanted to go for it' (P18)

'they [doctors] sort of said, well the average remission after the stem cell transplant is, I think either 12-24 months, or 18-24 months, something like that, and there was I thinking, right I'm going for the 10-year option, so that was quite a shock' (P18)

'Myeloma is a very individual disease. You get the same treatment, same, same this, same that but you have different outcomes and things.' (P28)

Theme 5: Support from others

Our interviewees said that their relatives often accompanied them to clinical consultations, and they were portrayed as playing an important, and sometimes crucial, role when treatment options were being discussed and considered: *'she [patient's wife] guided me…she would translate…she would talk to the ward people…'* (P7). Patients benefitted from *'going over'* information from their haematologist with their relative following consultations, and discussing the details of any proposed treatment, side effects, and implications for quality of life with them: *'we'd come home and discuss it [information from the consultant] what she said about so and so…having two sets of ears helped'* (P11). Some patients (for example, those who mentioned difficulties processing and retaining information) described relying heavily on support from relatives (spouse/partner/adult child/sibling) for all interactions with clinicians: *'my daughter always takes notes…so when we come away we can go through them…they are quite happy with that…if I go on my own, I would retain some of it and I'd probably forget some of it…my daughter knows all about my treatment'* (P9).

Relatives' roles encompassed gathering and interpreting information, acting as a sounding board, and providing practical and emotional support to patients preparing for, and undergoing, treatment.

*'we were proactive and looked for information' (*R,P7)

'we'd [patient and partner] talked through it [decision related to stem cell transplant] over a number of weeks really…we'd come back to it…there's this factor and that factor…' (P18)

'there are times when my wife has come to the rescue…particularly if I get an infection…I'm thankful there is somebody else around' (P11)

For peer review only - http://bmjopen.bmj.com/site/about/guidelines.xhtml

Many patients and relatives used the pronoun *'we'* throughout the interview, though there was general agreement that the final decision of whether or not to accept a treatment, was/would be the patients.

'we'd both be involved…it wouldn't be one partner on their own, it would always be the two of us involved together' (P21)

'he [husband] doesn't sway me, he leaves it very much up to me, he wouldn't persuade me…' (P1)

'I'm as involved as much as I can be but at the end of the day it's [patient's name]… he has to make the decision, I can't make it for him' (R, P31)

'she [partner] kind of left me to make the decision really, but we'd talked through it over a number of weeks' (P18)

The importance of emotional support provided by a spouse/partner to patients experiencing anxiety and inner turmoil associated with diagnosis and TDM was repeatedly emphasised: *'she's [patient's wife] been with me at every step'* (P27); *'you need somebody to be the rock…to take the strain off you'* (P16); *'I don't know how I could have coped without him [husband]'* (P6).

Three study participants living alone commented that they often felt unsupported in relation to their cancer, feelings that were heightened when treatment decisions arose: *'I knew chemo was what I'd have to have but when he* [consultant] *told me, that shocked me, and I was so upset and scared…really scared'* (P4). In the absence of a spouse/partner they turned to friends or close family members for advice and support, but did not want to be a burden to them: *'I've got a very good friend…we've known each other 50 years…I would never have got through it without her…you need support…somebody talk to…but she has her own family…I don't like to be a burden to anybody'* (P8). Two of the three participants without a spouse/partner mentioned seeking formal psychological support, so that they might have the opportunity to share and discuss their feelings and experiences.

Eight of the 35 participants had joined a formal support group. Reported benefits included hearing about up-to-date research findings from invited speakers (often clinicians), and having the opportunity to talk to other people about their experiences, which helped some patients think through advantages and disadvantages of their own options for treatment. Those who preferred not to join a group *('not for me'), ('I'm a very private person'),* often sought one-to-one support, through meetings with former patients arranged by clinicians, personal contacts with someone with a similar diagnosis, and/or via on-line patient forums.

For peer review only - http://bmjopen.bmj.com/site/about/guidelines.xhtml

DISCUSSION

This qualitative study provides new insights into patient perspectives of involvement in treatment decisions, their views on proactive engagement, and the role of others in supporting decision making. The findings reflect the broad array of interconnected mechanisms at play in shared decision making [38], and its complex and dynamic nature [39-41]. The principal finding among our interviewees was the strong preference for treatment recommendations to be provided by haematologists, based on trust in their clinical expertise and perceptions of empathetic patient/clinician relationships. Most participants expected/wanted an explanation from their clinician about the rationale for treatment decisions, including details of possible risks and benefits, but did not wish, and/or felt ill-equipped, to make decisions on their own behalf. This finding does not align with some recent reports of the growing desire for involvement amongst patients with haematological malignancies [20-22], though seems unsurprising within the detailed contexts depicted by our interviewees. Participants with myeloma, who had most decisions to make due to the nature of their cancer, tended to experience decision making as challenging, struggling to weigh up the risks, adverse impacts on quality of life, and prognostic uncertainty associated with different treatments options; which left them inclined to follow clinician recommendations. Patients with FL also reported trusting clinicians to make decisions on their behalf, particularly when they were acutely ill at diagnosis (as may occur in some of these typically indolent cancers), and unable to participate in discussions. Likewise, participants with CLL were mainly inclined to entrust decisions to clinicians; however, some expressed dissatisfaction that they had not been given the opportunity to fully consider, and accept, or decline, treatment. Dissatisfaction amongst this group of patients was possibly, in part, attributable to the fact that patients with CLL often had infrequent contact with haematology HCPs during long periods of observation, with months sometimes elapsing between appointments.

Drawing on results from their systematic review of the literature relating to physician views of SDM, Pollard et al. [42] comment that physicians tend to express support for SDM in situations where they do not feel strongly about one treatment alternative, but are less supportive of SDM in situations where compelling, or well-evidenced, clinical practice guidelines exist in favour of one treatment over another. In such instances, the decision is not one of selecting between options, but rather whether the patient chooses to accept or decline treatment. This review includes results from an interview study with 20 physicians working in five different settings [43], that found support for SDM was most common among those who had received communication skills training in this area; and Rocque et al. [44] suggest that multi-level education programmes, targeting patients with CLL and their clinicians, may improve patient participation in decision making.

For peer review only - http://bmjopen.bmj.com/site/about/guidelines.xhtml

Patient involvement in decision making has been linked to improved care experiences and better health outcomes, yet the desire for this has been shown to vary by individuals, number of treatment options and treatment certainty [45]. That patients with haematological malignancies, which are characterised by uncertain trajectories, indistinct transitions and prognostic uncertainty [46, 47, 7] and novel and evolving treatments, may prefer to defer decisions to specialists, whom they trust, is understandable. Our findings resonate with qualitative studies in Germany [48] and Denmark [49], that combine interviews with extensive observation of consultations with clinicians and patients with cancer, and which show that most of the time physicians made treatment decisions alone, or with colleagues, with little patient involvement. None of our participants recalled clinicians formally eliciting their preferences regarding decision-making, as is recommended [50, 51], yet most felt as involved in this process as they wanted to be, through discussions of treatment options, and clinicians taking time to talk and listen to them, address their concerns, answer questions, and offer explanations. Many of the patients in our study clearly felt strong bonds with their specialists, as noted elsewhere [47], arising from sustained contact in clinic or during hospital admissions. A meta-ethnography of quantitative and qualitative studies [52] underscores the importance patients place on being in a caring relationship with clinicians, which may preclude the need to seek detailed information.

Amongst our participants was a small number (n=5) who shared certain characteristics (male, (mainly) younger, educated to degree level, and highly motivated), and who had adopted a proactive approach to TDM. These individuals generally felt confident interpreting complex information, and were prepared to 'speak up' to obtain further explanation from clinicians; nonetheless, limits to understanding were perceived as constraining their ability to make fully informed decisions. Loh et al. [53] caution against predicting preference for decisional involvement of patients with haematological cancers, based on age or characteristics such as educational attainment, suggesting instead that this should be assessed periodically, as part of decision-making encounters.

While many respondents preferred little or no involvement in TDM, there were some whose engagement was hindered by the provision of information that did not match their needs, leaving them feeling ill-equipped to deal with the complex nature of this material. Clinician assessment of individual health literacy (capacity to access, process and interpret information) is therefore an essential component of SDM, as is noted by others [15]. Providing patients with information that is comprehensible, tailored to their needs, and which does not overwhelm [54], can be challenging. Strategies for 'drip-feeding' [54] information, are to be preferred to a one-way-flow of information from clinician to patient ('broadcasting'), which is regarded as suboptimal [55]. Interestingly,

For peer review only - http://bmjopen.bmj.com/site/about/guidelines.xhtml

insufficient time for information sharing and discussion during consultations was not generally perceived as a problem in our study, though is reported elsewhere [15, 48, 20].

During interview, patients often recalled feelings of profound shock on hearing their diagnosis, reactions that could act as a barrier to meaningful discussion about treatment. A qualitative study [56] including 32 patients with acute myeloid leukaemia revealed similar findings; highlighting the importance of clinicians managing the amount of information patients are ready to receive [54], both at diagnosis and other timepoints; for example, initiation of treatment after W&W.

The important role played by relatives and others in supporting patients during consultations when treatment options are discussed was very apparent in our study, reflecting results from a systematic review [57] of patient-physician-companion communication that shows companions as instrumental in information transfer and provision of emotional support. Unaccompanied patients in our study said they particularly benefitted from a nurse taking notes of what was said during consultation, and 'talking through' the record with them afterwards. Interviewees without a spouse/partner expressed concerns about burdening friends and family members with their needs for practical and emotional support. While some patients may be reluctant to broach the topic of their cancer with others, or find it difficult to talk about it, they are likely to benefit from discussing treatment options/decisions with someone close to them, who is familiar with their personal preferences and circumstances; haematology doctors and nurses could take time to help patients identify such individuals, and encourage patients to draw on their support.

Coping with disease progression and prognostic uncertainty was said to be particularly difficult by our participants with myeloma. Treatment with SCT (or the newer CAR T-cell therapy) can affect patients physically, psychologically and financially [58-60], and formal psychological support may be beneficial [61, 62]. Several of those considering SCT valued one-to-one support, as also noted by Tariman [20]. Furthermore, it has been suggested that information about 'personal experiences' can complement 'general facts', and contribute to decision support in various ways, for example, by helping people clarify their own values and reasoning, either by suggesting different ways of thinking and/or by providing a 'sounding board' against which to test their own ideas [63].

Strengths and Limitations

As far as we are aware, this is the first UK study to specifically explore involvement in TDM in patients with haematological malignancies. Our sample size of 35 enabled in-depth exploration of the research questions, and use of semi-structured interviews allowed participants to focus on issues they themselves considered significant. Our sampling framework ensured 'key informants' were

For peer review only - http://bmjopen.bmj.com/site/about/guidelines.xhtml

interviewed from targeted disease subtypes, both sexes and various age-groups. As the diseases included are typically relapsing-remitting conditions, we felt it important to include patients whose perceptions may have altered over time, during prolonged W&W or following treatment, to capture as broad a range of views as possible. To counteract the influence of memory, we also invited some recently diagnosed patients to take part; reference to patient diaries and contributions from relatives also enhanced recall. Relatives' participation enhanced the quality of the data collected, through contribution of their own perspectives, prompting patients, and, on occasion, corroboration and/or clarification of patients' accounts.

Attempts to recruit patients from black and minority ethnic (BAME) backgrounds were unfortunately unsuccessful. As Morse [64] has highlighted how merging data from a small number of BAME participants can result in loss of cultural differences when analysed alongside the remainder, who share a single identity, we recommend future in-depth studies, dedicated to those whose heritage differs from participants in our own study. Furthermore, fewer people living in more deprived areas agreed to take part, compared to those in affluent areas. Consequently, a further limitation was our inability to recruit patients with low levels of literacy, who may have been deterred because the study invitation and information was provided in writing. We therefore recognise that the views of our participants are unlikely to reflect those of the entire population with the diseases of interest. Nonetheless, it is highly likely that a large proportion of our findings are transferable to other UK areas, and also countries with similar health-care infrastructure and universal health-care coverage.

Clinical Implications

Our findings suggest that our interviewees varied in their preference for involvement in TDM according to intrinsic, contextual, and disease-related factors, requiring clinicians to assess individuals' preferences for engagement at multiple time points over the course of their haematological cancer pathway. Fisher et al. [41] comment that clinicians who clarify patients' preferences and ensure they are informed about their options, are sharing the deliberation aspect of decision making, even if the doctor ultimately provides a strong recommendation. Entrusting clinical staff to make recommendations does not appear to diminish patients' desire for discussion of possible options, and for provision of relevant information that matches their individual needs. Empathetic relationships with clinicians seem highly valued by patients, and appear conducive to engagement in TDM. Deliberation of treatment options can be highly distressing for some patients, and those lacking support from family members/others may benefit from formal assessment and referral for psychological support.

For peer review only - http://bmjopen.bmj.com/site/about/guidelines.xhtml

CONCLUSION

This study revealed that patients with haematological cancers may wish to be involved in TDM to varying extents, contingent on complex, inter-related factors, that are dynamic and subject to change according to clinical and personal contexts. Overall, our interviewees expressed a strong preference for acceptance of clinician recommendations, linked to disease complexity, patients' trust in clinician expertise, and perceptions of trusted patient-clinician relationships.

For peer review only

For peer review only - http://bmjopen.bmj.com/site/about/guidelines.xhtml

Abbreviations and Declarations

Abbreviations

TDM: Treatment decision making; NHS: National Health Service; UK: United Kingdom; P: Patient; R: Relative.

Declarations

Ethics approval and consent to participate

Research projects set within HMRN's infrastructure and collecting supplementary data require ethical approval, which was granted by the London, City and East committee (REC:16/ LO/0740). Assurances of confidentiality and anonymity were given to participants and written consent was obtained from patients and relatives.

Consent for publication

All interviewees consented to the use of quotations from their interviews in publications arising from the study.

Availability of data and materials

All data and materials relating to this research are from the Haematological Malignancy Research Network and are archived and maintained by the first and last author, according to organisational and ethical regulations. Data are not publicly available due to the risk of participant identification from specific contexts revealed when reading entire transcripts and due to the terms and conditions regarding the release of data to third parties upon which ethical approvals for this study were contingent. Reasonable requests for further information relating to this data can be made to the corresponding author.

Competing interests

The authors declare that they have no competing interests.

Funding

This work was supported by the NIHR via a PGfAR: RP-PG-0613-2002, Cancer Research UK: 29685, and Blood Cancer UK (formerly Bloodwise): 15037. None of the funding bodies were involved in the design of the study, nor in the collection, analysis, interpretation and reporting of data; the views expressed here are those of the authors and do not necessarily reflect those of the funder.

Authors' contributions

DH, ER, AS and RP designed the study. AS identified potential participants and mapped pathways. DH and DM recruited the study participants, and DM conducted interviews. Transcripts were coded and analysed by DM with discussion/input from DH. DM wrote the first draft of the manuscript. DH, ER, AS revised the manuscript. RP commented on the clinical aspects of the study. All authors read and approved the final version.

Acknowledgments

We wish to thank the study participants who took part in an interview and shared sensitive and emotive issues.

For peer review only - http://bmjopen.bmj.com/site/about/guidelines.xhtml

REFERENCES:

1. Office for National Statistics. Cancer Registration Statistics, England: 2017. https://www.ons.gov.uk/peoplepopulationandcommunity/healthandsocialcare/conditionsanddiseases/bulletins/cancerregistrationstatisticsengland/2017 (release date April, 2019) Accessed 15 Jan 2021.

2. Public Health England National Statistics Cancer Registration Statistics, England: final release, 2018. Gov.UK (Published 29 May, 2020). https://www.gov.uk/government/publications/cancer-registration-statistics-england-2018-final-release/cancer-registration-statistics-england-final-release-2018. Accessed 15 Jan 2021.

3. Blood Cancer UK, 2018. https://bloodcancer.org.uk/news/latest-statistics Accessed 15 Jan 2021.

4. National Institute for Clinical Excellence (NICE) Interactive Flowcharts. 2020. https://pathways.nice.org.uk/pathways/ Accessed 15 Jan 2021.

5. British Society for Haematology. Guidelines. 2020. https://b-s-h.org.uk/guidelines/ Accessed 15 Jan 2021.

6. Smith A, Howell D, Patmore R, Jack A, Roman E. Incidence of haematological malignancy by sub-type: a report from the Haematological Malignancy Research Network. Br J Cancer. 2011;105:1684–92. Available from: https://doi.org/10.1038/bjc.2011.450.

7. Smith A, Howell D, Crouch S, Painter D, Blase J, Wang H-I, et al. Cohort Profile: The Haematological Malignancy Research Network (HMRN): a UK population-based patient cohort. Int J Epidemiol. 2018; 7:700-700g. Available from: https://doi.org/10.1093/ije/dyy044.

8. Im A, Pavletic SZ. Immunotherapy in hematologic malignancies: past, present, and future. J Hematol Oncol 2017;10:94. Available from: https://doi.org/10.1186/s13045-017-0453-8.

9. Gauthier J, Yakoub-Agha I. Chimeric antigen-receptor T-cell therapy for hematological malignancies and solid tumors: Clinical data to date, current limitations and perspectives. Curr Res Transl Med.2017;65:93-102. Available from: https://doi.org/10.1016/j.retram.2017.08.03.

10. Institute of Medicine (US) Committee on Quality of Health Care in America. Crossing the Quality Chasm: a new Health System for the 21st Century. Washington (DC): Natl Acad Press (US); 2001. Available from: https://doi.org/10.17226/10027.

For peer review only - http://bmjopen.bmj.com/site/about/guidelines.xhtml

11. Coulter A, Collins A. Making Shared Decision Making a Reality: No decision about me, without me. Report from The King's Fund. 2011; London: The King's Fund. Available from: https://www.kingsfund.org.uk/sites/default/files/Making-shared-decision-making-a-reality-paper-Angela-Coulter-Alf-Collins-July-2011_0.pdf. Accessed 15 Jan 2021.

12. Barry M, Edgman-Levitan S. Shared Decision Making – the Pinnacle of Patient-Centred Care. New Engl J Med. 2012;366(9):780-1. Available from: https://doi.org/10.1056/NEJMp1109283.

13. Elwyn G, Frosch D, Thomson R, Joseph-Williams N, Lloyd A, Kinnersley P, Cording E, Tomson D, Dodd C, Rollnick S, Edwards A, Barry M. Shared Decision Making: A Model for Clinical Practice. J Gen Intern Med. 2012;27(10):1361–7 Available from: https://doi.org/10.1007/s11606-012-2077-6.

14. Elwyn G, Durand MA, Song J, Aarts J, Barr PJ, Berger Z, et al. A three-talk model for shared decision making: multistage consultation process. BMJ. 2017;359:j4891 Available from: http://dx.doi.org/10.1136/bmj.j4891

15. LeBlanc TW, Baile WF, Eggly S, Bylund CL, Kurtin S, Khurana M, et al. Review of the patient-centered communication landscape in multiple myeloma and other hematologic malignancies. Pat Educ Couns. 2019;102:1602-1612. Available from: https://doi.org/10.1016/j.pec.2019.04.028.

16. Mulley AG, Trimble C, Elwyn G. Stop the silent misdiagnosis: patients' preferences matter. BMJ. 2012;345:e6572. Available from: https://doi.org/10.1136/bmj.e6572.

17. Elwyn G, Cochran N, Pignone M. Shared Decision Making – the Importance of Diagnosing Preferences. JAMA Intern Med. 2017;177(9):1239-1240. Available from: https://doi.org/10.1001/jamainternmed.2017.1923.

18. Bomhof-Roordink H, Fischer MJ, van Duijn-Bakker N, Baas-Thijssen MC, van der Weijden T, Stiggelbout AM, Pietersei AH. Shared decision making in oncology: A model based on patients', healthcare professionals' and researchers' views. Psycho-Oncology. 2019;28 (1):139–146. Available from: https://doi.org/10.1002/pon.4923.

19. Alexander SC, Sullivan AM, Back AL, Tulsky JA, Goldman RE, Block SD, et al. Information giving and receiving in haematology malignancy consultations. Psycho-Oncology. 2012;21(3):297–306. Available from: https://doi.org/10.1002/pon.1891.

20. Tariman JD, Doorenbos A, Schepp KG, Becker PS, Berry DL. Patient, Physician and Contextual Factors Are Influential in the Treatment Decision Making of Older Adults Newly Diagnosed with

For peer review only - http://bmjopen.bmj.com/site/about/guidelines.xhtml

Symptomatic Myeloma. Cancer Treat Commun. 2014;2:34-47. Available from: https://doi.org/10.1016/j.ctrc.2014.08.003.

21. Rood JAJ, van Zuuren, FJ, Stam F, van der Ploeg T, Eeltink C, Verdonck-de Leeuws IM, Huigens PC. Perceived need for information among patients with a haematological malignancy: associations with information satisfaction and treatment decision-making preferences. Hematol Oncol. 2015;33 (2): 85–98. Available from: https://doi.org.10.1002/hon.2138.

22. Rood JAJ, Nauta IH, Witte BI, Stam F, van Zuuren FJ, Manenschijn A, et al. Shared decision-making and providing information among newly diagnosed patients with haematological malignancies and their informal care-givers: Not "one size fits all". Psycho-Oncology. 2017;26(12):2040–2047. Available from: https://doi.org/10.1002/pon.4414.

23. Tong A, Sainsbury P, Craig J. Consolidated criteria for reporting qualitative research (COREQ): a 32-item checklist for interviews and focus groups. Int J Qual Health Care. 2007; 19(6):349-57. Available from: https://doi.org/10.1093/intqhc/mzm042.

24. Sandelowski M. 2000. Focus on research methods: Whatever happened to qualitative description? Res Nurs Health. 23(4):334-340. Available from: https://doi.org/10.1002/1098-240x(200008)23:4<334::aid-nur9>3.0.co;2-g.

25. Neergaard MA, Olesen F, Andersen RS, Sondergaard J. Qualitative description-the poor cousin of health research? BMC Med Res Methodol. 2009;9:52. Available from: https://doi.org/10.1186/1471-2288-9-52.

26. Gentles SJ, Charles C, Ploeg J, McKibbon KA. Sampling in Qualitative Research: Insights from an Overview of the Methods Literature. The Qualitative Report. 2015; 20:1772-1789. Available from: http://nsuworks.nova.edu/tqr/vol20/iss11/5

27. Malterud K, Siersma VD, Guassora AD. Sample size in qualitative interview studies: Guided by information power. Qual Health Res. 2016; 26(13):1753–1760. Available from: https://doi.org/10.1177/1049732315617444.

28. Saunders B, Sim J, Kingstone T, Baker S, Waterfield J, Bartlam B, et al. Saturation in qualitative research: Exploring its conceptualization and operationalization. Qual Quant. 2018; 52(4):1893–1907. Available from: https://doi.org/10.1007/s11135-017-0574-8.

For peer review only - http://bmjopen.bmj.com/site/about/guidelines.xhtml

29. Thorne, S. The Great Saturation Debate: What the "S Word" Means and Doesn't Mean in Qualitative Research Reporting. Can J Nurs Res. 2020; 52: 3–5. Available from: https://doi.org/10.1177/0844562119898554.

30. Morse J. Confusing categories and themes. (Editorial). Qual Health Res. 2008;18 (6):727-728. Available from: https://doi.org/10.1177/1049732308314930.

31. Bradshaw C, Atkinson S, Doody O. Employing a Qualitative Description Approach in Health Care Research. Glob Qual Nurs Res. 2017;24 (4):2333393617742282. Available from: https://doi.org/10.1177/2333393617742282.

32. Caelli K, Ray L, Mill, J. "Clear as mud": Toward greater clarity in generic qualitative research. Int J Qual Methods. 2003;2:1–23. Available from: https://doi.org/10.1177/160940690300200201.

33. Pope C, Mays N. (eds). Qualitative Research in Health Care. 3rd ed. Oxford: BMJ Blackwell Publishing; 2006.

34. Sandelowski, M. (2010). What's in a name? Qualitative description revisited. Res Nurs Health. 33(1):77–84. Available from: https://doi.org/10.1002/nur.20362.

35. Green J, Thorogood N. Qualitative Methods for Health Research: Introducing Qualitative Methods Series. 4th ed. London: Sage; 2018, p258.

36. Pope C, Ziebland S, Mays N. Analysing qualitative data. BMJ. 2000;8;320 (7227): 114–116. Available from: https://doi.org/10.1136/bmj.320.7227.114.

37. Barbour RS. 2001. Checklists for improving rigour in qualitative research: a case of the tail wagging the dog? BMJ. 2001;322:1115–1117. Available from: https://doi.org/10.1136/bmj.322.7294.1115.

38. Waldron T, Carr T, McMullen L, Westorp G, Duncan V, Neufeld S-M, Bandura L-A, Groot G. Development of a Programme Theory for Shared Decision Making: a realist synthesis. BMC Health Serv Res. 2020;20(1):59. Available from: https://doi.org/10.1186/s12913-019-4649-1.

39. Entwistle VA, Watt IS. Patient involvement in treatment decision making: the case for a broader conceptual framework. Pat Ed Couns. 2006;63(3):268-278. Available from: https://doi.org/10.1016/j.pec.2006.05.002.

40. Joseph-Williams N, Elwyn G, Edwards, A. Knowledge is *not* power for patients: A systematic review and thematic synthesis of patient-reported barriers and facilitators to shared decision

For peer review only - http://bmjopen.bmj.com/site/about/guidelines.xhtml

making. Pat Educ Couns. 2014.94(3):291-309. Available from: https://doi.org/10.1016/j.pec.2013.10.031.

41. Fisher KA, Tan ASL, Matlock DD, Saver B, Mazor KM, Pieterse AH. Keeping the patient in the center: Common challenges in the practice of shared decision making. Pat Educ Couns. 2018;101(12):2195–2201. Available from: https://doi.org/10.1016/j.pec.2018.08.007.

42. Pollard S, Bansback N, Bryan S. Physician attitudes toward shared decision making: A systematic Review. Pat Educ Couns. 2015;98(9):1046-1057. Available from: https://doi.org/j.pec.2015.05.004.

43. Zeuner R, Frosch DL, Kuzemchak MD, Politi MC. Physicians' perceptions of shared decision-making behaviours: a qualitative study demonstrating the continued chasm between aspirations and clinical practice. Health Expect. 2014;18(6):2465–2476. Available from: https://doi.org/10.1111/hex.12216.

44. Rocque GB, Williams CP, Halilova KI, Borate U, Jackson BE, Van Laar ES, et al. Improving shared decision making in chronic lymphocytic leukaemia through multidisciplinary education. Trans Behav Med. 2018;8:175-182. Available from: https://doi.org/10.1093/tbm/ibx034.

45. Blumenthal-Barby JS. 'That's the doctor's job': Overcoming patient reluctance to be involved in medical decision making. Pat Educ Couns. 2017; 100(1):14-17. Available from: https://doi.org/10.1016/j.pec.2016.07.010.

46. National Institute for Clinical Excellence (NICE). Haematological cancers: improving outcomes NICE guideline [NG47]. https://www.nice.org.uk/guidance/ng47/evidence/improving-outcomes-in-haematological-cancers-the-manual-pdf-2487893581. Accessed 15 Jan 2021.

47. McCaughan D, Roman E, Smith AG, Garry A, Johnson M, Patmore R, et al. Determinants of hospital death in haematological cancers: findings from a qualitative study. BMJ Supp Palliat Care. 2018;8(1):78–86. Available from: https://doi.org/10.1136/bmjspcare-2016-001289.

48. Hahlweg P, Harter M, Nestoriuc Y, Scholl I. How are decisions made in cancer care? A qualitative study using participant observation of current practice. BMJ Open. 2017;7(9):e016360. Available from: https://doi.org/10.1136/bmjopen-2017-016360.

49. Lowe MM, Osther PJS, Ammentorp J, Birkelund R. The Balance of Patient Involvement: Patients' and Health Professionals' Perspectives on Decision-Making in the Treatment of Advanced Prostate Cancer. Qual Health Res. 2021;31(1):29-40. Available from: https://doi.org/10.1177/1049732320962759.

For peer review only - http://bmjopen.bmj.com/site/about/guidelines.xhtml

50. Coulter A. Patient information and shared decision-making in cancer care. Br J Cancer. 2003;89 Suppl 1:S15-16. Available from: https://doi.org/10.1038/sj.bjc.6601080.

51. Legare F, Stacey D, and IP TEAM. IP-SDM model. 2010. https://decisionaid.ohri.ca/docs/develop/IP-SDM-Model.pdf Accessed 15 Jan 2021.

52. Atherton K, Young B, Salmon P. Understanding the information needs of people with haematological cancers. A meta-ethnography of quantitative and qualitative research. Eur J Cancer Care. 2017;26(6):e12647. Available from: https://doi.org/10.1111/ecc.12647.

53. Loh KP, Tsang M, LeBlanc TW, Back A, Duberstein PR, Mohile SG, et al., Decisional involvement and information preferences of patients with hematologic malignancies. Blood Adv. 2020; 4(21):5492-5500. Available from: https://doi.org/10.1182/bloodadvances.2020003044.

54. Atherton K, Young B, Kalakonda N, Salmon P. Perspectives of patients with haematological cancer on how clinicians meet their information needs: “Managing” information versus “giving” it. Psycho-Oncology. 2018;27(7):1719–1726. Available from: https://doi.org/10.1002/pon.4714.

55. Chhabra KR, Pollack KI, Lee SJ, Back AL, Goldman RE, Tulsky JA. Physician Communication Styles in Initial Consultations for Hematological Cancer. Pat Educ Couns. 2013; 93(3):573-8. Available from: https://doi.org/10.1016/j.pec.2013.08.023.

56. LeBlanc TW, Fish LJ, Bloom CT, El-Jawahri A, Davis DM, Locke SC, et al. Patient experiences of acute myeloid leukemia: A qualitative study about diagnosis, illness understanding, and treatment decision-making. Psycho-Oncology. 2017;26(12):2063–2068. Available from: https://doi.org/10.1002/pon.4309.

57. Laidsaar-Powell RC, Butow PN, Bu S, Charles C, Gafni A, Lam WWT, et al. Physician–patient–companion communication and decision-making: a systematic review of triadic medical consultations. Patient Educ Couns. 2013; 91(1):3–13. Available from: https://doi.org/10.1016/j.pec.2012.11.007.

58. Mosher CE, DuHamel KN, Rini C, Corner G, Lam J, Redd WH. Quality of Life Concerns and Depression among Hematopoietic Stem Cell Transplant Survivors. Support Care Cancer. 2011; 19(9): 1357–1365. Available from: https://doi.org.10.1007/s00520-010-0958-y.

For peer review only - http://bmjopen.bmj.com/site/about/guidelines.xhtml

59. Walpole G, Clark H, Dowling M. Myeloma patients' experiences of haematopoietic stem cell transplant: A qualitative thematic synthesis. Eur J Onc Nurs. 2018;35:15-21. Available from: https://doi.org/10.1016/j.ejon.2018.05.002.

60. Ruark J, Mullane E, Cleary N, Cordeiro A, Bezzero ED, Wu V, et al., Patient-Reported Neuropsychiatric Outcomes of Long-Term Survivors after Chimeric Antigen Receptor T Cell Therapy. Biol Blood Marrow Transplant. 2020; 26(1):34-43. Available from: https://doi.org/10.1016j.bbmt.2019.09.037.

61. Swash B, Hulbert-Williams N, Bramwell R. Unmet psychosocial needs in haematological cancer: a systematic review. Support Care Cancer. 2014; 22(4):1131-1141. Available from: https://doi.org/10.1007/s00520-014-2123-5.

62. Oberoi DV, White VM, Seymour JF, Prince HM, Harrison S, Jefford M et al. Distress and unmet needs during treatment and quality of life in early cancer survivorship: A longitudinal study of haematological cancer patients. Eur J Haematol. 2017; 99(5):423-430. Available from: https://doi.org/10.1111/ejh.12941.

63. Entwistle VA, France EF, Wyke S, Jepson R, Hunt K, Ziebland S, Thompson A. How information about other people's personal experiences can help with healthcare decision-making: a qualitative study. Patient Educ Couns. 2011;85(3):e291–8. Available from: https://doi.org/10.1016/j.pec.2011.05.014.

64. Morse J. The Changing Face of Qualitative Inquiry. Invited IIQM Conference Keynote address 2019. Int J Qual Methods. 2020; 19:1–7. Available from: https://doi.org/10.1177/1609406920909938.

For peer review only - http://bmjopen.bmj.com/site/about/guidelines.xhtml

Figure 1. Factors implicated in patient involvement in treatment decisions

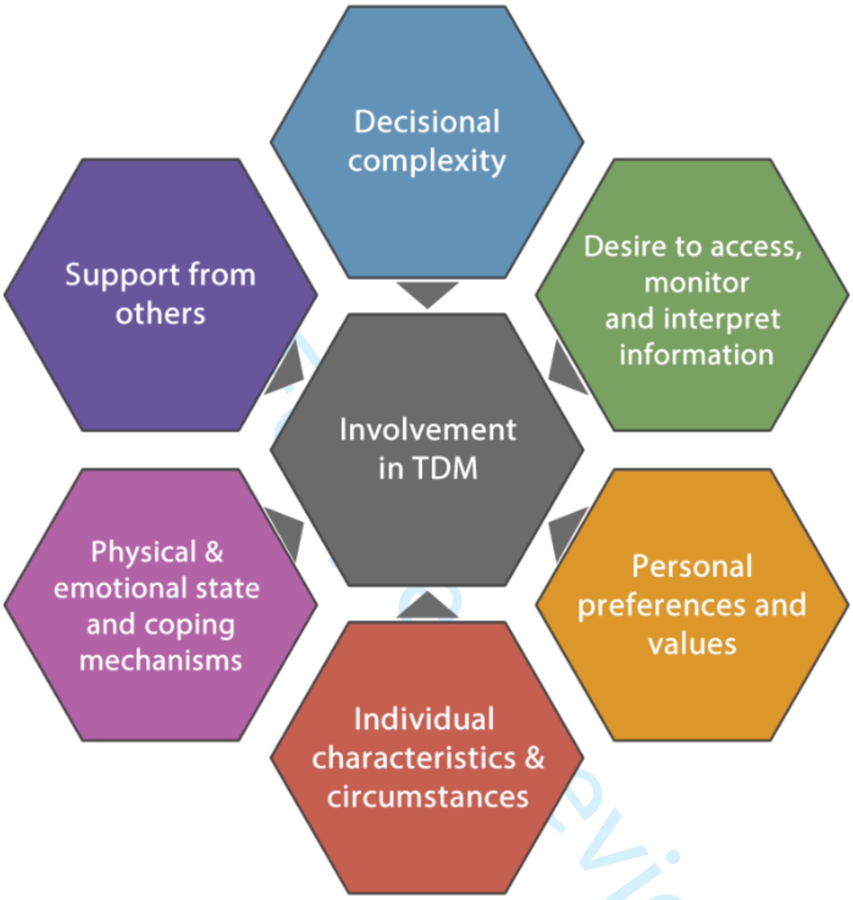

Figure 2. Continuum of characteristics associated with proactive and non-proactive approaches to involvement in treatment decisions

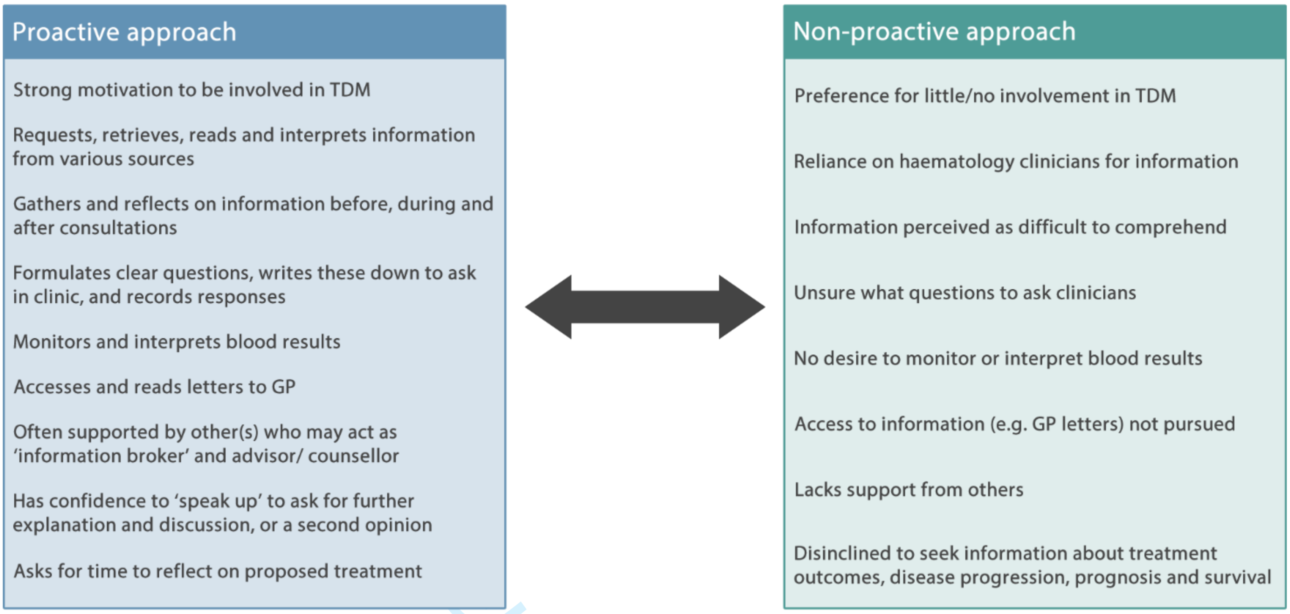

r review only

Supplementary file 1: Topic guide for interviews with patients and relatives

Focusing on information and decisions at key states (diagnosis, W&W, treatment) and progression through states.

Information

- How important is it to you that you receive information about your cancer? *(why is that?)*
- How do you feel about the information given to you at diagnosis/start of treatment?
- How do you feel about getting information from HCPs more generally? *(time constraints; overwhelming; difficult to understand/take in; use of language/terminology)*
- Do you feel the information given applies specifically to you? *(personalized, tailored, specific)*
- How healthcare practitioners (HCPs) ascertain your information needs?
- Is the information you received explained in a way you can understand? *(technical language; too detailed; not detailed enough)*
- What do HCPs do to check if you understand the information they give you?
- How do you feel about asking questions? Are your questions always answered?
- Do you feel that your information needs are usually met? What worked well and could have been better? *(diagnosis; treatment initiation/cessation - examples)*
- What do you think about the timing of information from HCPs? When is the right time? *(at diagnosis; during clinic appointments; when disease status changes; at other times)*
- How do/did you feel about discussing the risks/benefits of different treatments with HCPs?
- How do you feel about discussing prognosis? *("a statement about expectations that refers to the likely course of the cancer and/or outcome") (want to know/not; timing; language)*
- What strategies do you use to absorb information? (*in general, how bad news is processed*)

Treatment decisions

- How do you feel about being involved in decisions with HCPs about your treatment?
- Have you been asked you if you want to be involved in decisions about treatment?
- Do you want to be involved in decisions? *(preference for patient only; clinician only; patient/clinician)*
- What should be considered during treatment decision making? *(effectiveness of treatment; side effects; prognosis; patient goals, values, preferences; impact on quality of life)*
- What might make it easier or harder for you to be involved in making decisions about your treatment? *(time; style of communication; how information is conveyed; explanations)*
- Are there particular time-points when it is harder to be involved in making decisions about treatment? *(diagnosis; treatment initiation/change; treatment cessation)*

Practical issues

- How do you feel about the amount of information you get? *(prefer more/less; overloaded; struggle to absorb)*
- What do you want to know/know more about? *(investigations; treatments; prognosis; side effects; QoL)*
- Where/who do you prefer to get information; why? *(Internet; doctors/nurses; family; leaflets; support group)*
- What do you think about different sources of information? *(credibility, ability to judge)*
- How do you prefer to see information about risks and benefits; why? (w*ords/numbers; figures/percentages; diagrams/graphs)*

For peer review only

COREQ (COnsolidated criteria for REporting Qualitative research) Checklist

Topic	No.	Guide Questions/Description	Page	Explanation
**Domain 1: Research team and reflexivity**				
**Personal characteristics**				
Interviewer	1	Which author conducted the interview?	p5	Dorothy McCaughan (DM)
Credentials	2	What were the researcher's credentials? E.g. PhD, MD	n/a	BA, RN MSc
Occupation	3	What was their occupation at the time of the study?	n/a	Senior Research Fellow
Gender	4	Was the researcher male or female?	n/a	Female
Experience and training	5	What experience or training did the researcher have?	n/a	Registered Nurse. Conducted many qualitative interviews patients.
**Relationship with participants**				
Relationship established	6	Was a relationship established prior to the study?	n/a	No relationship existed with participants prior to the study.
Participant knowledge of the interviewer	7	What did participants know about the researcher? e.g. personal goals, reasons for doing the research?	n/a	Participants had no knowledge of the interviewer prior to the study.
Interviewer characteristics	8	What characteristics were reported about the interviewer? e.g. Bias, assumptions, interests in topic	p5	Data were collected by DM, analysed by DM & DH, both qualified nurses and experienced health services researchers.
**Domain 2: Study design**				
**Theoretical framework**				
Methodological orientation and theory	9	What methodological orientation underpinned the study? e.g. grounded theory, content analysis	p5	Qualitative description and thematic content analysis (referenced in the Methods).
**Participant selection**				
Sampling	10	How were participants selected? e.g. purposive, convenience, consecutive,	p4	Interviewees were purposively selected.
Method of approach	11	How were participants approached?	p5	Interviewees were recruited by mail. Potential participants contacted DM if they wanted to take part.
Sample size	12	How many participants were in the study?	p5	Thirty-five patient interviews were undertaken, ten included relatives.
Non-participation	13	How many people refused to participate or dropped out? Reasons?	n/a	After invitation, around 20 patients did not contact the study team; reasons for this are unknown. No-one who responded dropped out.
Setting of data collection	14	Where was the data collected? e.g. home, clinic, workplace	p5	Data were largely collected in patient homes. A small number took place in the University or elsewhere.
Presence of nonparticipants	15	Was anyone else present besides the participants and researchers?	p5	Interviews were conducted privately.

For peer review only

Description of sample	16	What are the important characteristics of the sample? e.g. demographic data, date	p4<br>Table 1	The sample was selected from HMRN and included patients with chronic haematological malignancies, who had agreed they could be contacted for research (men and women of any ethnicity and age, living in any area).
Interview guide	17	Were questions, prompts, guides provided by the authors? Was it pilot tested?	p5<br>Appx. 1	A semi-structured topic guide, tested on 2 patients, was developed from the literature and team experiences.
Repeat interviews	18	Were repeat interviews carried out? If yes, how many?	n/a	No, there were no repeat interviews.
Audio/visual recording	19	Was audio or visual recording used to collect the data?	p5	Audio recording was used.
Field notes	20	Were field notes made during and/or after the interview?	n/a	Field notes were made post-interview.
Duration	21	What was the duration of the interviews?	p5	Interviews lasted 40-90 minutes.
Data saturation	22	Was data saturation discussed?	p5	Interviews continued until saturation appeared to have been reached.
Transcripts returned	23	Were transcripts returned to participants for comment and/or correction?	n/a	This was not requested and audiotaping was clear.
**Domain 3: analysis and findings**				
**Data analysis**				
Number of data coders	24	How many data coders coded the data?	p5	DM & DH were involved in coding and discussed/developed the scheme.
Description of the coding tree	25	Did authors provide a description of the coding tree?	p5-6<br>Fig. 1	The coding scheme (tree) reflected the topic guide and was developed to accommodate data content.
Derivation of themes	26	Were themes identified in advance or derived from the data?	p5	Themes were derived from the data. The topic guide provided a framework for data collection.
Software	27	What software, if applicable, was used to manage the data?	n/a	Data were managed in Word and Excel.
Participant checking	28	Did participants provide feedback on the findings?	n/a	Feedback was provided to patients via the study newsletter.
**Reporting**				
Quotations presented	29	-Were quotations presented to illustrate themes/findings?<br>-Were quotations linked to ID?	p7-16	Quotations are presented with anonymised IDs, linked to more detailed characteristics in Table 1.
Data and findings consistent	30	Was there consistency between data and findings?	p7-16	We consistently link findings with supporting quotations.
Clarity of major themes	31	Were major themes clearly presented in the findings?	p7-16<br>Fig. 1&2	Five major themes are clearly defined, with separate headings.
Clarity of minor themes	32	Is there a description of diverse cases or discussion of minor themes?	p5, p19	Whilst highlighting the main themes, we also incorporated a range of responses and drew attention to negative cases.

Developed from: Tong A, Sainsbury P, Craig J. Consolidated criteria for reporting qualitative research (COREQ): a 32-item checklist for interviews and focus groups. International Journal for Quality in Health Care. 2007. Volume 19, Number 6: pp. 349 – 357.

For peer review only - http://bmjopen.bmj.com/site/about/guidelines.xhtml

BMJ Open

Treatment decision making (TDM): A qualitative study exploring the perspectives of patients with chronic haematological cancers

Journal:	*BMJ Open*
Manuscript ID	bmjopen-2021-050816.R2
Article Type:	Original research
Date Submitted by the Author:	10-Nov-2021
Complete List of Authors:	McCaughan, Dorothy; University of York, Health Sciences<br>Roman, Eve; University of York, Health Sciences<br>Smith, Alexandra; University of York, Health Sciences<br>Patmore, Russell; Castle Hill Hospital, Queens Centre for Oncology<br>Howell, Debra; University of York, Health Sciences
**Primary Subject Heading**:	Haematology (incl blood transfusion)
Secondary Subject Heading:	Health services research, Patient-centred medicine, Qualitative research
Keywords:	Myeloma < HAEMATOLOGY, Leukaemia < HAEMATOLOGY, Lymphoma < HAEMATOLOGY, QUALITATIVE RESEARCH, Organisation of health services < HEALTH SERVICES ADMINISTRATION & MANAGEMENT, CHEMOTHERAPY

SCHOLARONE™
Manuscripts

I, the Submitting Author has the right to grant and does grant on behalf of all authors of the Work (as defined in the below author licence), an exclusive licence and/or a non-exclusive licence for contributions from authors who are: i) UK Crown employees; ii) where BMJ has agreed a CC-BY licence shall apply, and/or iii) in accordance with the terms applicable for US Federal Government officers or employees acting as part of their official duties; on a worldwide, perpetual, irrevocable, royalty-free basis to BMJ Publishing Group Ltd ("BMJ") its licensees and where the relevant Journal is co-owned by BMJ to the co-owners of the Journal, to publish the Work in this journal and any other BMJ products and to exploit all rights, as set out in our licence.

The Submitting Author accepts and understands that any supply made under these terms is made by BMJ to the Submitting Author unless you are acting as an employee on behalf of your employer or a postgraduate student of an affiliated institution which is paying any applicable article publishing charge ("APC") for Open Access articles. Where the Submitting Author wishes to make the Work available on an Open Access basis (and intends to pay the relevant APC), the terms of reuse of such Open Access shall be governed by a Creative Commons licence – details of these licences and which Creative Commons licence will apply to this Work are set out in our licence referred to above.

Other than as permitted in any relevant BMJ Author's Self Archiving Policies, I confirm this Work has not been accepted for publication elsewhere, is not being considered for publication elsewhere and does not duplicate material already published. I confirm all authors consent to publication of this Work and authorise the granting of this licence.

For peer review only

TITLE: Treatment decision making (TDM): A qualitative study exploring the perspectives of patients with chronic haematological cancers

AUTHORS: Dorothy McCaughan, Eve Roman, Alexandra Smith, Russell Patmore, Debra Howell

CORRESPONDING AUTHOR:

Debra Howell
Epidemiology & Cancer Statistics Group, Department of Health Sciences, Seebohm Rowntree Building, University of York, York, UK. YO10 5DD.
Telephone: +44 (0)904 321895
Email: debra.howell@york.ac.uk

AUTHORS
Dorothy McCaughan
Epidemiology & Cancer Statistics Group, Department of Health Sciences, Seebohm Rowntree Building, University of York, York, UK. YO10 5DD.
Telephone: +44 (0) 904 321348
Email: dorothy.mccaughan@york.ac.uk

Eve Roman
Epidemiology & Cancer Statistics Group, Department of Health Sciences, Seebohm Rowntree Building, University of York, York, UK. YO10 5DD.
Telephone: +44 (0) 904 321882
Email: eve.roman@york.ac.uk

Alexandra Smith
Epidemiology & Cancer Statistics Group, Department of Health Sciences, Seebohm Rowntree Building, University of York, York, UK. YO10 5DD.
Telephone: + 44 (0) 904 321884

Russell Patmore
Queens Centre for Oncology and Haematology, Castle Hill Hospital, Cottingham, UK.
Telephone: +44 (0) 482 604444
Email: Russell.Patmore@hey.nhs.uk

Debra Howell
Epidemiology & Cancer Statistics Group, Department of Health Sciences, Seebohm Rowntree Building, University of York, York, UK. YO10 5DD
Telephone: +44 (0)904 321895
Email: debra.howell@york.ac.uk

KEYWORDS: leukaemia, lymphoma, myeloma, treatment decision making, qualitative research

WORD COUNT: 6212 including quotations

For peer review only - http://bmjopen.bmj.com/site/about/guidelines.xhtml

ABSTRACT

Objectives

Haematological malignancies are the fifth most common cancer in the UK, with chronic subtypes comprising around a third of all new diagnoses. These complex diseases have some similarities with other cancers, but often require different management. Surgical resection is not possible, and while some are curable with intensive chemotherapy, most indolent subtypes are managed with non-aggressive intermittent or continuous treatment, often over many years. Little is known about the views of patients with chronic haematological cancers regarding treatment decision making (TDM), a deficit our study aimed to address.

Setting and design

Set within the Haematological Malignancy Research Network (HMRN: www.hmrn.org), an ongoing population-based cohort that provides infrastructure to support evidence-based research, HMRN data were augmented by qualitative information from in-depth interviews. Data were analysed for thematic content, combining inductive and deductive approaches. Interpretation involved seeking meaning, salience and connections within data.

Participants

Thirty-five patients with four chronic subtypes: chronic lymphocytic leukaemia, follicular lymphoma, marginal zone lymphoma, and myeloma. Ten relatives were present and contributed to varying extents.

Results

Five themes were discerned: 1. Preference for clinician recommendations; 2. Factors implicated in patient involvement in TDM; 3. Perceptions of proactive/non-proactive approaches to TDM; 4. Experiences of TDM at various points in the disease trajectory; 5. Support from others. Our principal finding relates to a strong preference among interviewees for treatment recommendations from haematologists, based on trust in their expertise and perceptions of empathetic patient-clinician relationships.

Conclusion

Interviewees wanted to be involved in TDM to varying extents, contingent on complex, inter-related factors, that are dynamic and subject to change according to differing clinical and personal contexts. Patients may benefit from clinicians assessing their shifting preferences for involvement on multiple occasions. Strong preferences for acceptance of recommendations was associated with cancer complexity, trust in clinician expertise, and positive perceptions of patient-clinician relationships.

For peer review only - http://bmjopen.bmj.com/site/about/guidelines.xhtml

Strengths and limitations

- Set within the infrastructure of an established population-based study, this is (to our knowledge) the first UK study to specifically explore TDM involvement in patients with chronic haematological malignancies.
- Interview data supplemented robust clinical information routinely collected as part of the broader cohort.
- The sample size (35 patients) enabled in-depth exploration of the research questions, enhanced by contributions from relatives/carers.
- Purposive sampling ensured inclusion of individuals with differing demographic and disease profiles, diagnosed at different time points on the clinical pathway.
- The views of participants are unlikely to reflect those of the entire patient population, and dedicated studies of people from black and minority ethnic backgrounds and those with low literacy levels are required.

For peer review only

For peer review only - http://bmjopen.bmj.com/site/about/guidelines.xhtml

TITLE: Treatment decision making: A qualitative study exploring the perspectives of patients with chronic haematological cancers

BACKGROUND

Haematological malignancies are the fifth most common cancer in the UK [1-3]. They are complex diseases, and although they have some similarities with other cancers, they often require different management. Surgery is not an option, and while some subtypes are curable with intensive chemotherapy and periods of hospitalisation, other more chronic subtypes are not. These more indolent malignancies, which account for around 30% of all newly diagnosed haematological cancers [4, 5], can, however, often be controlled for long periods; typically (but not always) following remitting/relapsing pathways wherein periods of chemotherapy are interspersed with active monitoring, known as Watch and Wait (W&W) [6, 7]. These pathways are associated with variations in the extent of hospital activity, with periods of disease progression, relapse and treatment requiring more consultations and decisions, and W&W having fewer contact points, often months apart. Haemato-oncology is a rapidly changing field, and the past decade has seen the development of innovative treatment regimens that include targeted therapies, along with new and established chemotherapies, radiotherapy, and haematopoietic stem cell transplantation (SCT) [8]. While obviously beneficial, these developments increase treatment decisional complexity for clinicians and patients [9].

Shared decision making (SDM), seen as a hallmark of quality care, is part of a broader concept of patient-centred care which considers individual preferences, needs, and values, with the aim of ensuring patient values guide all clinical decisions [10-12]. Steps integral to SDM include the clinician informing the patient of treatment options and the need for a decision; discussion between patient and clinician of each option; and the clinician supporting the patient to consider each option, before reaching an informed decision [13-15]. Elicitation of patient preferences is considered central to effective SDM [16, 17]. When multiple treatment options exist, each of which may be associated with different risks, benefits and quality of life implications, adherence to SDM steps can result in a decision that is optimal for the individual patient (and their relatives/carers). If evidence for a specific treatment is strong, the clinician may make a recommendation, informing the patient of their reasoning, which the patient may accept or decline [18, 15]. Audiotaped consultations between 236 patients and 40 haematologists in the United States (US) [19] revealed that patient preferences were not commonly elicited, and that treatment recommendations were provided by haematologists in 97% of consultations. More recent studies, (mainly surveys), indicate increasing desire amongst patients with haematological cancers for involvement in treatment decisions [20-

For peer review only - http://bmjopen.bmj.com/site/about/guidelines.xhtml

22], but highlight dissatisfaction with information received. A recent thematic review [15] of 18 haematological studies, indicates three critical, but modifiable, barriers to patient centred communication, a prerequisite for SDM: insufficient information exchange, treatment goal misalignment, and discordant (patient preferred and actual) role preferences in decision making. Despite the recognised need for qualitative research to better understand and contextualise preferences for involvement in decision making amongst haematology patients [15], few qualitative studies have been conducted.

Our study was designed to investigate the perspectives of patients with one of four haematological subtypes: chronic lymphocytic leukaemia (CLL), follicular lymphoma (FL), marginal zone lymphoma (MZL), and myeloma. Specifically, we aimed to explore patients' understanding and experiences of involvement in treatment decision making (TDM), and to identify factors promoting or impeding this process. This study constitutes one strand of a larger programme of work designed to provide evidence-based information about the management and experiences of the general population of patients with chronic haematological malignancies. A suite of further papers are under preparation, which address information seeking and sharing, patients' experiences of disease management, and needs for support.

METHODS

Methods are reported in accordance with the Consolidated Criteria for Reporting Qualitative Research Checklist [23].

2.1 Study design

A qualitative, descriptive study [24, 25], utilising semi-structured in-depth interviews.

2.2 Sample and setting

The study was conducted within the UK's Haematology Malignancy Research Network (HMRN: www.hmrn.org), a unique collaboration between university academics, National Health Service (NHS) clinicians, and patients and carers, that facilitates research using various methods, with the purpose of generating evidence to underpin improvements in clinical practice. Detailed information about HMRN's configuration, methods and ethical approvals has been published elsewhere [6,7]. HMRN includes a population of around 4 million people in Yorkshire and Humberside and has a similar age and sex profile to the UK and a comparable socio-economic and urban/rural distribution.

For peer review only - http://bmjopen.bmj.com/site/about/guidelines.xhtml

Sampling in qualitative research aims to acquire information that is useful for understanding the complexity, depth, variation, or context surrounding a phenomenon [26, 27]. We therefore aimed to capture a broad range of diverse experiences; initial criteria included proximity to the median diagnostic age for each disease subtype, with variation by gender, ethnicity and postcode, as well as time since diagnosis. Over time, our sampling strategy evolved to purposively select individuals across broader demographic categories (for example, those relatively young when first diagnosed), and to ensure inclusion of participants at different time points on the clinical pathway. To appreciate the role of caregivers, patients were asked to invite a relative to contribute to the interview, if they wished. Ten relatives were present and contributed to varying extents. Details of the study sample can be seen in Table 1.

2.3 Data collection

Using existing links with NHS teams, initial checks ensured patients were alive and well enough to participate. Potential participants were sent information about the study, and a letter inviting them to take part in an interview, with a reply slip and pre-paid envelope. The researcher's contact details and free-phone number were included so that patients could discuss the study before deciding whether to take part. Thirty-five interviews were conducted (DM) between February and October, 2019; and ten relatives participated. The majority took place privately, in patients' homes and lasted around 40-90 minutes. Interviews were digitally recorded and transcribed verbatim, checked for accuracy and anonymised. Recordings and transcriptions were stored in accordance with legally required data protection standards and ethically approved practices. Interviewing continued until it appeared no new information was forthcoming, a signal that data saturation was likely achieved [28], and the recruitment end-point occurred when preliminary analysis indicated patterns and themes with sufficient data [29].

Interviews were directed by a semi-structured topic guide **(Appendix 1)** based on research literature and input from clinicians (haematology specialist consultants and nurses) and piloted with 2 patients (from a haematology cancer support group) to check comprehensiveness and comprehensibility. The guide was modified over time to include new lines of inquiry, and was used flexibly to allow patients to "tell their story" from diagnosis onwards.

2.4 Data analysis

The analytic approach adopted was qualitative description [24], based on thematic content analysis [30]. Qualitative description research seeks to discover and understand a phenomenon, a process, or

For peer review only - http://bmjopen.bmj.com/site/about/guidelines.xhtml

the perspectives and worldviews of the people involved [31-33]. Analysis was undertaken by two members of the research team (DM, DH), both experienced in qualitative methods in applied health services research and haematology. Interviews were summarised through dynamic engagement with the dataset, while staying close to participants' accounts [24]. Our aim was to translate the 'raw' data into a coherent depiction of the phenomena under scrutiny [34]. Guided by the research questions, our analysis balanced both inductive and deductive orientations [35]. We familiarised ourselves with the content of the transcripts to identify initial codes (units of meaning) and themes. These were modified and expanded during an interactive and reflexive process of 'interrogating' the data, in the search for common patterns and 'deviant' or 'negative' cases not supporting, or appearing to contradict, patterns or explanations emerging from data analysis [36]. Data were then summarised and compared, within and between cases. Our analysis therefore facilitated data synthesis and interpretation, enabling a detailed and nuanced account of the findings [34]. Analytical rigour was promoted through reflective notes and memos, and discussion of disagreements helped refine the analysis [37].

2.5 Ethical considerations

Research projects set within HMRN's infrastructure and collecting supplementary data require ethical approval, which was granted by the London, City and East committee (REC:16/ LO/0740). Assurances of confidentiality and anonymity were given to participants and written consent was obtained from patients and relatives. During interview, a (small) number of patients became upset while reflecting on their cancer and its progression. Although these patients were asked if they wanted to pause or discontinue the interview, all wished to continue, some commenting afterwards that the discussion had helped clarify their thinking about their disease.

2.6 Patient and Public Involvement

Patient public involvement (PPI) is integral to HMRN and lay-individuals are routinely involved in all research activities. For this particular study, patients and relatives were involved in prioritising aims, preparing the funding application, attending programme steering committee meetings, and the dissemination of findings.

RESULTS

Thirty-five patients (19 male, 16 female) were interviewed, 10 accompanied by relatives (spouse/partner or other family member). Most were aged 50-70 years; 32 lived with a spouse/partner or other family member and three lived alone. Ten patients had CLL, eight FL, twelve

For peer review only

myeloma and five MZL. Prior to interview, patients had experienced different treatment pathways, according to diagnosis and disease progression; some (7) had started and remained on W&W, others (22) had started treatment, and a further group (6) had experienced multiple lines of chemotherapy before progressing to stem cell transplant. Patient characteristics and individuals' treatment pathways, ascertained from HMRN routine data collection, and patient self-report, can be found in Table 1.

For peer review only

For peer review only - http://bmjopen.bmj.com/site/about/guidelines.xhtml

Table 1. Characteristics of interviewees

ID	Diagnosis[1]	Age-range at interview (years)	Years since diagnosis	Known treatment line(s) at interview (via Haematolgoical Malignancy Research Network data)[2,3]					
				1	2	3	4	5	6
P1	CLL	60-70	4	Observation	-	-	-	-	-
P2	SMZL	60-70	15	Observation	Chemotx	Observation	-	-	-
P3	CLL	60-70	22	Observation	Chemotx	Observation	-	-	-
P4	SMZL	60-70	3	Observation	-	-	-	-	-
P5	EMZL	50-60	2	HPE	Observation	-	-	-	-
P6*	CLL	70-80	8	Observation	Chemotx	Observation	-	-	-
P7*	CLL	60-70	6	Observation	Chemotx	Observation	-	-	-
P8	FL	70-80	3	Chemotx	Radiotx	Observation	-	-	-
P9	CLL	80-90	5	Observation	-	-	-	-	-
P10	FL	70-80	8	Observation	Chemotx	Chemotx	Chemotx	-	-
P11	Myeloma	60-70	10	Observation	Chemotx	Observation	-	-	-
P12	SMZL	70-80	5	Observation	-	-	-	-	-
P13	CLL	50-60	1	Observation	-	-	-	-	-
P14	Myeloma	60-70	4	Steroids	Radiotx	Chemotx	Chemotx	Chemotx	SCT
P15	FL	70-80	3	Observation	-	-	-	-	-
P16	Myeloma	60-70	2	Chemotx	Chemotx	Chemotx	SCT	Observation	-
P17*	FL	60-70	3	Observation	-	-	-	-	-
P18	Myeloma	60-70	3	Chemotx	Chemotx	Chemotx	SCT	Observation	
P19	FL	50-60	3	Steroids	Chemotx	Chemotx	Observation	-	-
P20*	CLL	70-80	4	Observation	-	-	-	-	-
P21*	Myeloma	70-80	3	Steroids	Chemotx	Chemotx	Chemotx	SCT	-
P22*	CLL	70-80	3	Observation	Clinical trial	Observation	-	-	-
P23	Myeloma	60-70	3	Observation	-	-	-	-	-
P24	FL	50-60	4	Steroids	Chemotx	Radiotx	Observation	-	-
P25	FL	60-70	4	Chemotx	Chemotx	-	-	-	-
P26	Myeloma	70-80	4	Observation	-	-	-	-	-
P27*	CLL	70-80	4	Chemotx	Observation	-	-	-	-
P28	Myeloma	60-70	4	Steroids	Chemotx	Chemotx	SCT	Clinical trial	Chemotx
P29	CLL	70-80	3	Clinical trial	Observation	-	-	-	-
P30*	Myeloma	70-80	2	Observation	-	-	-	-	-
P31*	Myeloma	70-80	2	Radiotx	Steroids	Chemotx	Observation	-	-
P32*	SMZL	60-70	2	Observation	Chemotx	Observation	-	-	-
P33	Myeloma	50-60	3	Chemotx	Chemotx	SCH	Observation	-	-
P34	FL	50-60	4	Steroids	Chemotx	Chemotx	Chemotx	-	-
P35	Myeloma	50-60	2	Chemotx	Chemotx	Chemotx	Chemotx	SCT	Observation

[1] CLL – chronic lymphocytic leukaemia; FL – follicular lymphoma; SMZL – systemic marginal zone lymphoma; EMZL – extra-nodal marginal zone lymphoma.
[2] Chemotx = Chemotherapy; HPE = H. Pylori eradication; Radiotx = Radiotherapy; SCT = Stem cell transplant (all autografts); SCH = Stem cell harvest (shown as SCT did not take place).
[3] Does not include supportive care (e.g. blood product transfusions, plasma exchange, bisphosphonates, cell mobilization products)
*Relative present at interview

For peer review only

For peer review only - http://bmjopen.bmj.com/site/about/guidelines.xhtml

The following 5 themes were identified: 1. Preference for clinician recommendations; 2. Factors implicated in patient involvement in TDM; 3. Perceptions of proactive/non-proactive approaches to TDM; 4. Experiences of TDM at various points in the disease trajectory; 5. Support from others.

Theme 1: Preference for clinician recommendations

Most of the patients in interviewed in our study said they wished to be informed about, and given the opportunity to discuss, treatment benefits, risks and outcomes with their doctor, while expressing a preference for the clinician to make a recommendation. Some said they wanted their consultant to explain the rationale for recommendations, while others said they weren't interested, that they just wanted *'to get it* [treatment] *over and done with'*.

*'they spoke to me so much, I feel involved**...** I would hate for them to say, you're going to do that, with no explanation…and that doesn't happen. When I have treatment, I am told why.'* (P9)

'I wanted their expertise and their guidance…I felt very involved but I didn't necessarily feel I should be making the ultimate decisions… I wanted them to make the decisions…' (P19)

I would just do what they recommend really' (P23)

'I just like the person telling me what I've got and what they're going to do' (P27)

Preferences for clinician recommendations were said to be based on patients' respect for haematologists' clinical knowledge and expertise, trust in their professional judgement, and faith that they would have the patient's best interests at heart.

'I do have a respect for medical people because they've done all the training, so I would hope they would say… well we think this is probably the best way forward, because I think, well, how would I know?' (P26)

'I've got total faith in what they are doing…I am certainly not capable of making a medical decision on my behalf' (P21)

'the team are making that decision for me, and they're working for me with my best interests at heart' (P13)

Trust in individual haematologists was strongly linked to patient perceptions of (mainly) excellent patient/doctor relationships, characterised by consultants' willingness and ability to: demonstrate

For peer review only - http://bmjopen.bmj.com/site/about/guidelines.xhtml

empathy (by helping patients feel at ease, and valued as individuals, *'not just a number'*); tailor information to match individual requirements; listen and respond to questions and concerns; initiate and engage patients in open and frank discussion; and impart hope and some positivity when things are not progressing well.

'she [haematologist] explained everything…I could understand everything…there was no stress involved…she seems to be able to ask the right questions and she takes it all in…she tends not to write down until we've finished talking…we just have a chat basically…she is so warm and pleasant, smiling, we have a laugh…I mean you come away feeling elated rather than 'phew' (P10)

'maybe they just think well, if somebody is not asking at the moment, we won't say, because this is too much information. Maybe they take their lead from where you're at…' (P27)

'giving time for people to actually speak…especially when people are ill, to think about things before they ask questions…so listening is probably at the top of the list' (P29)

'let's have everything as open as possible so that everybody who's in the equation knows what's going on or what possibilities are out there for treatments' (P14)

'I was a bit frightened but she said, if you do exactly as we've planned…you will come out of it…I'm so certain of that. Now, when any consultant tells you that, it sort of lifts you' (P9)

Trust was said to be enhanced when patients knew that treatment discussions had included members of the wider haematology team, as happened within the context of multidisciplinary team meetings, if their consultant had sought a second opinion from haematologists specializing in the patient's condition, and when the patient was aware that they were receiving treatment in a recognised centre of excellence.

'these people [haematologists] are much more expert than I'll ever be…the consultant that is making the decision, it's his team. You've been talked about, so it isn't one person making that decision…in the background there's quite a lot of people that are involved and I find that really, really soothing' (P13)

'it's reassuring that it is not just one person making that decision…when he [haematologist] decided I needed to start chemo, he did say he had actually spoke to a colleague of his at [place] University…I think he was a professor…and told him my symptoms, and the professor had also said, well, yes, I think it's time to start treatment…' (P4)

For peer review only - http://bmjopen.bmj.com/site/about/guidelines.xhtml

I knew they were a centre of excellence and they had very high standards…so, I just felt very…secure' (P19)

Interestingly, some people revealed awareness of 'their' consultant's clinical and academic credentials.

'he [patient's consultant] was the lead consultant for the team so I thought, well that is a good recommendation' (P24)

'and Prof [name] who I've seen perhaps 4 times and everyone wants to see Prof [name], because he's got the biggest brain [laughs] (P28)

Only one study participant (P3, with CLL) mentioned having themselves sought a second opinion, commenting that far from taking offence, his consultant assisted him in identifying appropriate specialists to contact.

'he [haematologist] didn't take that as a personal insult…you feel more confident…going to see one of the top specialists [second opinion]…you're going to see a range of experts who have agreed this is the best option…you are being backed by a range of experts' (P3)

Theme 2: Factors implicated in patient involvement in TDM

The extent to which participants in our study said they were involved in TDM appeared to vary according to a range of inter-related individual *('everybody is different')* and contextual factors, including decision complexity; individuals' ability and desire to access, interpret, and retain information about their cancer; personal preferences and values; patients' physical and/or emotional state, and coping mechanisms; and the level of support from others (elaborated in Theme 5). These factors were drawn together to develop this theme and are summarised in Figure 1, with quotes (below) illustrating each component. While some patients wanted to hand over much, or all, responsibility to clinicians, others took steps to enable participation in discussions; many interviewees clustered somewhere between, reporting varying levels of engagement/non-involvement, dependent on circumstances.

[Individual differences]: *'every patient is different…there probably are patients who just rely on their specialist…most are happy to just 'get on with it gov', whereas I was always asking questions and that's what I would recommend to other patients, is try to understand and be proactive… that way at least when [there's] any option to decide if I can feel confident that's the best available for me'* (P3)

For peer review only - http://bmjopen.bmj.com/site/about/guidelines.xhtml

[Decisional complexity]: *'in an ideal world it would be myself, me and the doctors [who share TDM] because to me, they've got so much knowledge about this thing that I don't know anything about… I'd just like to have something [simple] like, Oh, you've got appendicitis'…but I'm not in that situation. It is complex…they don't know all the answers…because there's different types of it [myeloma] and I don't fully get the reasons why everyone reacts differently [to treatment]'* (P35)

[Information access]: *'I'm not an internet person…it annoys me, the internet'* (P15)

[Information understanding]: *'Not everyone is equipped to sort of read some of the literature because…it is a little bit challenging…and time consuming'* (R, P6)

[Information retention]: *'We were given some leaflets…but I'm a bit fuzzy…since I've had my cancer my memory has gone to shot'* (P16)

[Physical/emotional state]: *'I was so rock bottom, I guess I just went along with it all…they knew what they were doing'* (P8)

[Coping mechanism]: *'there are some people who want to know every detail about every treatment and how it affects them…I'm scared to do that'* (P35)

[Personal preference (for proactive role)]: *'PatientView [portal for accessing patient electronic record]…that's fantastic because I can see things, so your platelets, white [blood cells]…haemoglobin…it's a fantastic bit of information for me prior to my consultant appointment…having that extra information, for me, is very valuable…I can point out…to the consultant…so what's this about…you know, why's that gone up?'* (P28)

[Personal circumstances]: *'your mind is concentrating on other things...is it life threatening…am I going to be able to work anymore…have I got to retire…you've got the financial aspects…making sure your family is looked after…your brain is working on so many different levels…you tend to accept what's [treatment] being given'* (P16)

[Level of support]: *'that's what I miss, because I have to do all my own research…and sometimes I think I just wish I had that person… [for support]'* (P33)

Theme 3: Perceptions of proactive/non-proactive approaches to TDM

During interview, a number of patients articulated views relating to proactive involvement in TDM, within the context of their stated preference for acceptance of haematologists' recommendations,

For peer review only - http://bmjopen.bmj.com/site/about/guidelines.xhtml

while others described factors likely to impede or diminish active engagement in TDM. Factors associated with proactive and non-proactive approaches to decisions are summarised in Figure 2.

Those who chose to be proactive described the need for certain resources (internet, time), skills (retrieving/interpreting relevant information) and a high level of personal commitment. This group also tended to access information from many sources, including UK charities (for example, Myeloma UK and CLL Support Association), national and local support groups, on-line patient forums (mainly US based), clinical nurse specialists (CNSs), and peer reviewed articles. Some patients described how internet access was essential, along with the ability to distinguish between *'authoritative'* and *'dreadful'* websites. Taking time to read about blood/lymphatic systems was seen as a necessary pre-requisite for understanding specific diseases. Patients who sought in-depth information from journal publications tended to have some prior knowledge which facilitated their understanding, up to the point when they encountered *'the buffers'* of what they could comprehend. Even these patients, who were well equipped to retrieve and assimilate information, reached a limit to their capacity to understand information relating to their haematological cancer at a specific juncture; other study participants indicated that they had difficulty understanding much/most of the information they encountered.

'I did intelligent searches…I was confident and used to reading science papers…' (R, P6)

'I had read on the internet all about these different prognostic indexes and stuff…when I was told prognosis was 5 years…I already knew what prognosis meant' (P34)

'I'd be looking at Lancet type papers…PubMed' (P24)

'it turned out the free light chains had rocketed… so that ruled out second stem cell transplant' (P18)

'it's good to know statistics…I wanted to know about survival figures…I realised that I was looking at the population as a whole, but then I thought I'd look at younger people…' (P3)

'it's challenging because you will reach the buffers at some point, when you think, that's just a little too difficult to understand' (R, P6)

Proactive patients described preparing for consultations. Despite busy clinics, patients said doctors rarely made them feel time was constrained, usually invited questions, and took time to respond. Some patients prepared questions beforehand, a few noted the consultant's response(s), with some having relatives acting as 'scribe'. Keeping a record or graph of blood test results, used as a trigger for questions, and maintaining a diary of disease progress and treatments were common. These

For peer review only - http://bmjopen.bmj.com/site/about/guidelines.xhtml

patients sought results from investigations (blood tests, bone marrow biopsies, X-rays, CT scans, etc.) and often asked consultants to explain their significance. They also read about the risks, benefits and possible side effects of treatments, to equip themselves to engage in further discussion. Being prepared to 'speak up' during consultations was said to be important, though was recognized to be easier for some than others: *'you have to be reasonably assertive, which is maybe not my strength, but I force myself in those situations where I know how important it is'* (P3). Only one patient (P6) and their spouse (R,P6) reported 'speaking up' to express a preference for a treatment other than the one offered by their consultant, based on their own *'research'*, that suggested it would be less detrimental to the patient's quality of life: *'we knew the gold standard was treatment with Fludarabine, FCR as they call it, it really wasn't recommended for people aged over 65, but nonetheless it was on offer to us…we actually then chose the Bendamustine route…the gentle one…which was a good alternative…we'd done our research, we made an intelligent decision'* (R, P6).

Many respondents expressed little or no desire to engage in active TDM, preferring to rely on clinicians' expertise, as reflected, for example, in the following comments: '*I don't want to go into details of things…the people at the hospital are there to help me…I put my absolute trust in them'* (P10); *'I went along with everything that I was asked to do because I have complete confidence in how they were handling the situation for me'* (P14).

Factors that could impede patients adopting a proactive approach included difficulties accessing and/or fully understanding information, a disinclination to dwell on disease progression and prognosis *('in denial'*: P35*)*, or patients feeling unwell, anxious, overwhelmed, or unsupported.

'I've got a little tablet but I very rarely use it. I don't like to, I'm not computer literate' (P30)

'the specialist at the hospital said, it's only forty-four, which meant nothing to me, forty-four what? She didn't say what and I never asked her.' (P31)

'sometimes they come out with all these big words and then you think, I'm not sure what that word is' (P24)

'I have glanced through it [booklet about myeloma] but it's a bit too high a level…it needs to be basic' (P21)

For peer review only - http://bmjopen.bmj.com/site/about/guidelines.xhtml

'I saw this research about myeloma…and when I read the title I wasn't sure whether it was going to be that helpful, because I didn't understand what it was really…' (P35)

'psychologically, I brush it under the carpet a bit because I know it [chemotherapy] is not imminent' (P5)

'I've just gone back to being anxious again…it's just horrible being in this position where you know it's [paraprotein level] is creeping up' (P35)

'I am strong, you know, but it just becomes too much… I want somebody just to hold my hand and go, I'm going to sort that for you.' (P33)

Theme 4: Experiences of TDM at various points in the disease trajectory

Patients' accounts indicated that the potential and desire for involvement in TDM could vary or indeed be curtailed at different points in the disease trajectory. Most participants described feeling deeply shocked, upset and anxious when first informed of their diagnosis (*'like a huge bombshell'*), to the extent that they could not absorb what was being said to them or think of questions to ask (*'it's as if your brain switched off'*), reactions likely to compromise ability to participate in discussions about treatment options.

'we were floored…[by diagnosis]…it hit us out of the blue…you can't think of questions in that short space of time…we were speechless…we just sat there in shock…you hear the word cancer…'' (P32)

'it was a shock when he mentioned the word cancer…it just sort of shut me down…he was willing to give me information but at that time, I just couldn't process it' (P4)

Some patients on W&W, who subsequently went on to require treatment, reported little or no involvement in TDM about the type of therapy to be given, as there was only a single relevant option. In this context the decision was said to be *'automatic'* and that it would be the *'standard'* treatment. Some of these participants said they would have welcomed having more time to discuss the proposed treatment with family members, and to consider whether or not to accept it; a few recalled their consultant strongly recommending that they accept the treatment offered.

'It was a fait accompli really' (P15)

For peer review only - http://bmjopen.bmj.com/site/about/guidelines.xhtml

'I was told that I had to have chemotherapy, 6 chemotherapies…one every 3 weeks and radiotherapy after that' (P8)

'I was very worried and scared and thought I might choose not to have the treatment…because I felt physically very well…why subject myself to being ill' (P1)

'[consultant said] "I must tell you that even though you don't want to go on chemo….my recommendation is that you do"' (P9).

Opportunities for involvement in decision making appeared circumscribed when urgent treatment was required. Examples came from P19, who was admitted to hospital acutely ill and subsequently diagnosed with FL, from P24, who said he was diagnosed with *'a very unusual lymphoma',* and from P21, with myeloma. In these instances, each patient referred to TDM occurring at speed, largely without their involvement, as they relied on the expertise of the clinical team to make the *'best'* decisions on their behalf.

'There weren't really any options…it was a case of this is what is best for you… I didn't have time to think about it… it [the cancer] was so advanced…they decided… I don't think I was really involved in that…I wanted them to guide me and make the decisions' (P19)

'it was a very unusual type of lymphoma…there was a huge team involved in it all and even throughout their decision making they changed from what were originally going to do, which was radiotherapy...they were just going to blitz it, but my kidney would have been in the way, so they then decided to go down the avenue of chemotherapy and the monoclonal…' (P24)

'they [doctors] decided on stem cell transplant straightaway' (P21)

Disease progression resulted in some patients with myeloma feeling overwhelmed when faced with difficult treatment decisions, and unable to choose between options. Factors compounding this included the intensive nature of proposed treatments (such as stem cell transplant) and their impact on quality of life; the limited "returns" that some treatments seemed to offer, compared to the consequences of associated risks, such as infection; and the uncertainty and unpredictability of outcomes.

'Honestly, my head is exploding with all this…it's just like a big crushing thing to me…I think I am quite strong but this is doing me in' (P35)

For peer review only - http://bmjopen.bmj.com/site/about/guidelines.xhtml

'the big one [decision] was the second stem cell transplant…I was really struggling to make the decision as to whether I wanted to go for it' (P18)

'they [doctors] sort of said, well the average remission after the stem cell transplant is, I think either 12-24 months, or 18-24 months, something like that, and there was I thinking, right I'm going for the 10-year option, so that was quite a shock' (P18)

'Myeloma is a very individual disease. You get the same treatment, same, same this, same that but you have different outcomes and things.' (P28)

Theme 5: Support from others

Our interviewees said that their relatives often accompanied them to clinical consultations, and they were portrayed as playing an important, and sometimes crucial, role when treatment options were being discussed and considered: *'she [patient's wife] guided me…she would translate…she would talk to the ward people…'* (P7). Patients benefitted from *'going over'* information from their haematologist with their relative following consultations, and discussing the details of any proposed treatment, side effects, and implications for quality of life with them: *'we'd come home and discuss it [information from the consultant] what she said about so and so…having two sets of ears helped'* (P11). Some patients (for example, those who mentioned difficulties processing and retaining information) described relying heavily on support from relatives (spouse/partner/adult child/sibling) for all interactions with clinicians: *'my daughter always takes notes…so when we come away we can go through them…they are quite happy with that…if I go on my own, I would retain some of it and I'd probably forget some of it…my daughter knows all about my treatment'* (P9).

Relatives' roles encompassed gathering and interpreting information, acting as a sounding board, and providing practical and emotional support to patients preparing for, and undergoing, treatment.

*'we were proactive and looked for information' (*R,P7)

'we'd [patient and partner] talked through it [decision related to stem cell transplant] over a number of weeks really…we'd come back to it…there's this factor and that factor…' (P18)

'there are times when my wife has come to the rescue…particularly if I get an infection…I'm thankful there is somebody else around' (P11)

For peer review only - http://bmjopen.bmj.com/site/about/guidelines.xhtml

Many patients and relatives used the pronoun *'we'* throughout the interview, though there was general agreement that the final decision of whether or not to accept a treatment, was/would be the patients.

'we'd both be involved…it wouldn't be one partner on their own, it would always be the two of us involved together' (P21)

'he [husband] doesn't sway me, he leaves it very much up to me, he wouldn't persuade me…' (P1)

'I'm as involved as much as I can be but at the end of the day it's [patient's name]… he has to make the decision, I can't make it for him' (R, P31)

'she [partner] kind of left me to make the decision really, but we'd talked through it over a number of weeks' (P18)

The importance of emotional support provided by a spouse/partner to patients experiencing anxiety and inner turmoil associated with diagnosis and TDM was repeatedly emphasised: *'she's [patient's wife] been with me at every step'* (P27); *'you need somebody to be the rock…to take the strain off you'* (P16); *'I don't know how I could have coped without him [husband]'* (P6).

Three study participants living alone commented that they often felt unsupported in relation to their cancer, feelings that were heightened when treatment decisions arose: *'I knew chemo was what I'd have to have but when he* [consultant] *told me, that shocked me, and I was so upset and scared…really scared'* (P4). In the absence of a spouse/partner they turned to friends or close family members for advice and support, but did not want to be a burden to them: *'I've got a very good friend…we've known each other 50 years…I would never have got through it without her…you need support…somebody talk to…but she has her own family…I don't like to be a burden to anybody'* (P8). Two of the three participants without a spouse/partner mentioned seeking formal psychological support, so that they might have the opportunity to share and discuss their feelings and experiences.

Eight of the 35 participants had joined a formal support group. Reported benefits included hearing about up-to-date research findings from invited speakers (often clinicians), and having the opportunity to talk to other people about their experiences, which helped some patients think through advantages and disadvantages of their own options for treatment. Those who preferred not to join a group *('not for me'), ('I'm a very private person'),* often sought one-to-one support, through meetings with former patients arranged by clinicians, personal contacts with someone with a similar diagnosis, and/or via on-line patient forums.

For peer review only - http://bmjopen.bmj.com/site/about/guidelines.xhtml

DISCUSSION

This qualitative study provides new insights into patient perspectives of involvement in treatment decisions, their views on proactive engagement, and the role of others in supporting decision making. The findings reflect the broad array of interconnected mechanisms at play in shared decision making [38], and its complex and dynamic nature [39-41]. The principal finding among our interviewees was the strong preference for treatment recommendations to be provided by haematologists, based on trust in their clinical expertise and perceptions of empathetic patient/clinician relationships. Most participants expected/wanted an explanation from their clinician about the rationale for treatment decisions, including details of possible risks and benefits, but did not wish, and/or felt ill-equipped, to make decisions on their own behalf. This finding does not align with some recent reports of the growing desire for involvement amongst patients with haematological malignancies [20-22], though seems unsurprising within the detailed contexts depicted by our interviewees. Participants with myeloma, who had most decisions to make due to the nature of their cancer, tended to experience decision making as challenging, struggling to weigh up the risks, adverse impacts on quality of life, and prognostic uncertainty associated with different treatments options; which left them inclined to follow clinician recommendations. Patients with FL also reported trusting clinicians to make decisions on their behalf, particularly when they were acutely ill at diagnosis (as may occur in some of these typically indolent cancers), and unable to participate in discussions. Likewise, participants with CLL were mainly inclined to entrust decisions to clinicians; however, some expressed dissatisfaction that they had not been given the opportunity to fully consider, and accept, or decline, treatment. Dissatisfaction amongst this group of patients was possibly, in part, attributable to the fact that patients with CLL often had infrequent contact with haematology HCPs during long periods of observation, with months sometimes elapsing between appointments.

Drawing on results from their systematic review of the literature relating to physician views of SDM, Pollard et al. [42] comment that physicians tend to express support for SDM in situations where they do not feel strongly about one treatment alternative, but are less supportive of SDM in situations where compelling, or well-evidenced, clinical practice guidelines exist in favour of one treatment over another. In such instances, the decision is not one of selecting between options, but rather whether the patient chooses to accept or decline treatment. This review includes results from an interview study with 20 physicians working in five different settings [43], that found support for SDM was most common among those who had received communication skills training in this area; and Rocque et al. [44] suggest that multi-level education programmes, targeting patients with CLL and their clinicians, may improve patient participation in decision making.

For peer review only - http://bmjopen.bmj.com/site/about/guidelines.xhtml

Patient involvement in decision making has been linked to improved care experiences and better health outcomes, yet the desire for this has been shown to vary by individuals, number of treatment options and treatment certainty [45]. That patients with haematological malignancies, which are characterised by uncertain trajectories, indistinct transitions and prognostic uncertainty [46, 47, 7] and novel and evolving treatments, may prefer to defer decisions to specialists, whom they trust, is understandable. Our findings resonate with qualitative studies in Germany [48] and Denmark [49], that combine interviews with extensive observation of consultations with clinicians and patients with cancer, and which show that most of the time physicians made treatment decisions alone, or with colleagues, with little patient involvement. None of our participants recalled clinicians formally eliciting their preferences regarding decision-making, as is recommended [50, 51], yet most felt as involved in this process as they wanted to be, through discussions of treatment options, and clinicians taking time to talk and listen to them, address their concerns, answer questions, and offer explanations. Many of the patients in our study clearly felt strong bonds with their specialists, as noted elsewhere [47], arising from sustained contact in clinic or during hospital admissions. A meta-ethnography of quantitative and qualitative studies [52] underscores the importance patients place on being in a caring relationship with clinicians, which may preclude the need to seek detailed information.

Amongst our participants was a small number (n=5) who shared certain characteristics (male, (mainly) younger, educated to degree level, and highly motivated), and who had adopted a proactive approach to TDM. These individuals generally felt confident interpreting complex information, and were prepared to 'speak up' to obtain further explanation from clinicians; nonetheless, limits to understanding were perceived as constraining their ability to make fully informed decisions. Loh et al. [53] caution against predicting preference for decisional involvement of patients with haematological cancers, based on age or characteristics such as educational attainment, suggesting instead that this should be assessed periodically, as part of decision-making encounters.

While many respondents preferred little or no involvement in TDM, there were some whose engagement was hindered by the provision of information that did not match their needs, leaving them feeling ill-equipped to deal with the complex nature of this material. Clinician assessment of individual health literacy (capacity to access, process and interpret information) is therefore an essential component of SDM, as is noted by others [15]. Providing patients with information that is comprehensible, tailored to their needs, and which does not overwhelm [54], can be challenging. Strategies for 'drip-feeding' [54] information, are to be preferred to a one-way-flow of information from clinician to patient ('broadcasting'), which is regarded as suboptimal [55]. Interestingly,

For peer review only - http://bmjopen.bmj.com/site/about/guidelines.xhtml

insufficient time for information sharing and discussion during consultations was not generally perceived as a problem in our study, though is reported elsewhere [15, 48, 20].

During interview, patients often recalled feelings of profound shock on hearing their diagnosis, reactions that could act as a barrier to meaningful discussion about treatment. A qualitative study [56] including 32 patients with acute myeloid leukaemia revealed similar findings; highlighting the importance of clinicians managing the amount of information patients are ready to receive [54], both at diagnosis and other timepoints; for example, initiation of treatment after W&W.

The important role played by relatives and others in supporting patients during consultations when treatment options are discussed was very apparent in our study, reflecting results from a systematic review [57] of patient-physician-companion communication that shows companions as instrumental in information transfer and provision of emotional support. Unaccompanied patients in our study said they particularly benefitted from a nurse taking notes of what was said during consultation, and 'talking through' the record with them afterwards. Interviewees without a spouse/partner expressed concerns about burdening friends and family members with their needs for practical and emotional support. While some patients may be reluctant to broach the topic of their cancer with others, or find it difficult to talk about it, they are likely to benefit from discussing treatment options/decisions with someone close to them, who is familiar with their personal preferences and circumstances; haematology doctors and nurses could take time to help patients identify such individuals, and encourage patients to draw on their support.

Coping with disease progression and prognostic uncertainty was said to be particularly difficult by our participants with myeloma. Treatment with SCT (or the newer CAR T-cell therapy) can affect patients physically, psychologically and financially [58-60], and formal psychological support may be beneficial [61, 62]. Several of those considering SCT valued one-to-one support, as also noted by Tariman [20]. Furthermore, it has been suggested that information about 'personal experiences' can complement 'general facts', and contribute to decision support in various ways, for example, by helping people clarify their own values and reasoning, either by suggesting different ways of thinking and/or by providing a 'sounding board' against which to test their own ideas [63].

Strengths and Limitations

As far as we are aware, this is the first UK study to specifically explore involvement in TDM in patients with haematological malignancies. Our sample size of 35 enabled in-depth exploration of the research questions, and use of semi-structured interviews allowed participants to focus on issues they themselves considered significant. Our sampling framework ensured 'key informants' were

For peer review only - http://bmjopen.bmj.com/site/about/guidelines.xhtml

interviewed from targeted disease subtypes, both sexes and various age-groups. As the diseases included are typically relapsing-remitting conditions, we felt it important to include patients whose perceptions may have altered over time, during prolonged W&W or following treatment, to capture as broad a range of views as possible. To counteract the influence of memory, we also invited some recently diagnosed patients to take part; reference to patient diaries and contributions from relatives also enhanced recall. Relatives' participation enhanced the quality of the data collected, through contribution of their own perspectives, prompting patients, and, on occasion, corroboration and/or clarification of patients' accounts.

Attempts to recruit patients from black and minority ethnic (BAME) backgrounds were unfortunately unsuccessful. As Morse [64] has highlighted how merging data from a small number of BAME participants can result in loss of cultural differences when analysed alongside the remainder, who share a single identity, we recommend future in-depth studies, dedicated to those whose heritage differs from participants in our own study. Furthermore, fewer people living in more deprived areas agreed to take part, compared to those in affluent areas. Consequently, a further limitation was our inability to recruit patients with low levels of literacy, who may have been deterred because the study invitation and information was provided in writing. We therefore recognise that the views of our participants are unlikely to reflect those of the entire population with the diseases of interest. Nonetheless, it is highly likely that a large proportion of our findings are transferable to other UK areas, and also countries with similar health-care infrastructure and universal health-care coverage.

Clinical Implications

Our findings suggest that our interviewees varied in their preference for involvement in TDM according to intrinsic, contextual, and disease-related factors, requiring clinicians to assess individuals' preferences for engagement at multiple time points over the course of their haematological cancer pathway. Fisher et al. [41] comment that clinicians who clarify patients' preferences and ensure they are informed about their options, are sharing the deliberation aspect of decision making, even if the doctor ultimately provides a strong recommendation. Entrusting clinical staff to make recommendations does not appear to diminish patients' desire for discussion of possible options, and for provision of relevant information that matches their individual needs. Empathetic relationships with clinicians seem highly valued by patients, and appear conducive to engagement in TDM. Deliberation of treatment options can be highly distressing for some patients, and those lacking support from family members/others may benefit from formal assessment and referral for psychological support.

For peer review only - http://bmjopen.bmj.com/site/about/guidelines.xhtml

For peer review only

CONCLUSION

This study revealed that patients with haematological cancers may wish to be involved in TDM to varying extents, contingent on complex, inter-related factors, that are dynamic and subject to change according to clinical and personal contexts. Overall, our interviewees expressed a strong preference for acceptance of clinician recommendations, linked to disease complexity, patients' trust in clinician expertise, and perceptions of trusted patient-clinician relationships.

For peer review only

For peer review only - http://bmjopen.bmj.com/site/about/guidelines.xhtml

Abbreviations and Declarations

Abbreviations

TDM: Treatment decision making; NHS: National Health Service; UK: United Kingdom; P: Patient; R: Relative.

Declarations

Ethics approval and consent to participate

Research projects set within HMRN's infrastructure and collecting supplementary data require ethical approval, which was granted by the London, City and East committee (REC:16/ LO/0740). Assurances of confidentiality and anonymity were given to participants and written consent was obtained from patients and relatives.

Consent for publication

All interviewees consented to the use of quotations from their interviews in publications arising from the study.

Availability of data and materials

All data and materials relating to this research are from the Haematological Malignancy Research Network and are archived and maintained by the first and last author, according to organisational and ethical regulations. Data are not publicly available due to the risk of participant identification from specific contexts revealed when reading entire transcripts and due to the terms and conditions regarding the release of data to third parties upon which ethical approvals for this study were contingent. Reasonable requests for further information relating to this data can be made to the corresponding author.

Competing interests

The authors declare that they have no competing interests.

Funding

This work was supported by the NIHR via a PGfAR: RP-PG-0613-2002, Cancer Research UK: 29685, and Blood Cancer UK (formerly Bloodwise): 15037. None of the funding bodies were involved in the design of the study, nor in the collection, analysis, interpretation and reporting of data; the views expressed here are those of the authors and do not necessarily reflect those of the funder.

Authors' contributions

DH, ER, AS and RP designed the study. AS identified potential participants and mapped pathways. DH and DM recruited the study participants, and DM conducted interviews. Transcripts were coded and analysed by DM with discussion/input from DH. DM wrote the first draft of the manuscript. DH, ER, AS revised the manuscript. RP commented on the clinical aspects of the study. All authors read and approved the final version.

Acknowledgments

We wish to thank the study participants who took part in an interview and shared sensitive and emotive issues.

For peer review only - http://bmjopen.bmj.com/site/about/guidelines.xhtml

For peer review only

REFERENCES:

1. Office for National Statistics. Cancer Registration Statistics, England: 2017. https://www.ons.gov.uk/peoplepopulationandcommunity/healthandsocialcare/conditionsanddiseases/bulletins/cancerregistrationstatisticsengland/2017 (release date April, 2019) Accessed 15 Jan 2021.

2. Public Health England National Statistics Cancer Registration Statistics, England: final release, 2018. Gov.UK (Published 29 May, 2020). https://www.gov.uk/government/publications/cancer-registration-statistics-england-2018-final-release/cancer-registration-statistics-england-final-release-2018. Accessed 15 Jan 2021.

3. Blood Cancer UK, 2018. https://bloodcancer.org.uk/news/latest-statistics Accessed 15 Jan 2021.

4. National Institute for Clinical Excellence (NICE) Interactive Flowcharts. 2020. https://pathways.nice.org.uk/pathways/ Accessed 15 Jan 2021.

5. British Society for Haematology. Guidelines. 2020. https://b-s-h.org.uk/guidelines/ Accessed 15 Jan 2021.

6. Smith A, Howell D, Patmore R, Jack A, Roman E. Incidence of haematological malignancy by sub-type: a report from the Haematological Malignancy Research Network. Br J Cancer. 2011;105:1684–92. Available from: https://doi.org/10.1038/bjc.2011.450.

7. Smith A, Howell D, Crouch S, Painter D, Blase J, Wang H-I, et al. Cohort Profile: The Haematological Malignancy Research Network (HMRN): a UK population-based patient cohort. Int J Epidemiol. 2018; 7:700-700g. Available from: https://doi.org/10.1093/ije/dyy044.

8. Im A, Pavletic SZ. Immunotherapy in hematologic malignancies: past, present, and future. J Hematol Oncol 2017;10:94. Available from: https://doi.org/10.1186/s13045-017-0453-8.

9. Gauthier J, Yakoub-Agha I. Chimeric antigen-receptor T-cell therapy for hematological malignancies and solid tumors: Clinical data to date, current limitations and perspectives. Curr Res Transl Med.2017;65:93-102. Available from: https://doi.org/10.1016/j.retram.2017.08.03.

10. Institute of Medicine (US) Committee on Quality of Health Care in America. Crossing the Quality Chasm: a new Health System for the 21st Century. Washington (DC): Natl Acad Press (US); 2001. Available from: https://doi.org/10.17226/10027.

For peer review only - http://bmjopen.bmj.com/site/about/guidelines.xhtml

11. Coulter A, Collins A. Making Shared Decision Making a Reality: No decision about me, without me. Report from The King's Fund. 2011; London: The King's Fund. Available from: https://www.kingsfund.org.uk/sites/default/files/Making-shared-decision-making-a-reality-paper-Angela-Coulter-Alf-Collins-July-2011_0.pdf. Accessed 15 Jan 2021.

12. Barry M, Edgman-Levitan S. Shared Decision Making – the Pinnacle of Patient-Centred Care. New Engl J Med. 2012;366(9):780-1. Available from: https://doi.org/10.1056/NEJMp1109283.

13. Elwyn G, Frosch D, Thomson R, Joseph-Williams N, Lloyd A, Kinnersley P, Cording E, Tomson D, Dodd C, Rollnick S, Edwards A, Barry M. Shared Decision Making: A Model for Clinical Practice. J Gen Intern Med. 2012;27(10):1361–7 Available from: https://doi.org/10.1007/s11606-012-2077-6.

14. Elwyn G, Durand MA, Song J, Aarts J, Barr PJ, Berger Z, et al. A three-talk model for shared decision making: multistage consultation process. BMJ. 2017;359:j4891 Available from: http://dx.doi.org/10.1136/bmj.j4891

15. LeBlanc TW, Baile WF, Eggly S, Bylund CL, Kurtin S, Khurana M, et al. Review of the patient-centered communication landscape in multiple myeloma and other hematologic malignancies. Pat Educ Couns. 2019;102:1602-1612. Available from: https://doi.org/10.1016/j.pec.2019.04.028.

16. Mulley AG, Trimble C, Elwyn G. Stop the silent misdiagnosis: patients' preferences matter. BMJ. 2012;345:e6572. Available from: https://doi.org/10.1136/bmj.e6572.

17. Elwyn G, Cochran N, Pignone M. Shared Decision Making – the Importance of Diagnosing Preferences. JAMA Intern Med. 2017;177(9):1239-1240. Available from: https://doi.org/10.1001/jamainternmed.2017.1923.

18. Bomhof-Roordink H, Fischer MJ, van Duijn-Bakker N, Baas-Thijssen MC, van der Weijden T, Stiggelbout AM, Pietersei AH. Shared decision making in oncology: A model based on patients', healthcare professionals' and researchers' views. Psycho-Oncology. 2019;28 (1):139–146. Available from: https://doi.org/10.1002/pon.4923.

19. Alexander SC, Sullivan AM, Back AL, Tulsky JA, Goldman RE, Block SD, et al. Information giving and receiving in haematology malignancy consultations. Psycho-Oncology. 2012;21(3):297–306. Available from: https://doi.org/10.1002/pon.1891.

20. Tariman JD, Doorenbos A, Schepp KG, Becker PS, Berry DL. Patient, Physician and Contextual Factors Are Influential in the Treatment Decision Making of Older Adults Newly Diagnosed with

For peer review only - http://bmjopen.bmj.com/site/about/guidelines.xhtml

Symptomatic Myeloma. Cancer Treat Commun. 2014;2:34-47. Available from: https://doi.org/10.1016/j.ctrc.2014.08.003.

21. Rood JAJ, van Zuuren, FJ, Stam F, van der Ploeg T, Eeltink C, Verdonck-de Leeuws IM, Huigens PC. Perceived need for information among patients with a haematological malignancy: associations with information satisfaction and treatment decision-making preferences. Hematol Oncol. 2015;33 (2): 85–98. Available from: https://doi.org.10.1002/hon.2138.

22. Rood JAJ, Nauta IH, Witte BI, Stam F, van Zuuren FJ, Manenschijn A, et al. Shared decision-making and providing information among newly diagnosed patients with haematological malignancies and their informal care-givers: Not "one size fits all". Psycho-Oncology. 2017;26(12):2040–2047. Available from: https://doi.org/10.1002/pon.4414.

23. Tong A, Sainsbury P, Craig J. Consolidated criteria for reporting qualitative research (COREQ): a 32-item checklist for interviews and focus groups. Int J Qual Health Care. 2007; 19(6):349-57. Available from: https://doi.org/10.1093/intqhc/mzm042.

24. Sandelowski M. 2000. Focus on research methods: Whatever happened to qualitative description? Res Nurs Health. 23(4):334-340. Available from: https://doi.org/10.1002/1098-240x(200008)23:4<334::aid-nur9>3.0.co;2-g.

25. Neergaard MA, Olesen F, Andersen RS, Sondergaard J. Qualitative description-the poor cousin of health research? BMC Med Res Methodol. 2009;9:52. Available from: https://doi.org/10.1186/1471-2288-9-52.

26. Gentles SJ, Charles C, Ploeg J, McKibbon KA. Sampling in Qualitative Research: Insights from an Overview of the Methods Literature. The Qualitative Report. 2015; 20:1772-1789. Available from: http://nsuworks.nova.edu/tqr/vol20/iss11/5

27. Malterud K, Siersma VD, Guassora AD. Sample size in qualitative interview studies: Guided by information power. Qual Health Res. 2016; 26(13):1753–1760. Available from: https://doi.org/10.1177/1049732315617444.

28. Saunders B, Sim J, Kingstone T, Baker S, Waterfield J, Bartlam B, et al. Saturation in qualitative research: Exploring its conceptualization and operationalization. Qual Quant. 2018; 52(4):1893–1907. Available from: https://doi.org/10.1007/s11135-017-0574-8.

For peer review only

For peer review only - http://bmjopen.bmj.com/site/about/guidelines.xhtml

29. Thorne, S. The Great Saturation Debate: What the "S Word" Means and Doesn't Mean in Qualitative Research Reporting. Can J Nurs Res. 2020; 52: 3–5. Available from: https://doi.org/10.1177/0844562119898554.

30. Morse J. Confusing categories and themes. (Editorial). Qual Health Res. 2008;18 (6):727-728. Available from: https://doi.org/10.1177/1049732308314930.

31. Bradshaw C, Atkinson S, Doody O. Employing a Qualitative Description Approach in Health Care Research. Glob Qual Nurs Res. 2017;24 (4):2333393617742282. Available from: https://doi.org/10.1177/2333393617742282.

32. Caelli K, Ray L, Mill, J. "Clear as mud": Toward greater clarity in generic qualitative research. Int J Qual Methods. 2003;2:1–23. Available from: https://doi.org/10.1177/160940690300200201.

33. Pope C, Mays N. (eds). Qualitative Research in Health Care. 3rd ed. Oxford: BMJ Blackwell Publishing; 2006.

34. Sandelowski, M. (2010). What's in a name? Qualitative description revisited. Res Nurs Health. 33(1):77–84. Available from: https://doi.org/10.1002/nur.20362.

35. Green J, Thorogood N. Qualitative Methods for Health Research: Introducing Qualitative Methods Series. 4th ed. London: Sage; 2018, p258.

36. Pope C, Ziebland S, Mays N. Analysing qualitative data. BMJ. 2000;8;320 (7227): 114–116. Available from: https://doi.org/10.1136/bmj.320.7227.114.

37. Barbour RS. 2001. Checklists for improving rigour in qualitative research: a case of the tail wagging the dog? BMJ. 2001;322:1115–1117. Available from: https://doi.org/10.1136/bmj.322.7294.1115.

38. Waldron T, Carr T, McMullen L, Westorp G, Duncan V, Neufeld S-M, Bandura L-A, Groot G. Development of a Programme Theory for Shared Decision Making: a realist synthesis. BMC Health Serv Res. 2020;20(1):59. Available from: https://doi.org/10.1186/s12913-019-4649-1.

39. Entwistle VA, Watt IS. Patient involvement in treatment decision making: the case for a broader conceptual framework. Pat Ed Couns. 2006;63(3):268-278. Available from: https://doi.org/10.1016/j.pec.2006.05.002.

40. Joseph-Williams N, Elwyn G, Edwards, A. Knowledge is *not* power for patients: A systematic review and thematic synthesis of patient-reported barriers and facilitators to shared decision

For peer review only - http://bmjopen.bmj.com/site/about/guidelines.xhtml

making. Pat Educ Couns. 2014.94(3):291-309. Available from: https://doi.org/10.1016/j.pec.2013.10.031.

41. Fisher KA, Tan ASL, Matlock DD, Saver B, Mazor KM, Pieterse AH. Keeping the patient in the center: Common challenges in the practice of shared decision making. Pat Educ Couns. 2018;101(12):2195–2201. Available from: https://doi.org/10.1016/j.pec.2018.08.007.

42. Pollard S, Bansback N, Bryan S. Physician attitudes toward shared decision making: A systematic Review. Pat Educ Couns. 2015;98(9):1046-1057. Available from: https://doi.org/j.pec.2015.05.004.

43. Zeuner R, Frosch DL, Kuzemchak MD, Politi MC. Physicians' perceptions of shared decision-making behaviours: a qualitative study demonstrating the continued chasm between aspirations and clinical practice. Health Expect. 2014;18(6):2465–2476. Available from: https://doi.org/10.1111/hex.12216.

44. Rocque GB, Williams CP, Halilova KI, Borate U, Jackson BE, Van Laar ES, et al. Improving shared decision making in chronic lymphocytic leukaemia through multidisciplinary education. Trans Behav Med. 2018;8:175-182. Available from: https://doi.org/10.1093/tbm/ibx034.

45. Blumenthal-Barby JS. 'That's the doctor's job': Overcoming patient reluctance to be involved in medical decision making. Pat Educ Couns. 2017; 100(1):14-17. Available from: https://doi.org/10.1016/j.pec.2016.07.010.

46. National Institute for Clinical Excellence (NICE). Haematological cancers: improving outcomes NICE guideline [NG47]. https://www.nice.org.uk/guidance/ng47/evidence/improving-outcomes-in-haematological-cancers-the-manual-pdf-2487893581. Accessed 15 Jan 2021.

47. McCaughan D, Roman E, Smith AG, Garry A, Johnson M, Patmore R, et al. Determinants of hospital death in haematological cancers: findings from a qualitative study. BMJ Supp Palliat Care. 2018;8(1):78–86. Available from: https://doi.org/10.1136/bmjspcare-2016-001289.

48. Hahlweg P, Harter M, Nestoriuc Y, Scholl I. How are decisions made in cancer care? A qualitative study using participant observation of current practice. BMJ Open. 2017;7(9):e016360. Available from: https://doi.org/10.1136/bmjopen-2017-016360.

49. Lowe MM, Osther PJS, Ammentorp J, Birkelund R. The Balance of Patient Involvement: Patients' and Health Professionals' Perspectives on Decision-Making in the Treatment of Advanced Prostate Cancer. Qual Health Res. 2021;31(1):29-40. Available from: https://doi.org/10.1177/1049732320962759.

For peer review only - http://bmjopen.bmj.com/site/about/guidelines.xhtml

50. Coulter A. Patient information and shared decision-making in cancer care. Br J Cancer. 2003;89 Suppl 1:S15-16. Available from: https://doi.org/10.1038/sj.bjc.6601080.

51. Legare F, Stacey D, and IP TEAM. IP-SDM model. 2010. https://decisionaid.ohri.ca/docs/develop/IP-SDM-Model.pdf Accessed 15 Jan 2021.

52. Atherton K, Young B, Salmon P. Understanding the information needs of people with haematological cancers. A meta-ethnography of quantitative and qualitative research. Eur J Cancer Care. 2017;26(6):e12647. Available from: https://doi.org/10.1111/ecc.12647.

53. Loh KP, Tsang M, LeBlanc TW, Back A, Duberstein PR, Mohile SG, et al., Decisional involvement and information preferences of patients with hematologic malignancies. Blood Adv. 2020; 4(21):5492-5500. Available from: https://doi.org/10.1182/bloodadvances.2020003044.

54. Atherton K, Young B, Kalakonda N, Salmon P. Perspectives of patients with haematological cancer on how clinicians meet their information needs: "Managing" information versus "giving" it. Psycho-Oncology. 2018;27(7):1719–1726. Available from: https://doi.org/10.1002/pon.4714.

55. Chhabra KR, Pollack KI, Lee SJ, Back AL, Goldman RE, Tulsky JA. Physician Communication Styles in Initial Consultations for Hematological Cancer. Pat Educ Couns. 2013; 93(3):573-8. Available from: https://doi.org/10.1016/j.pec.2013.08.023.

56. LeBlanc TW, Fish LJ, Bloom CT, El-Jawahri A, Davis DM, Locke SC, et al. Patient experiences of acute myeloid leukemia: A qualitative study about diagnosis, illness understanding, and treatment decision-making. Psycho-Oncology. 2017;26(12):2063–2068. Available from: https://doi.org/10.1002/pon.4309.

57. Laidsaar-Powell RC, Butow PN, Bu S, Charles C, Gafni A, Lam WWT, et al. Physician–patient–companion communication and decision-making: a systematic review of triadic medical consultations. Patient Educ Couns. 2013; 91(1):3–13. Available from: https://doi.org/10.1016/j.pec.2012.11.007.

58. Mosher CE, DuHamel KN, Rini C, Corner G, Lam J, Redd WH. Quality of Life Concerns and Depression among Hematopoietic Stem Cell Transplant Survivors. Support Care Cancer. 2011; 19(9): 1357–1365. Available from: https://doi.org.10.1007/s00520-010-0958-y.

For peer review only - http://bmjopen.bmj.com/site/about/guidelines.xhtml

59. Walpole G, Clark H, Dowling M. Myeloma patients' experiences of haematopoietic stem cell transplant: A qualitative thematic synthesis. Eur J Onc Nurs. 2018;35:15-21. Available from: https://doi.org/10.1016/j.ejon.2018.05.002.

60. Ruark J, Mullane E, Cleary N, Cordeiro A, Bezzero ED, Wu V, et al., Patient-Reported Neuropsychiatric Outcomes of Long-Term Survivors after Chimeric Antigen Receptor T Cell Therapy. Biol Blood Marrow Transplant. 2020; 26(1):34-43. Available from: https://doi.org/10.1016j.bbmt.2019.09.037.

61. Swash B, Hulbert-Williams N, Bramwell R. Unmet psychosocial needs in haematological cancer: a systematic review. Support Care Cancer. 2014; 22(4):1131-1141. Available from: https://doi.org/10.1007/s00520-014-2123-5.

62. Oberoi DV, White VM, Seymour JF, Prince HM, Harrison S, Jefford M et al. Distress and unmet needs during treatment and quality of life in early cancer survivorship: A longitudinal study of haematological cancer patients. Eur J Haematol. 2017; 99(5):423-430. Available from: https://doi.org/10.1111/ejh.12941.

63. Entwistle VA, France EF, Wyke S, Jepson R, Hunt K, Ziebland S, Thompson A. How information about other people's personal experiences can help with healthcare decision-making: a qualitative study. Patient Educ Couns. 2011;85(3):e291–8. Available from: https://doi.org/10.1016/j.pec.2011.05.014.

64. Morse J. The Changing Face of Qualitative Inquiry. Invited IIQM Conference Keynote address 2019. Int J Qual Methods. 2020; 19:1–7. Available from: https://doi.org/10.1177/1609406920909938.

For peer review only - http://bmjopen.bmj.com/site/about/guidelines.xhtml

Figure 1. Factors implicated in patient involvement in treatment decisions

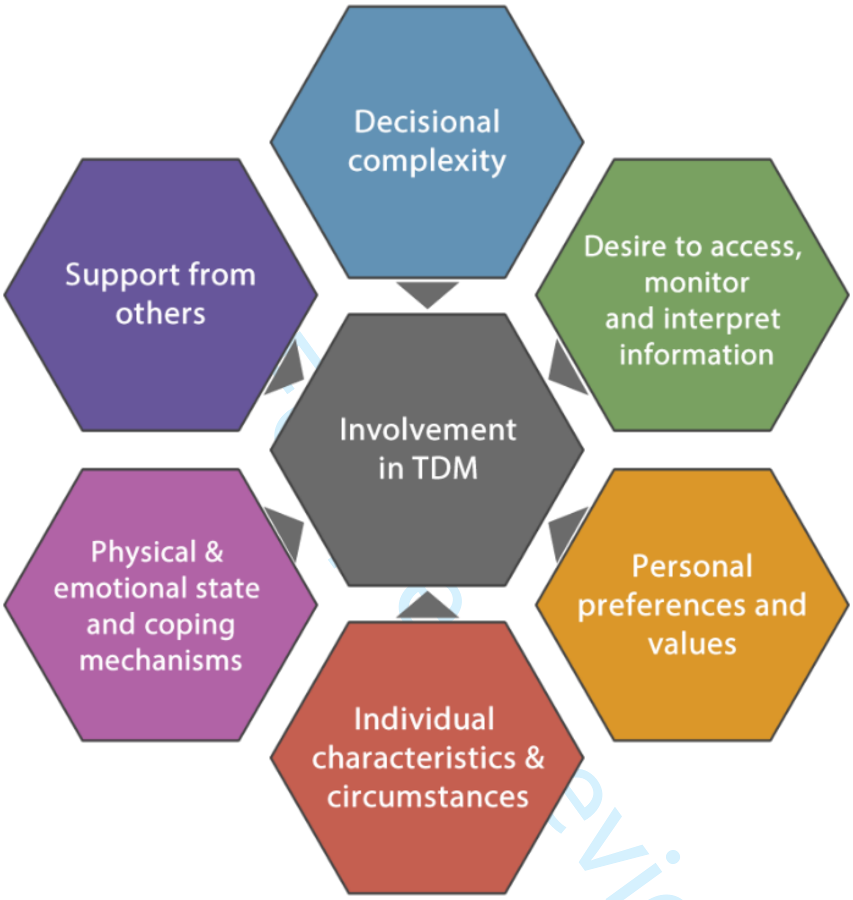

For peer review only

Figure 2. Continuum of characteristics associated with proactive and non-proactive approaches to involvement in treatment decisions

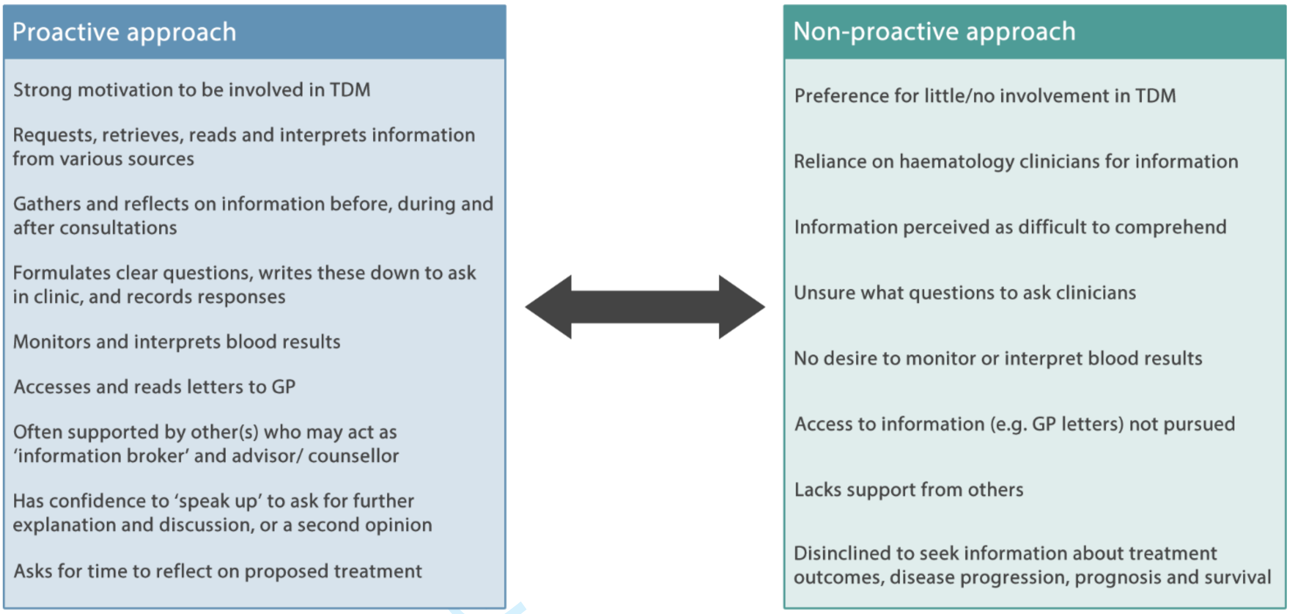

Supplementary file 1: Topic guide for interviews with patients and relatives

Focusing on information and decisions at key states (diagnosis, W&W, treatment) and progression through states.

Information

- How important is it to you that you receive information about your cancer? *(why is that?*)
- How do you feel about the information given to you at diagnosis/start of treatment?
- How do you feel about getting information from HCPs more generally? *(time constraints; overwhelming; difficult to understand/take in; use of language/terminology)*
- Do you feel the information given applies specifically to you? *(personalized, tailored, specific)*
- How healthcare practitioners (HCPs) ascertain your information needs?
- Is the information you received explained in a way you can understand? *(technical language; too detailed; not detailed enough)*
- What do HCPs do to check if you understand the information they give you?
- How do you feel about asking questions? Are your questions always answered?
- Do you feel that your information needs are usually met? What worked well and could have been better? *(diagnosis; treatment initiation/cessation - examples)*
- What do you think about the timing of information from HCPs? When is the right time? *(at diagnosis; during clinic appointments; when disease status changes; at other times)*
- How do/did you feel about discussing the risks/benefits of different treatments with HCPs?
- How do you feel about discussing prognosis? *("a statement about expectations that refers to the likely course of the cancer and/or outcome") (want to know/not; timing; language)*
- What strategies do you use to absorb information? (*in general, how bad news is processed*)

Treatment decisions

- How do you feel about being involved in decisions with HCPs about your treatment?
- Have you been asked you if you want to be involved in decisions about treatment?
- Do you want to be involved in decisions? *(preference for patient only; clinician only; patient/clinician)*
- What should be considered during treatment decision making? *(effectiveness of treatment; side effects; prognosis; patient goals, values, preferences; impact on quality of life)*
- What might make it easier or harder for you to be involved in making decisions about your treatment? *(time; style of communication; how information is conveyed; explanations)*
- Are there particular time-points when it is harder to be involved in making decisions about treatment? *(diagnosis; treatment initiation/change; treatment cessation)*

Practical issues

- How do you feel about the amount of information you get? *(prefer more/less; overloaded; struggle to absorb)*
- What do you want to know/know more about? *(investigations; treatments; prognosis; side effects; QoL)*
- Where/who do you prefer to get information; why? *(Internet; doctors/nurses; family; leaflets; support group)*
- What do you think about different sources of information? *(credibility, ability to judge)*
- How do you prefer to see information about risks and benefits; why? (w*ords/numbers; figures/percentages; diagrams/graphs)*

For peer review only

COREQ (COnsolidated criteria for REporting Qualitative research) Checklist

Topic	No.	Guide Questions/Description	Page	Explanation
**Domain 1: Research team and reflexivity**				
**Personal characteristics**				
Interviewer	1	Which author conducted the interview?	p5	Dorothy McCaughan (DM)
Credentials	2	What were the researcher's credentials? E.g. PhD, MD	n/a	BA, RN MSc
Occupation	3	What was their occupation at the time of the study?	n/a	Senior Research Fellow
Gender	4	Was the researcher male or female?	n/a	Female
Experience and training	5	What experience or training did the researcher have?	n/a	Registered Nurse. Conducted many qualitative interviews patients.
**Relationship with participants**				
Relationship established	6	Was a relationship established prior to the study?	n/a	No relationship existed with participants prior to the study.
Participant knowledge of the interviewer	7	What did participants know about the researcher? e.g. personal goals, reasons for doing the research?	n/a	Participants had no knowledge of the interviewer prior to the study.
Interviewer characteristics	8	What characteristics were reported about the interviewer? e.g. Bias, assumptions, interests in topic	p5	Data were collected by DM, analysed by DM & DH, both qualified nurses and experienced health services researchers.
**Domain 2: Study design**				
**Theoretical framework**				
Methodological orientation and theory	9	What methodological orientation underpinned the study? e.g. grounded theory, content analysis	p5	Qualitative description and thematic content analysis (referenced in the Methods).
**Participant selection**				
Sampling	10	How were participants selected? e.g. purposive, convenience, consecutive,	p4	Interviewees were purposively selected.
Method of approach	11	How were participants approached?	p5	Interviewees were recruited by mail. Potential participants contacted DM if they wanted to take part.
Sample size	12	How many participants were in the study?	p5	Thirty-five patient interviews were undertaken, ten included relatives.
Non-participation	13	How many people refused to participate or dropped out? Reasons?	n/a	After invitation, around 20 patients did not contact the study team; reasons for this are unknown. No-one who responded dropped out.
Setting of data collection	14	Where was the data collected? e.g. home, clinic, workplace	p5	Data were largely collected in patient homes. A small number took place in the University or elsewhere.
Presence of nonparticipants	15	Was anyone else present besides the participants and researchers?	p5	Interviews were conducted privately.

For peer review only

Description of sample	16	What are the important characteristics of the sample? e.g. demographic data, date	p4 Table 1	The sample was selected from HMRN and included patients with chronic haematological malignancies, who had agreed they could be contacted for research (men and women of any ethnicity and age, living in any area).
Interview guide	17	Were questions, prompts, guides provided by the authors? Was it pilot tested?	p5 Appx. 1	A semi-structured topic guide, tested on 2 patients, was developed from the literature and team experiences.
Repeat interviews	18	Were repeat interviews carried out? If yes, how many?	n/a	No, there were no repeat interviews.
Audio/visual recording	19	Was audio or visual recording used to collect the data?	p5	Audio recording was used.
Field notes	20	Were field notes made during and/or after the interview?	n/a	Field notes were made post-interview.
Duration	21	What was the duration of the interviews?	p5	Interviews lasted 40-90 minutes.
Data saturation	22	Was data saturation discussed?	p5	Interviews continued until saturation appeared to have been reached.
Transcripts returned	23	Were transcripts returned to participants for comment and/or correction?	n/a	This was not requested and audiotaping was clear.
**Domain 3: analysis and findings**				
**Data analysis**				
Number of data coders	24	How many data coders coded the data?	p5	DM & DH were involved in coding and discussed/developed the scheme.
Description of the coding tree	25	Did authors provide a description of the coding tree?	p5-6 Fig. 1	The coding scheme (tree) reflected the topic guide and was developed to accommodate data content.
Derivation of themes	26	Were themes identified in advance or derived from the data?	p5	Themes were derived from the data. The topic guide provided a framework for data collection.
Software	27	What software, if applicable, was used to manage the data?	n/a	Data were managed in Word and Excel.
Participant checking	28	Did participants provide feedback on the findings?	n/a	Feedback was provided to patients via the study newsletter.
**Reporting**				
Quotations presented	29	-Were quotations presented to illustrate themes/findings? -Were quotations linked to ID?	p7-16	Quotations are presented with anonymised IDs, linked to more detailed characteristics in Table 1.
Data and findings consistent	30	Was there consistency between data and findings?	p7-16	We consistently link findings with supporting quotations.
Clarity of major themes	31	Were major themes clearly presented in the findings?	p7-16 Fig. 1&2	Five major themes are clearly defined, with separate headings.
Clarity of minor themes	32	Is there a description of diverse cases or discussion of minor themes?	p5, p19	Whilst highlighting the main themes, we also incorporated a range of responses and drew attention to negative cases.

Developed from: Tong A, Sainsbury P, Craig J. Consolidated criteria for reporting qualitative research (COREQ): a 32-item checklist for interviews and focus groups. International Journal for Quality in Health Care. 2007. Volume 19, Number 6: pp. 349 – 357.

For peer review only
